# Chemoenzymatic Halocyclization of γ,δ‐Unsaturated Carboxylic Acids and Alcohols

**DOI:** 10.1002/cssc.201902240

**Published:** 2019-10-22

**Authors:** Sabry H. H. Younes, Florian Tieves, Dongming Lan, Yonghua Wang, Philipp Süss, Henrike Brundiek, Ron Wever, Frank Hollmann

**Affiliations:** ^1^ Department of Biotechnology Delft University of Technology Van der Maasweg 9 2629 HZ Delft The Netherlands; ^2^ Department of Chemistry Faculty of Sciences Sohag University Sohag 82524 Egypt; ^3^ School of Food Science and Engineering Overseas Expertise Introduction Center for Discipline Innovation of Food Nutrition and Human Health (111 Center) South China University of Technology Guangzhou 510640 P.R. China; ^4^ Enzymicals AG Walther-Rathenau-Str. 49a 17489 Greifswald Germany; ^5^ Van't Hoff Institute for Molecular Sciences University of Amsterdam Amsterdam The Netherlands

**Keywords:** biocatalysis, enzymes, etherification, lactones, haloperoxidases

## Abstract

A chemoenzymatic method for the halocyclization of unsaturated alcohols and acids by using the robust V‐dependent chloroperoxidase from *Curvularia inaequalis* (*Ci*VCPO) as catalyst has been developed for the in situ generation of hypohalites. A broad range of halolactones and cyclic haloethers are formed with excellent performance of the biocatalyst.

## Graphical abstract

**You had me at “halo”**: A chemoenzymatic method for the halocyclization of unsaturated alcohols and acids by using a robust V‐dependent chloroperoxidase as catalyst has been developed for the in situ generation of hypohalites. A broad range of halolactones and cyclic haloethers are formed with excellent biocatalyst performance.

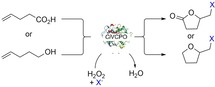

Halolactonization reactions are well‐established in organic synthesis.^1^ The established synthetic routes use a variety of catalysts and halide sources. *N*‐Bromosuccinimide, for example, is commonly used.^2^ The resulting byproduct, however, is often difficult to recover from the reaction mixture and ends up as waste. Moreover, elementary halides are used, which poses questions of safety and corrosion.^3^ Recently, Oxone was proposed as an alternative means of producing electrophilic bromine species from bromide.^4^ Although this method avoids organic waste, it still produces significant amounts of inorganic salts (sulfates) as waste. Other catalytic methods to generate BrO^−^ rely on catalysts such as organic tellurides,^5^ selenides,^6^ or Cu catalysts.^7^ Haloetherification of alkenols is similarly difficult to achieve.^8^

Haloperoxidases (E.C. 1.11.1) represent an interesting alternative to the aforementioned chemical means to generate electrophilic halide species from halides and hydrogen peroxide under mild reaction conditions. In particular, the V‐dependent chloroperoxidase from *Curvularia inaequalis* (*Ci*VCPO) is a very promising catalyst with exceptional activity and stability.^9^ Previously, we have applied this enzyme for the halogenation of phenols,^10^ hydroxyhalogenation of alkenes,^11^ and, inspired by the pioneering work by Deska and co‐workers,^12^ to mediate (aza‐)Achmatowicz reactions.^13^ In the current study, we investigated the suitability of *Ci*VCPO to initiate the spontaneous halolactonization of γ,δ‐unsaturated carboxylic acids (Scheme 1).

**Scheme 1:**
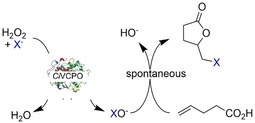
Halolactonization of 4‐pentenoic acid (as model δ,γ‐unsaturated carboxylic acid) with hypohalites generated from H_2_O_2_ and halides using the V‐dependent chloroperoxidase from *Curvularia inaequalis* (*Ci*VCPO).

First, we evaluated the influence of several reaction parameters, such as pH and reagent concentration, on the efficiency of the bromolactonization of 4‐pentenoic acid. In accordance with our previous findings,^10^, ^11^, ^13^ the reaction proceeded optimally at pH 5 (with more than 80 % activity at both pH 7 and pH 4; Table 1). Although this behavior can most likely be attributed to the pH‐dependency of the biocatalyst, the protonation stage of the carboxylate group may also play a role here. Reactions in non‐buffered media were less efficient, most probably owing to the alkalization of the reaction medium in the course of the reaction. The concentrations of bromide and H_2_O_2_ both directly influenced the conversion of the reaction. Performing the reaction in the absence of the biocatalyst did not result in any significant conversion within the timeframe of the experiment.

**Table 1:** Influence of pH and reagent concentration on the chemoenzymatic bromolactonization of 4‐pentenoic acid. 
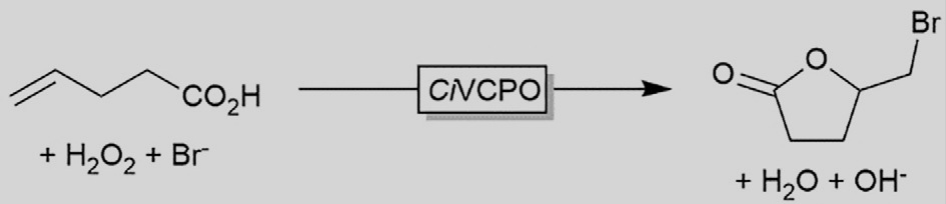

pH	KBr [mm]	H_2_O_2_ [mm]	Conversion [%]
H_2_O^[a]^	160	170	39
3	160	170	40
4	160	170	80
5	160	170	99
7	160	170	90
9	160	170	20
5	160	85	80
5	80	170	40
5^[b]^	160	170	–

General conditions: *c*(4‐pentenoic acid)=40 mm; 100 mm citrate buffer (pH 5); *c*(*Ci*VCPO)=100 nm; *T*=25 °C; *t*=24 h. Other buffers used: acetate (pH 3), citrate (pH 4), potassium phosphate (pH 7) and Tris buffer (pH 9); a: double distilled water, unbuffered; b: reaction performed in the absence of *CiVCPO*.

A typical time course of the chemoenzymatic bromolactonization is shown in Figure 1. Very pleasingly, *Ci*VCPO performed more than 5 catalytic cycles per second and at least 325 000 catalytic cycles.

**Figure 1:**
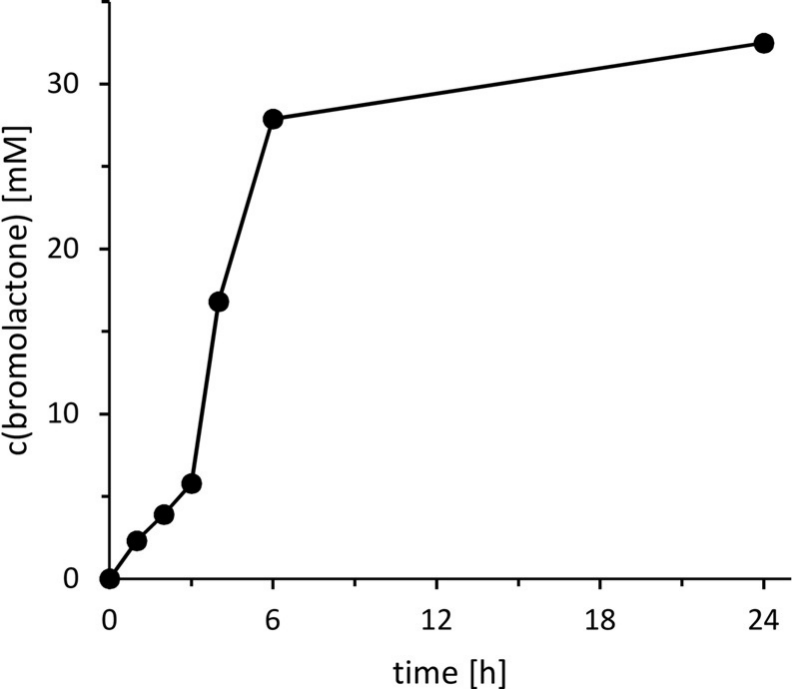
Representative time course of the chemoenzymatic bromolactonization of 4‐pentenoic acid. General conditions: *c*(4‐pentenoic acid)=40 mm; *c*(H_2_O_2_)=170 mm; *c*(KBr)=160 mm; 100 mm citrate buffer (pH 5); *c*(*Ci*VCPO)=100 nm; *T*=25 °C.

Next, we further evaluated the product scope of the chemoenzymatic halolactonization reaction (Table 2). Pleasingly, all starting materials were converted with good to excellent conversions into the corresponding halolactones. In particular, the cyclohexene‐derived (enantiomerically pure) products may serve as building blocks for a range of natural products.^14^ The selectivity of the reaction was generally satisfactory with the corresponding hydroxylactone as the sole byproduct.^15^

**Table 2:** Preliminary product scope of the chemoenzymatic halolactonization of γ,δ‐unsaturated carboxylic acids.

Substrate	Product	Conversion [%]^[a]^ (Selectivity [%])	
		**a**: X=Br	**b**: X=Cl
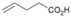	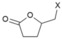	>99 (67)	>99 (67)
**1**	**9 a,b**		
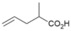	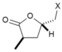	>99 (64)	>99 (65)
**2**	**10 a**,**b**		
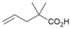	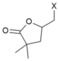	>99 (72)	>99 (68)
**3**	**11 a**,**b**		
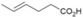	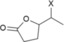	>99 (57)	>99 (56)
**4**	**12 a**,**b**		
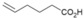	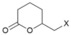	>99 (56)	>99 (70)
**5**	**13 a**,**b**		
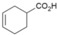	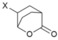	>99 (70)	>99 (62)
**6**	**14 a**,**b**		
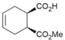	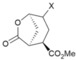	>99 (80)	86 (82)
**7**	**15 a**,**b**		
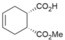	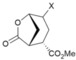	>99 (79)	80 (87)
**8**	**16 a**,**b**		

General conditions: *c*(substrate)=40 mm; *c*(H_2_O_2_)=100 mm; *c*(KX)=160 mm; 100 mm citrate buffer (pH 5); *c*(*Ci*VCPO)=100 nm; *T*=25 °C; *t*=24 h. [a] determined by NMR spectroscopy (see the Supporting Information for spectra and further details).

The relative configuration for product **10 a** was established based on coupling constants and NOE experiments. The NOE correlation between H‐5 (m, dH 4.51–4.48) and H‐6b (dd, dH 3.60, *J*=12.7, 5.0 Hz, 1 H) suggested the same orientation of H‐5 and H‐6b. The NOE correlations between H‐5 and the H‐4b (m, dH 1.97–1.89), as well as the methyl group at 1.25 ppm indicated protons located in the same orientation (see the Supporting Information, Figures S7 and S8).

To demonstrate the preparative feasibility, we performed the chloro‐, bromolactonization of 4‐pentenoic acid and bromolactonization of 2‐methyl‐4‐pentenoic acid at 10 mmol scale. 0.9, 1.4, and 1.15 g of the desired chloro‐ and bromolactone products were isolated corresponding to 70, 80, and 60 % yields, respectively, as well as 0.58 g (30 %) of hydroxylactone in the case of bromolactonization of 2‐methyl‐4‐pentenoic acid.

One apparent drawback of the current chemoenzymatic halolactonization reaction lies with the nonselective chemical step producing racemic lactones. We therefore envisioned complementing the halolactonization reaction with a hydrolase‐catalyzed kinetic resolution step (Scheme 2). In total, 9 commercial and self‐made hydrolases were screened. However, none of the enzymes exhibited an enantioselectivity high enough for efficient kinetic resolution (Figures S56 and S57). Currently, protein engineering of the lipase *Streptomyces* sp.^16^ is ongoing to obtain a more enantioselective and hence, practical catalyst.

**Scheme 2:**
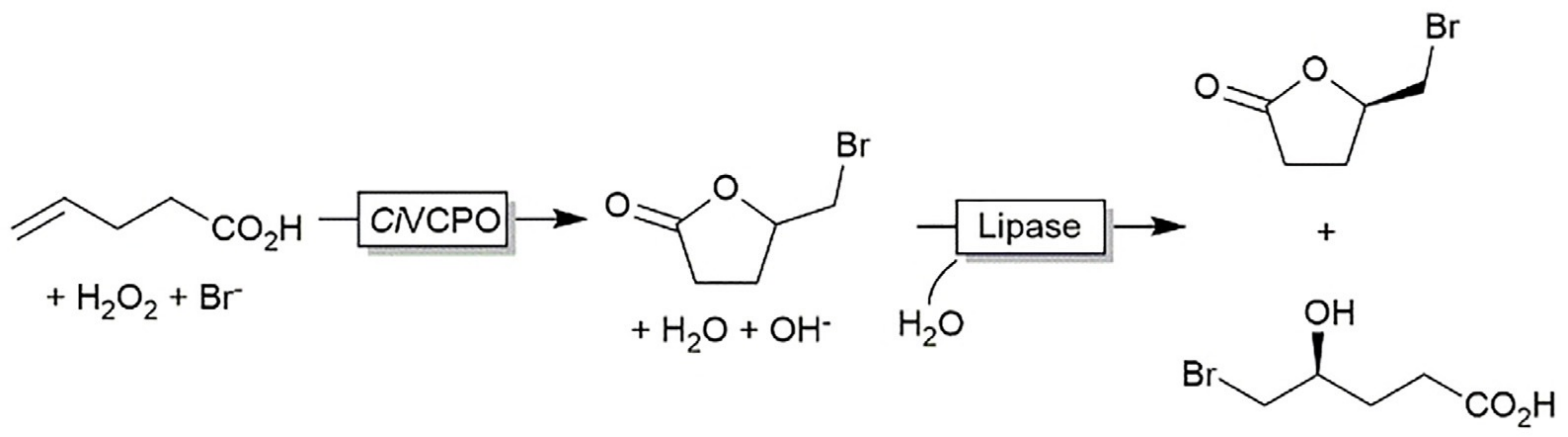
Envisioned kinetic resolution of the racemic lactones obtained from the chemoenzymatic bromolactonization reaction.

Finally, we investigated the possibility of performing haloetherification reactions in the current setup. Assuming the intermediate halonium ion is sufficiently stable under the aqueous conditions, we reasoned that intramolecular etherifications should be feasible (Scheme 3).

**Scheme 3:**
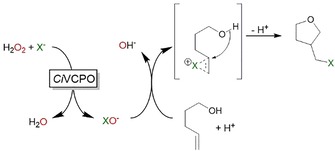
Envisioned chemoenzymatic haloetherification reaction.

The proof‐of‐concept reaction proceeded smoothly to full conversion (Figure 2). Overall 36 mm of 2‐(bromomethyl)tetrahydro‐2*H*‐pyran were obtained within 24 h, corresponding to a turnover number of more than 360 000 for the biocatalyst.

**Figure 2:**
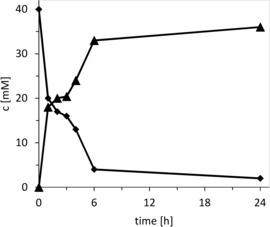
Time course of the chemoenzymatic etherification of 5‐hexen‐1‐ol (♦) into 2‐(bromomethyl)tetrahydro‐2*H*‐pyran (▴). General conditions: *c*(5‐hexen‐1‐ol)=40 mm; *c*(*Ci*VCPO)=100 nm; *c*(H_2_O_2_)=170 mm, *c*(KBr)=160 mm; 100 mm citrate buffer (pH 5); *T*=25 °C.

Indeed, with all commercially available alkenols tested, we found significant formation of the expected cyclic ethers (Scheme 4). As in case of the lactonization reactions, the sole byproducts observed in these reactions were the hydroxyethers (X=OH). The relative configuration of compound **19 a** was determined depending upon NOE correlations. Based on the structure of the starting material (−)‐carveol, the NOE correlation of CH_3_‐2 and H‐3 indicated the positioning of these functional groups on the same side (Figure S27).

**Scheme 4:**
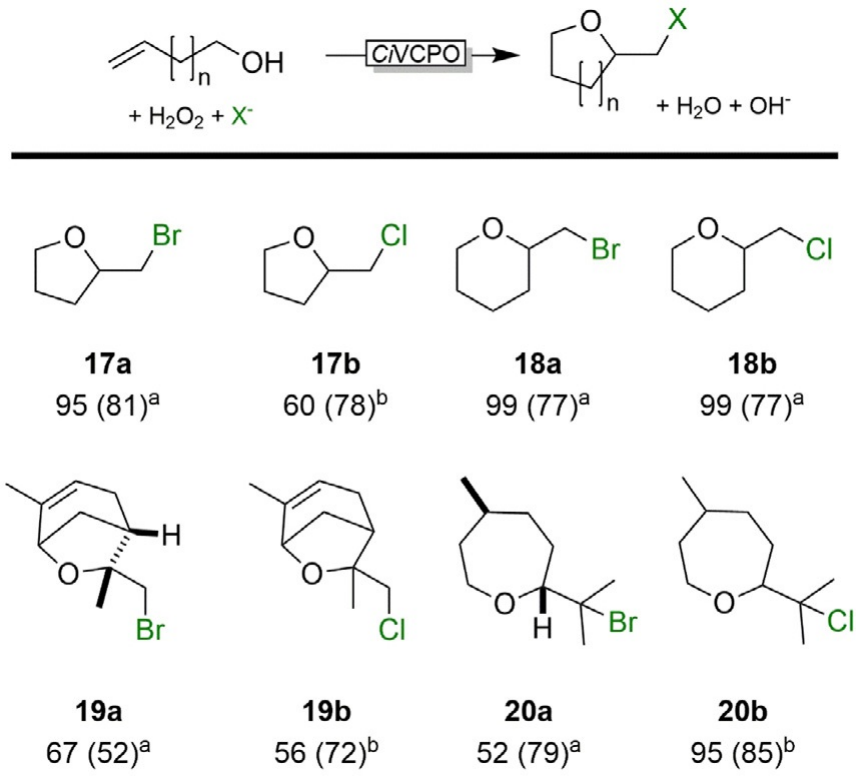
Chemoenzymatic intramolecular haloetherification. General conditions: *c*(alkenol)=40 mm; *c*(H_2_O_2_)=170 mm; *c*(KBr or KCl)=160 mm; 100 mm citrate buffer (pH 5); *c*(*Ci*VCPO)=100 nm; *T*=25 °C, *t*=24 h. [a] ^1^H NMR conversions are shown (selectivity); [b] GC conversion (selectivity).

Also, the relative configuration of compound **20 a** was determined based on NOE correlations. Based on the structure of the starting material (+)‐citronellol and of the methyl group at position 3 (CH_3_‐3), the NOE correlation of CH_3_‐5 and H‐2 elucidated the β orientation of Me‐2 and H‐2 (dH 1.13; Figures S30 and S31).

Preparative scale reactions of some selected alkenols were performed at 10 mmol scale. For example (−)‐carveol and (+)‐citronellol were converted almost quantitatively, albeit at lower selectivity than shown in Scheme 4. After 24 h, the desired products were isolated in 60 and 50 % yield, respectively.

In the current contribution, we have expanded the scope of *Ci*VCPO as a biocatalyst for organic synthesis. A semiquantitative comparison^17^ of the proposed chemoenzymatic halolactonization and haloetherification reaction with established protocols2b demonstrates its potential environmental benefits (Table 3). The mass intensities of the chemical and chemoenzymatic reactions are comparable. However, the quality of the reagents and waste products differs significantly. In the case of chemical synthesis, methylene chloride as solvent is questionable, especially compared to simple citric acid buffer. Furthermore, stoichiometric amounts of succinimide, the recycling of which necessitates further down‐stream processing (DSP) steps, is formed as a byproduct in the chemical process, whereas the chemoenzymatic process yields water (and unreacted bromide) as byproduct. Finally, the catalyst consumption of both processes also differs significantly.

**Table 3:** Semiquantitative comparison of the mass intensity of the chemical and the chemoenzymatic bromolactonization reaction. 
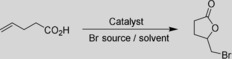

	Chemical process		Chemoenzymatic process	
	[g g^−1^_product_]		[g g^−1^_product_]	
Solvent	CH_2_Cl_2_	29.9	H_2_O	35.2
			citrate	0.67
				
Reagent	NBS	1	H_2_O_2_/KBr	0.12/0.67
				
Catalyst	mol. sieve	0.05	*Ci*VCPO	0.00016

Following the established method, the present procedure entailed extraction of the products with dichloromethane, which obviously is questionable from an environmental point‐of‐view. Therefore, future efforts will concentrate on the substitution of CH_2_Cl_2_ with more acceptable alternatives, such as ethyl acetate.^18^ A particular focus will lie on the intensification of the reaction, that is, increasing the substrate loading (and consequently also the product concentration). This will reduce the relatively large E‐factor contribution of the solvent.

Overall, we are convinced that the proposed chemoenzymatic method for halocyclization represents a promising alternative to established chemical procedures. Further upscaling and characterization of the reaction is currently ongoing in our laboratory.

## Experimental Section

A detailed description of the biocatalyst preparation and purification as well as a complete description of the experimental and analytical procedures can be found in the Supporting information.

### Halocyclization of γ,δ‐unsaturated carboxylic acids and alcohols

The halocyclization reactions were performed by using 1 mL glass vials containing 40 mm unsaturated acids, and/or alcohols in 0.1 m citrate buffer (pH 5) with 160 mm KBr and 100 nm *Ci*VCPO. Reactions were started by the addition of 100 mm of H_2_O_2_ and stirred by a magnetic bar at 500 rpm for 24 h. The reaction mixtures were extracted with ethyl acetate (1 mL; containing 5 mm acetophenone as an internal standard), dried over anhydrous MgSO_4_, and analyzed by GC (Shimadzu; see Table S1).

### Preparative‐scale chloro‐ and bromolactonization reactions

The reaction was performed in a 100 mL Erlenmeyer flask at room temperature with stirring. The reaction medium consisted of 0.1 m citrate buffer (pH 5, final volume of 50 mL) with 160 mm of KBr or KCl, 4‐pentenoic acid or 2‐methyl‐4‐pentenoic acid (10 mmol), and 100 nm *Ci*VCPO. The reaction was started by the addition of 100 mm of H_2_O_2_. After 24 h the reaction mixture was acidified, extracted with dichloromethane (3×100 mL), and dried over anhydrous Na_2_SO_4_. The combined organic layers were concentrated under reduced pressure. The chloro‐ and bromolactone products were purified by flash column chromatography on silica gel (EtOAc/hexanes, 1:2 *v*/*v*); 0.914, 1.4, and 1.15 g of chloro‐ and bromolactone products were isolated with 70, 80, and 60 % yield, respectively, as well as hydroxylactone in 30 % yield in the case of bromolactonization of 2‐methyl‐4‐pentenoic acid and analyzed by NMR spectroscopy.

### Preparative‐scale synthesis of 7‐(bromomethyl)‐4,7‐dimethyl‐6‐oxabicyclo[3.2.1]oct‐3‐ene (19 a)

The reaction was performed in a 100 mL Erlenmeyer flask at room temperature with stirring. The reaction medium consisted of 0.1 m citrate buffer (pH 5, final volume of 50 mL) with 160 mm of KBr, 10 mmol carveol and 100 nm *Ci*VCPO. The reaction was started by the addition of 100 mm of H_2_O_2_. After 24 h the reaction mixture was extracted by ethyl acetate (3×100 mL), dried over anhydrous Na_2_SO_4_. The combined organic layers were concentrated under reduced pressure. The products was purified by flash column chromatography on (silica gel, EtOAc/hexanes, 1:2); 1.38 g of 7‐(bromomethyl)‐4,7‐dimethyl‐6‐oxabicyclo[3.2.1]oct‐3‐ene (**19 a**) was isolated with 60 % yield and analyzed by NMR spectroscopy.

### Preparative‐scale of 2‐(2‐bromopropan‐2‐yl)‐5‐methyloxepane (20 a)

The reaction was performed in a 10 mL Erlenmeyer flask at room temperature with stirring. The reaction medium consisted of 0.1 m citrate buffer (pH 5, final volume of 50 mL) with 160 mm of KBr, 10 mmol (+)‐β‐citronellol and 100 nm *Ci*VCPO. The reaction was started by the addition of 100 mm of H_2_O_2_. After 24 h the reaction mixture was extracted by ethyl acetate (3×100 mL), dried over anhydrous Na_2_SO_4_. The combined organic layers were concentrated under reduced pressure. The products was purified by flash column chromatography on (silica gel, EtOAc/hexanes, 1:2); 117 mg of 2‐(2‐bromopropan‐2‐yl)‐5‐methyloxepane (**20 a**) was isolated with 50 % yield and analyzed by NMR spectroscopy.

## Conflict of interest

*The authors declare no conflict of interest*.

## Supporting information

As a service to our authors and readers, this journal provides supporting information supplied by the authors. Such materials are peer reviewed and may be re‐organized for online delivery, but are not copy‐edited or typeset. Technical support issues arising from supporting information (other than missing files) should be addressed to the authors.

Supplementary
