# Supplementary material for: Chemoenzymatic Halocyclization of γ,δ‐Unsaturated Carboxylic Acids and Alcohols
Source: ChemSusChem. 2019 Oct 22;13(1):97–101. doi: 10.1002/cssc.201902240 (PMC6973245; doi:10.1002/cssc.201902240)
Supplement: Supplementary file 1 — Supplementary [file CSSC-13-97-s001.pdf]

CHEMISTRY & SUSTAINABILITY
CHEMSUSCHEM
ENERGY & MATERIALS

Supporting Information

Chemoenzymatic Halocyclization of γ,δ-Unsaturated Carboxylic Acids and Alcohols

Sabry H. H. Younes,[a, b] Florian Tieves,[a] Dongming Lan,[c] Yonghua Wang,[c] Philipp Süss,[d] Henrike Brundiek,[d] Ron Wever,[e] and Frank Hollmann*[a]

cssc_201902240_sm_miscellaneous_information.pdf

Chemoenzymatic halocyclisation of γ,δ-unsaturated carboxylic acids and alcohols

Sabry H. H. Younes,[a,b] Florian Tieves,[a] Dongming Lan,[c] Yonghua Wang,[c] Philipp Süss,[d] Henrike Brundiek,[d] Ron Wever[e] and Frank Hollmann[a]*

Supporting information:

[a] Dr. S.H.H. Younes, Dr. F. Tieves, Prof. Dr. F. Hollmann
Department of Biotechnology
Delft University of Technology
Van der Maasweg 9, 2629 HZ Delft, The Netherlands
E-mail: f.hollmann@tudelft.nl

[b] Dr. S.H.H. Younes
Department of Chemistry, Faculty of Sciences,
Sohag University, Sohag 82524, Egypt

[c] Dr. D. Lan, Prof. Dr. Y. Wang
School of Food Science and Engineering, Overseas Expertise Introduction Center for Discipline Innovation of Food Nutrition and Human Health (111 Center)
South China University of Technology Guangzhou 510640, P.R. China

[d] Dr. P. Süss, Dr. H. Brundiek
Enzymicals AG, Walther-Rathenau-Str. 49a, 17489 Greifswald, Germany

[e] Prof. Dr. R. Wever
University of Amsterdam, Van't Hoff Institute for Molecular Sciences, Amsterdam, The Netherlands

Table of contents

1. General information ……………………………………………………………………………………...S3
2. Preparation of vanadium chloroperoxidase from Curvularia inaequali (CiVCPO)……..S3
3. Procedure for halocyclisation of δ,γ-unsaturated carboxylic acids and alcohols……...S4
4. Semi-preparative scale of halocyclisation reaction…………………………………………………..S4
5. Preparative-scale
   5.1. Preparative-scale of chloro- and bromolactonisationreaction……………………......S5
   5.2. Preparative-scale of 7-(bromomethyl)-4,7-dimethyl-6-oxabicyclo[3.2.1]oct-3-ene (19a)……………………………………………………………………………………………………S5
   5.3. Preparative-scale of 2-(2-bromopropan-2-yl)-5-methyloxepane (20a)……….S5
6. General Procedure for bromolactone Kinetic Resolution (KR) ……………..……………..…..S5
   6.1. Porcine pancreas lipase (PPL) ………………………………………………………..….S5
   6.2. Lipase MAS1 ……………………….…………………………………………………..….S5
7. Analytical methods ……………………………..……………………………………………….….…...S5
   7.1. GC analyses………………………..………….………………………………………………..…...S6
   7.2. GC analytics …………………….………………….……………………………..…………………..S8
   7.3. NMR-Analysis………………………….……………..………………………………..……………….S9
   7.4. NMR Spectra……………………………………………..………………………………..…………...S12
   7.5. GC Chromatograms………………………………..……………………………………..………...S27
   7.6. Kinetic Resolution (KR) Chromatograms…………………………………………………..S39

1. General information

All commercial reagents and solvents were purchased from Sigma-Aldrich with the highest purity available and used as received. Reactions were carried out under inert atmosphere of dry argon. Flash chromatography was carried out using Acros silica gel (0.035–0.070 mm, and ca. 6 nm pore diameter). NMR spectra were recorded on a Varian 400 (400 MHz) spectrometer in $CDCl_3$. Chemical shifts are given in ppm with respect to tetramethylsilane. Coupling constants are reported as *J*-values in Hz.

2. Preparation of vanadium chloroperoxidase from *Curvularia inaequalis* (*CiVCPO*)

For heterologous expression and purification of *CiVCPO* a slightly modified literature procedure was used: A 2 L culture of Escherichia coli transformant [E. coli TOP10 (Invitrogen) with the construct pBAD-*CiVCPO*] was grown at 37 °C in Lysogeny broth medium supplemented with 100 µg/mL ampicillin to an OD 600 nm of 0.6-0.8. Protein expression was induced after cooling the fermentation broth to 20 °C and addition of 0.02 % L arabinose, followed by another 72 hours of incubation. The expression of *CiVCPO* in E. coli yielded an enzyme content of 15 mg·L-1 culture. Cells were harvested by centrifugation at 8000 rpm for 10 min at 4 °C. The cells were re-suspended to 1 g mL-1 in 50 mM Tris/H_2SO_4, pH 8.1 fortified with protease inhibitors, lysozyme (2 mg mL-1) and DNaseI. Cells were lysed using a Cell disruptor and debris was removed by centrifugation at 15000 rpm for 1 h at 4 °C. After centrifugation an equal volume of isopropyl alcohol was added to the supernatant to precipitate nucleic acids and unstable proteins. After centrifugation (30 min at 15000 rpm), the clear supernatant was applied to a DEAE Sephacel column (Amersham Pharmacia Biotech) (5mL min-1) equilibrated with 50 mM Tris/H_2SO_4 pH 8.1. After washing of the column with 2 volumes of 50 mM Tris/H_2SO_4, pH 8.1, and 2 volumes of 0.1 M NaCl in 50 mM Tris/H_2SO_4, pH 8.1, the enzyme was eluted with 1 M NaCl in 50 mM Tris/HCl, pH 8.1. Finally, the pure apoenzyme was dialyzed against 100 µM orthovanadate in 50 mM Tris H_2SO_4, pH 8.1 to obtain the reconstituted holoenzyme. As illustrated below, SDS-PAGE monitoring of the purification process showed that the whole soluble fraction from the E. coli cultures (lane 4) was considerably enriched in the *CiVCPO* (67.5 kDa) band, incubation and centrifugation with isopropanol (lane 3) partially removed undesired proteins and finally after DEAE chromatography (lanes 5 and 6) protein was ≥ 90 % pure. Protein concentration was estimated by the BSA assay and *CiVCPO* activity was determined to be 120 U mg^{-1}. One unit of the enzyme activity was defined as the amount of the enzyme that catalyzes the bromination of 1 µmol monochlorodimedon per min at pH 5 and 30 °C (using a saturating concentration of bromide (5 mM) in 0.1 M citrate (pH 5) after the addition of 10 mM of H_2O_2).
Purification steps of *CiVCPO* (lane 1 crude extract with isopropanol; lane 2 crude extract with isopropanol after centrifugation; lane 3 standard (99 kDa, 66 kDa, 45 kDa and 30 kDa); lane 4

crude extract, lanes 5-6 purified enzyme).

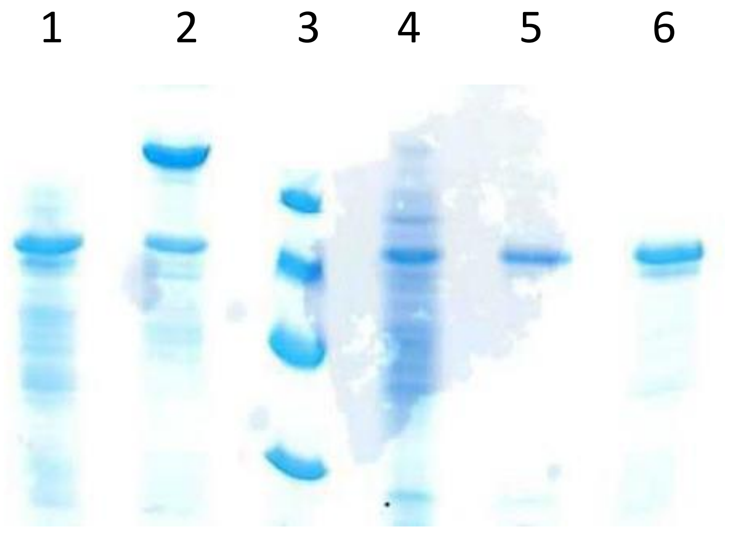

3. Procedure for halocyclisation of δ,γ-unsaturated carboxylic acids and alcohols

The halocyclisation reactions were performed by using 1 mL glass vial containing 40 mM of unsaturated acids, and/or alcohols in 0.1 M citrate buffer (pH 5) with 160 mM of KBr, followed by 100 nM of vanadium-dependent chloroperoxidase from the fungus *Curvularia inaequalis* (*CiVCPO*). 100 mM of H_2O_2 was added in one portion and continuously stirred by a magnetic bar at 500 rpm for 24 h.

GC analytics
The reaction mixtures were extracted with ethyl acetate 1 mL (containing 5 mM of acetophenone as an internal standard), dried over anhydrous $MgSO_4$ and analysed by GC (SHIMADZU, see Table S1).

4. Semi-preparative scale of halocyclisation reaction

To 4 mL glass vial containing 40 mM of unsaturated acids, and/or alcohols in 0.1 M citrate buffer pH 5 (final volume of 2.6 mL) with 160 mM of KBr, followed by 100 nM of vanadium-dependent chloroperoxidase from the fungus *Curvularia inaequalis* (*CiVCPO*). 100 mM of H_2O_2 was added in one portion and continuously stirred by a magnetic bar at 500 rpm for 24 h. The reaction mixture was acidified, extracted by dichloromethane (3x5 mL), The combined organic layers were reduced in *vacuo*, dried over anhydrous Na_2SO_4 and analysed by NMR.

5. Preparative-scale

5.1. Preparative-scale of chloro- and bromolactonisation reaction

The reaction was performed in a 100 mL Erlenmeyer flask at room temperature for 24 h with magnetic stirring, contained 0.1 M citrate buffer (pH 5, final volume of 50 mL) with 160 mM of KBr or KCl, 10 mmol 4-pentenoic acid and/or 2-methyl-4-pentenoic acid, followed by the addition of 100 nM of vanadium-dependent chloroperoxidase from the fungus *Curvularia inaequalis* (*CiVCPO*) and 100 mM of H_2O_2 was added one portion under adJusted concentrations of reagents and buffer strength. The reaction mixtures were acidified, extracted by dichloromethane (3x100 mL), dried over anhydrous Na_2SO_4. The combined organic layers were reduced in *vacuo*. The chloro- and bromolactone products were isolated by flash column chromatography on (silica gel, EtOAc:Hexanes, 1:2); 0.914, 1.4 and 1.15 g of the desired chloro- and bromolactone products were isolated corresponding to 70, 80 and 60% isolated yield, as well as 0.58 g (30%) of hydroxylactone in case of bromolactonisation of 2-methyl-4-pentenoic acid, respectively, and analysed by NMR.

5.2. Preparative-scale of 7-(bromomethyl)-4,7-dimethyl-6-oxabicyclo[3.2.1]oct-3-ene (19a).

The reaction was performed in a 100 mL Erlenmeyer flask at room temperature with stirring. The reaction medium consisted of 0.1 M citrate buffer (pH 5, final volume of 50 mL) with 160 mM of KBr, 10 mmol carveol and 100 nM *Ci*VCPO. The reaction was started by the addition of 100 mM of H_2O_2. After 24h the reaction mixture was extracted by ethyl acetate (3x100 mL), dried over anhydrous Na_2SO_4. The combined organic layers were reduced *in vacuo*. The products was purified by flash column chromatography on (silica gel, EtOAc:Hexanes, 1:2); 1.38 g of 7-(bromomethyl)-4,7-dimethyl-6-oxabicyclo[3.2.1]oct-3-ene (**19a**) was obtained with 60% isolated yield, and analysed by NMR.

5.3. Preparative-scale of 2-(2-bromopropan-2-yl)-5-methyloxepane (20a)

The reaction was performed in a 10 mL Erlenmeyer flask at room temperature with stirring. The reaction medium consisted of 0.1 M citrate buffer (pH 5, final volume of 50 mL) with 160 mM of KBr, 10 mmol (+)-β-citronellol and 100 nM *Ci*VCPO. The reaction was started by the addition of 100 mM of H_2O_2. After 24h the reaction mixture was extracted by ethyl acetate (3x100 mL), dried over anhydrous Na_2SO_4. The combined organic layers were reduced *in vacuo*. The products was purified by flash column chromatography on (silica gel, EtOAc:Hexanes, 1:2); 117 mg of 2-(2-bromopropan-2-yl)-5-methyloxepane (**20a**) was obtained with 50% isolated yield, and analysed by NMR.

6. General procedure for bromolactone Kinetic Resolution (KR)

6.1. Porcine pancreas lipase (PPL)

The reaction was performed by using 1 mL glass vial containing 10 mM of bromolactone was suspended in 100 mM phosphate buffer (KPi, pH 7.5); porcine pancreas lipase (PPL) (10 mg) was added and continuously stirred 500 rpm at 30 ºC for 24 h. The reaction was monitored by chiral GC.

6.2. Lipase MAS1

The reaction was performed by using 1 mL glass vial containing 10 mM of bromolactone was suspended in 100 mM phosphate buffer (KPi, pH 8.0); MAS1 lipase (1 mg) was added and continuously stirred 500 rpm at 40 ºC for 3h. The reaction was monitored by chiral GC.

The reaction mixtures were extracted with ethyl acetate (containing 5 mM of acetophenone as an internal standard), dried over anhydrous $MgSO_4$ and analysed by GC (SHIMADZU).

7. Analytical methods

7.1. GC analyses

The progress of the reaction was followed by GC and all product concentrations were calculated based on calibration curve equations using 5 mM acetophenone as an internal standard. GC analyses were carried out on a Shimadzu GC-2010 gas chromatograph equipped with an FID on the column CP sil 5CB (50 m × 0.53 mm × 1.0 µm), FID, N2 is the carrier gas and Hydrodex β-TBDM (50 m × 0.25 mm × 0.25 µm), FID, He is the carrier gas; The calibration curves using 5 mM acetophenone as an internal standard were linear in the range of product detection ($R^2 > 0.999$). The following acquisition parameters were used:

Method A. Oven program: Oven program: 80 °C for 5 min, 10 °C/min to 105 °C for 1 min, 10 °C/min to 115 °C for 1.5 min, 10 °C/min to 160 °C for 1.5 min, 30 °C/min to 325 °C for 1 min; total flow rate: 20 mL/min; injector temperature: 220 °C; detector temperature: 350 °C; splitless injection.

Method B. Oven program: 80 °C for 5 min, 30 °C/min to 140 °C for 0.7 min, 30 °C/min to 325 °C for 0.7 min; total flow rate: 20 mL/min; injector temperature: 220 °C; detector temperature: 350 °C; splitless injection.

Method C. Oven program: 80 °C for 5 min, 10 °C/min to 105 °C for 1 min, 10 °C/min to 115 °C for 1.5 min, 10 °C/min to 160 °C for 1.5 min, 30 °C/min to 325 °C for 1 min; total flow rate: 20 mL/min; injector temperature: 220 °C; detector temperature: 350 °C; splitless injection.

Method D. Oven program: 120 °C for 2.5 min, 20 °C/min to 145 °C for 1 min, 20 °C/min to 185 °C for 4 min, 25 °C/min to 225 °C for 1.5 min; total flow rate: 20 mL/min; injector temperature: 250 °C; detector temperature: 275 °C; splitless injection.

7.2. G C analytics

Table S1. Details for GC analysis

Substrate	t_R (min)	Product	t_R (min)		Column program
			X=Br	X= Cl	
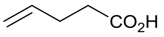	2.3	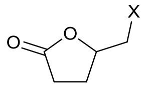	5.90	7.60	A
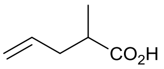	2.8	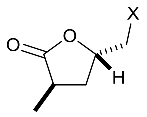	6.56	7.88	A
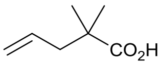	3.2	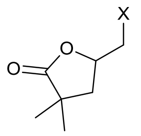	6.50	7.85	A
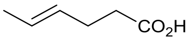	3.2	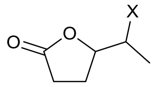	6.36	7.76	A
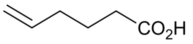	2.9	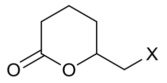	8.10	8.90	A
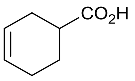	5.2	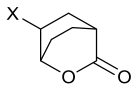	7.99	8.82	A
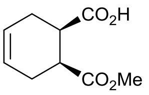	3.36	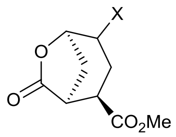	10.54	11.08	A
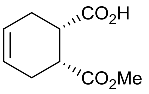	3.36	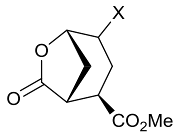	10.54	11.08	A
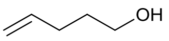	1.78	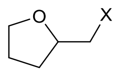	10.20	12.46	B
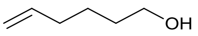	2.72	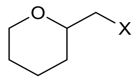	6.70	6.72	B
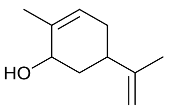	11.50	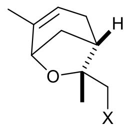	18.44	18.38	C
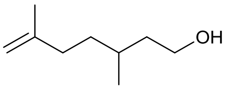	11.68	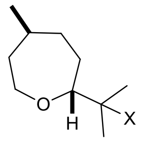	18.38	17.44	C

7.3. NMR-Analysis

5-(Bromomethyl)dihydrofuran-2(3H)-one (9a):

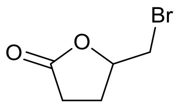

^{1}H NMR (400 MHz, Chloroform-*d*) δ 4.78-4.67 (m, 1H), 3.56 (dd, *J* = 10.8, 4.3 Hz, 1H), 3.52 (dd, *J* = 10.8, 6.0 Hz, 1H), 2.70-2.49 (m, 3H), 2.72-2.50 (m, 3H), 2.18-2.10 (m, 1H). ^{13}C NMR (101 MHz, Chloroform-d) δ 176.29, 78.55, 28.95, 28.25, 7.48.

5-(Chloromethyl)dihydrofuran-2(3H)-one(9b):

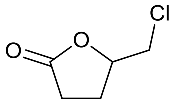

^{1}H NMR (400 MHz, Chloroform-*d*) 4.64-4.61 (m, 1H), 3.91 (dd, *J* = 12.5, 2.9 Hz, 1H), 3.66 (dd, *J* = 12.4, 4.7 Hz, 1H), 2.69-2.51 (m, 3H), 2.16-2.10 (m, 1H).

5-(Bromomethyl)-3-methyldihydrofuran-2(3H)-one (10a):

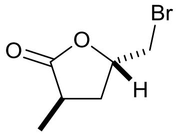

^{1}H NMR (400 MHz, Chloroform-*d*) δ 4.62-4.53 (m, 1H), 3.83 (dd, *J* = 12.5, 3.0 Hz, 1H), 3.61 (dd, *J* = 12.5, 4.6 Hz, 1H), 2.82-2.78 (m, 1H), 2.38-2.31 (m, 1H), 1.97-1.91 (m, 1H), 1.24 (d, *J* = 7.0 Hz, 3H).

5-(Hydroxymethyl)-3-methyldihydrofuran-2(3H)-one :

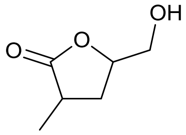

^{1}H NMR (400 MHz, Chloroform-d) δ 4.51-4.48 (m, 1H), 3.88 (dd, *J* = 12.7, 2.8 Hz, 1H), 3.60 (dd, *J* = 12.7, 5.0 Hz, 1H), 2.75-2.68 (m, 1H), 2.40-2.33 (m, 1H), 1.84-1.72 (m, 1H), 1.26 (d, 3H).

5-(Chloromethyl)-3-methyldihydrofuran-2(3H)-one (10b):

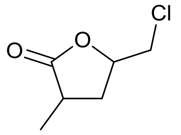

^{1}H NMR (400 MHz, Chloroform-*d*) δ 4.76-4.70 (m, 1H), 3.69-3.67 (dd, *J* = 12.4, 2.9 Hz, 1H), 3.65-3.63 (dd, *J* = 12.4, 4.7 Hz, 1H), 2.61-2.53 (m, 1H), 2.46-2.34 (m, 1H), 2.10-2.02 (m, 1H), 1.29 (d, *J* = 2.7 Hz, 3H).

5-(Bromomethyl)-3,3-dimethyldihydrofuran-2(3H)-one (11a):

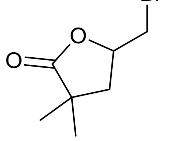

^{1}H NMR (400 MHz, Chloroform-*d*) δ 4.66-4.58 (m, 1H), 3.57 (dd, *J* = 10.7, 4.7 Hz, 1H), 3.46 (dd, *J* = 10.7, 6.5 Hz, 1H), 2.27 (dd, *J* = 12.9, 6.3 Hz, 1H), 1.92 (dd, *J* = 12.9, 9.5 Hz, 1H), 1.30 (s, 3H), 1.28 (s, 3H).

5-(Chloromethyl)-3,3-dimethyldihydrofuran-2(3H)-one (11b):

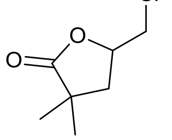

^{1}H NMR (400 MHz, Chloroform-d) δ 4.62-4.56 (m, 1H), 3.57 (dd, *J* = 10.7, 4.7 Hz, 1H), 3.44 (dd, *J* = 10.7, 6.5 Hz, 1H), 2.27 (dd, *J* = 12.9, 6.3 Hz, 1H), 1.92 (dd, *J* = 12.9, 9.5 Hz, 1H), 1.30 (s, 3H), 1.28 (s, 3H).

5-(1-Bromoethyl)dihydrofuran-2(3H)-one (12a):

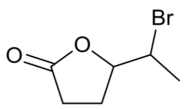

^{1}H NMR (400 MHz, Chloroform-*d*) δ δ 4.37-4.30 (m, 1H), 3.77 (q, *J* = 6.5, 5.4 Hz, 1H), 2.6-2.54 (m, 1H), 2.28-2.22 (m, 1H), 2.08-1.98 (m, 1H), 1.78 (d, 3H).

5-(1-Chloroethyl)dihydrofuran-2(3H)-one (12b):

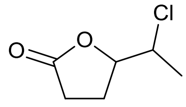

^{1}H NMR (400 MHz, Chloroform-*d*) δ 4.44 (q, *J* = 7.1 Hz, 1H), 4.33 (td, *J* = 7.3, 5.3 Hz, 1H), 2.62-2.54 (m, 2H), 2.49-2.36 (m, 1H), 2.30-2.19 (m, 1H), 1.74 (d, J = 6.7 Hz, 3H).

6-(Bromomethyl)tetrahydro-2H-pyran-2-one (13a):

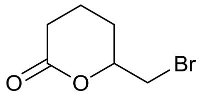

^{1}H NMR (400 MHz, Chloroform-*d*) δ 4.53-4.47 (m, 1H), 3.53 (dd, *J* = 10.7, 4.5 Hz, 1H), 3.47 (dd, *J* = 10.8, 6.3 Hz, 1H), 2.44-2.39 (m, 2H), 2.00-1.93 (m, 1H), 1.88-1.82 (m, 2H), 1.76-1.68 (m, 1H).

6-(Chloromethyl)tetrahydro-2H-pyran-2-one (13b):

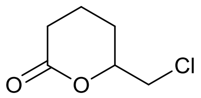

^{1}H NMR (400 MHz, Chloroform-*d*) δ 4.56-4.48 (m, 1H), 3.64 (dd, *J* = 11.1, 7.1 Hz, 1H), 3.48 (dd, *J*= 11.1, 7.1 Hz, 1H), 2.44-2.40 (t, J = 7.2 Hz, 3H), 1.87-1.85 (m, 1H), 1.78-1.73 (m, 2H), 1.62-1.58 (m, 1H).

4-Bromo-6-oxabicyclo[3.2.1]octan-7-one (14a):

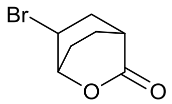

^{1}H NMR (400 MHz, Chloroform-d) δ 4.74 (dd, *J* = 13.0, 6.1 Hz, 1H), 3.80-3.72 (m, 1H), 2.64 (q, *J* = 6.0, 5.1 Hz, 1H), 2.33-2.21 (m, 2H), 1.98-1.84 (m, 3H), 1.60-1.45 (m, 2H).

4-Chloro-6-oxabicyclo[3.2.1]octan-7-one (14b):

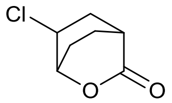

^{1}H NMR (400 MHz, Chloroform-d) δ 4.75-4.70 (m, 1H), 3.79-3.67 (m, 1H), 2.66-2.63 (m, 1H), 2.51-2.42 (m, 2H), 1.95-1.84 (m, 2H), 1.59-1.52 (m, 2H).

Methyl (1R,2S)-4-bromo-7-oxo-6-oxabicyclo[3.2.1]octane-2-carboxylate (15a):

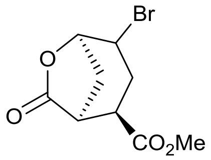

^{1}H NMR (400 MHz, Chloroform-d) δ 4.84-4.77 (m, 1H), 4.44 (q, *J* = 5.3, 4.1 Hz, 1H), 3.74 (s, 3H), 2.97-2.94 (m, 1H), 2.61-2.53 (m, 1H), 2.49-2.38 (m, 1H).

Methyl (1R,2S)-4-chloro-7-oxo-6-oxabicyclo[3.2.1]octane-2-carboxylate (15b):

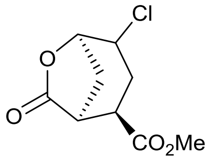

^{1}H NMR (400 MHz, Chloroform-*d*) δ 4.75 (dd, *J* = 5.7, 4.5 Hz, 1H), 4.39 (t, *J* = 4.7 Hz, 1H), 3.74 (s, 3H), 3.11 (d, *J* = 5.3 Hz, 1H), 2.97 (ddd, *J* = 12.3, 5.8, 1.9 Hz, 1H), 2.51-2.32 (m, 4H).

Methyl (1R,2R)-4-bromo-7-oxo-6-oxabicyclo[3.2.1]octane-2-carboxylate (16a):

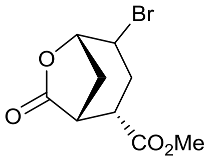

^{1}H NMR (400 MHz, Chloroform-*d*) δ 4.80-4.70 (q, *J* = 4.8 Hz, 1H), 4.39 (t, *J* = 4.8 Hz, 1H), 3.74 (s, 3H), 3.09 (dd, *J* = 20.4, 5.7 Hz, 1H), 3.01-2.91 (m, 1H), 2.70-2.64 (m, 1H), 2.47- 2.34 (m, 2H), 2.16-2.00 (m, 1H).

Methyl (1R,2R)-4-chloro-7-oxo-6-oxabicyclo[3.2.1]octane-2-carboxylate (16b):

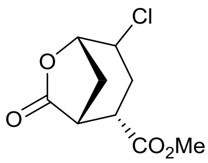

^{1}H NMR (400 MHz, Chloroform-*d*) δ 4.78-4.74 (m, 1H), 4.39 (t, *J* = 4.7 Hz, 1H), 3.74 (s, 4H), 3.11 (d, *J* = 5.3 Hz, 1H), 2.97 (ddd, *J* = 12.3, 5.8, 1.9 Hz, 2H), 2.46-2.37 (m, 3H), 1.76 (d, *J* = 12.4 Hz, 1H).

2-(Bromomethyl)tetrahydrofuran (17a):

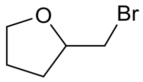

^{1}H NMR (400 MHz, Chloroform-*d*) δ 3.89-3.82 (m, 1H), 3.74-3.68 (m, 2H), 3.53 (dd, *J* = 10.3, 3.7 Hz, 1H), 3.41 (dd, *J* = 10.3, 7.1 Hz, 1H), 1.76-1.60 (m, 4H).

2-(Bromomethyl)tetrahydro-2H-pyran (18a):

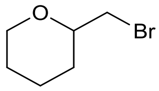

^{1}H NMR (400 MHz, Chloroform-*d*) δ 3.82-3.77 (m, 1H), 3.69-3.64 (m, 2H), 3.54 (dd, *J* = 10.3, 3.3 Hz, 1H), 3.39 (dd, *J* = 10.3, 7.1 Hz, 1H), 1.68-1.55 (m, 6H).

2-(Chloromethyl)tetrahydro-2H-pyran (18b):

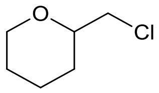

^{1}H NMR (400 MHz, Chloroform-*d*) δ 3.86-3.77 (m, 1H), 3.71-3.67 (m, 2H), 3.62 (dd, *J* = 10.3, 3.3 Hz, 1H), 3.48 (dd, *J* = 11.1, 7.1 Hz, 1H), 1.67-1.54 (m, 6H).

7-(Bromomethyl)-4,7-dimethyl-6-oxabicyclo[3.2.1]oct-3-ene (19a):

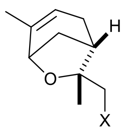

^{1}H NMR (400 MHz, Chloroform-d) δ 5.51 (dd, *J* = 35.9, 5.5 Hz, 1H), 4.54 (dt, *J* = 10.7, 5.4 Hz, 1H), 3.56 (dd, *J* = 10.8, 4.8 Hz, 1H), 3.48-3.42 (m, 1H), 2.38 (dd, *J* = 11.8, 7.2 Hz, 2H), 2.23 (s, 1H), 2.09-2.01 (m, 2H), 1.74 (s, 3H), 1.30 (s, 3H).

2-(2-Bromopropan-2-yl)-5-methyloxepane (20a):

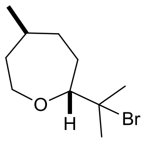

^{1}H NMR (400 MHz, Chloroform-*d*) δ δ 5.10 (t, J = 7.2 Hz, 1H), 3.73-3.66 (m, 2H), 1.68 (s, 3H), 1.60 (s, 3H), 1.57-1.51 (m, 2H), 1.40-1.31 (m, 3H), 1.23-1.11 (m, 2H), 0.91 (d, J = 6.4 Hz, 3H).

7.4. NMR Spectra

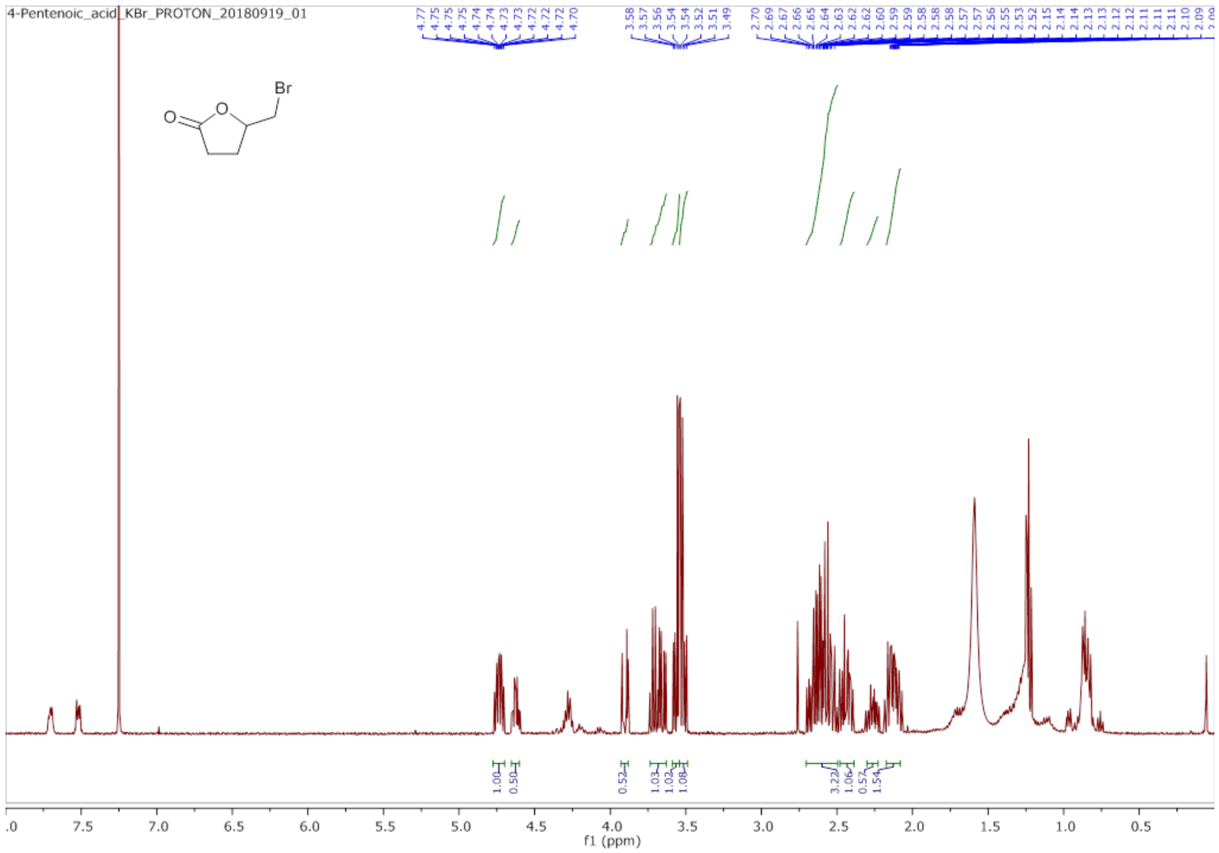

Scheme 1. ^{1}H NMR of 5-(bromomethyl)dihydrofuran-2(3H)-one **(9a)**.

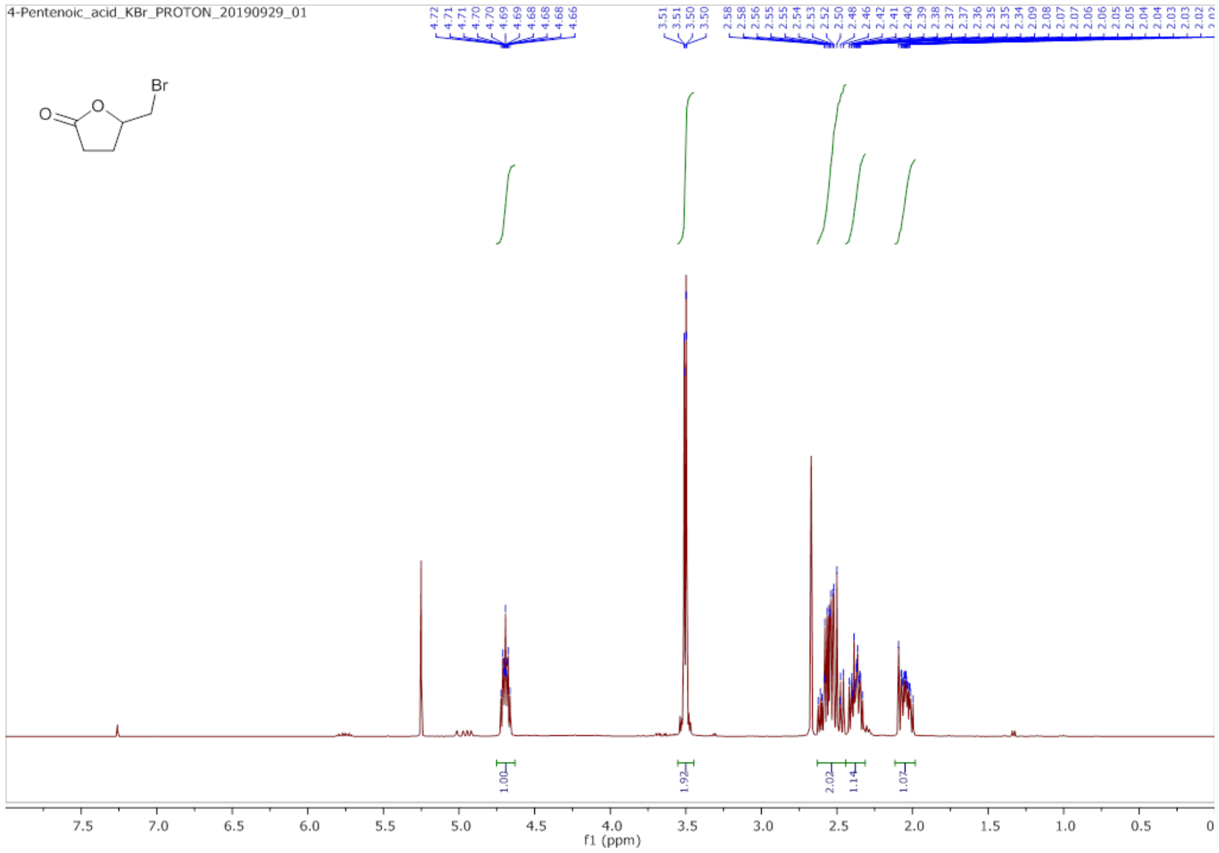

Scheme 2. ^{1}H NMR of pure 5-(bromomethyl)dihydrofuran-2(3H)-one **(9a)**.

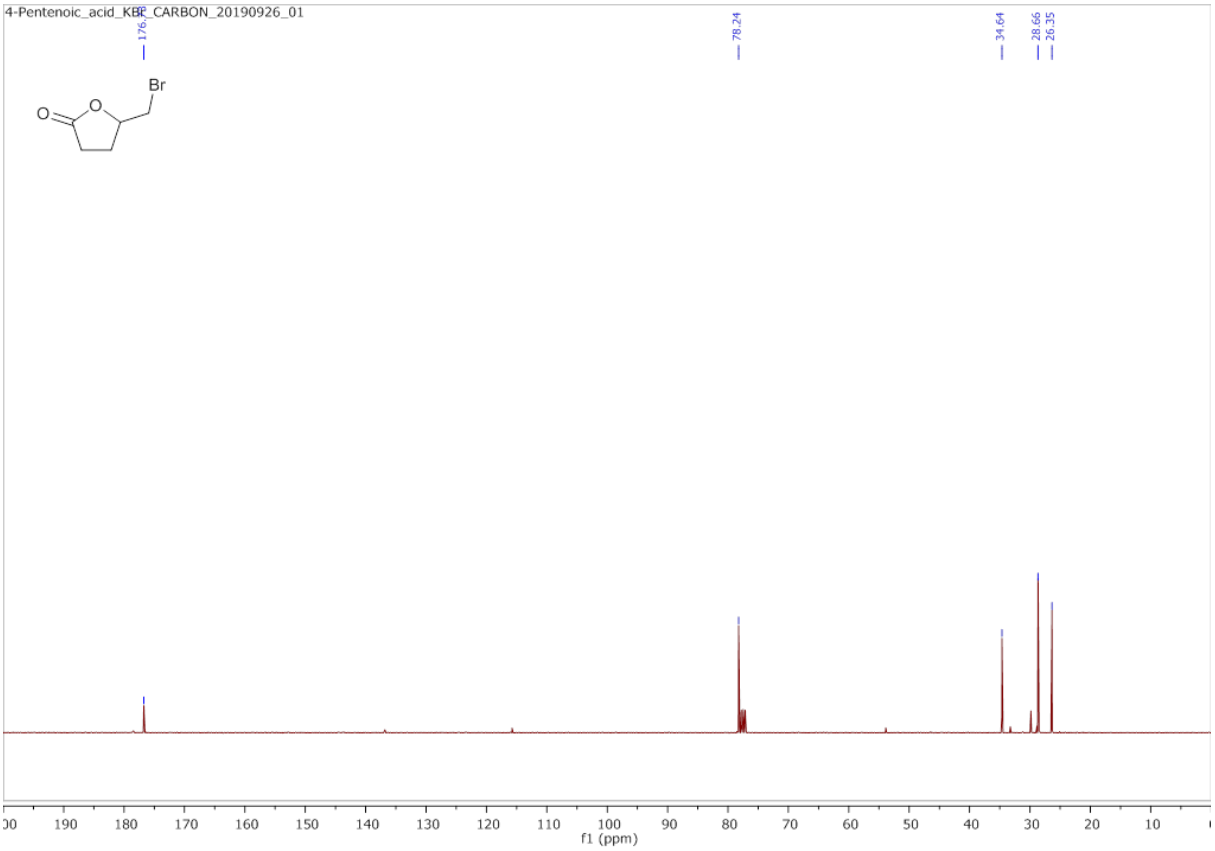

Scheme 3. ^{13}C NMR of pure 5-(bromomethyl)dihydrofuran-2(3H)-one **(9a)**.

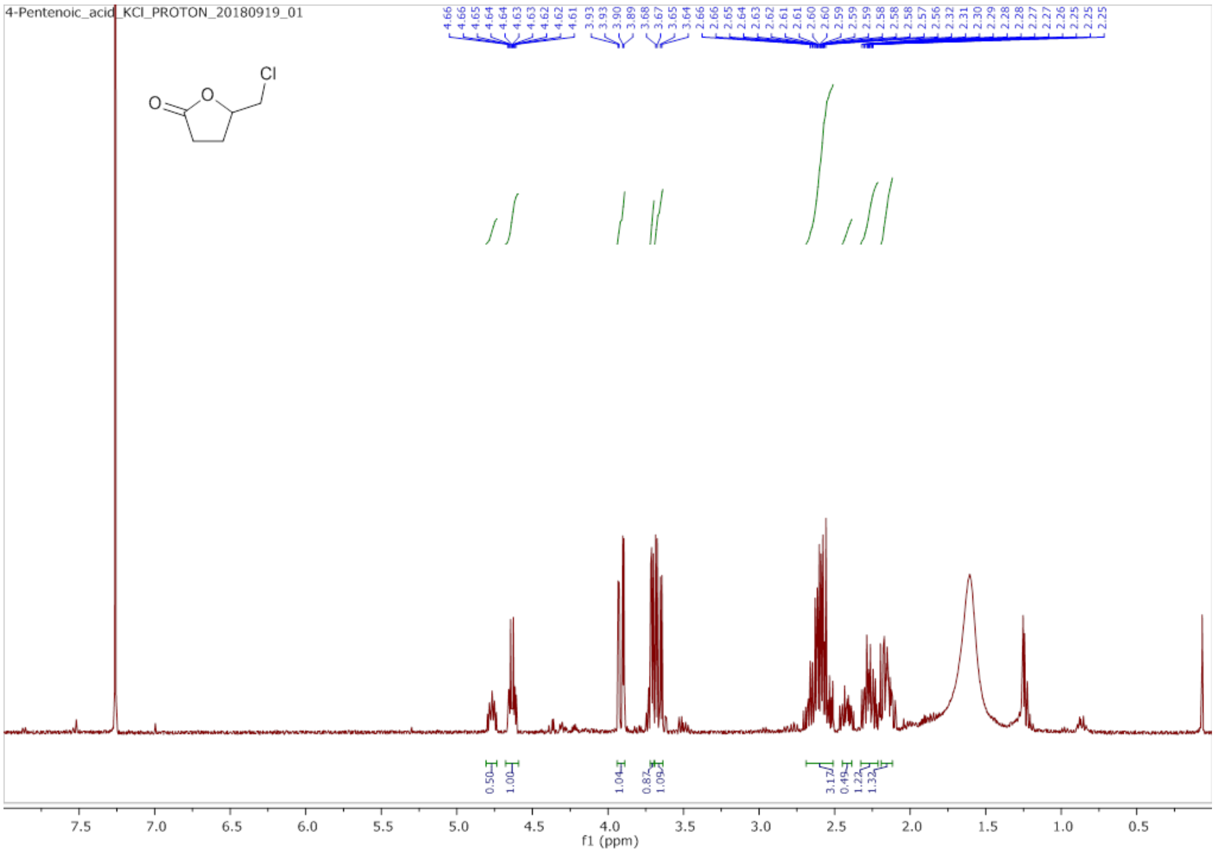

Scheme 4. ^{1}H NMR of 5-(chloromethyl)dihydrofuran-2(3H)-one **(9b)**.

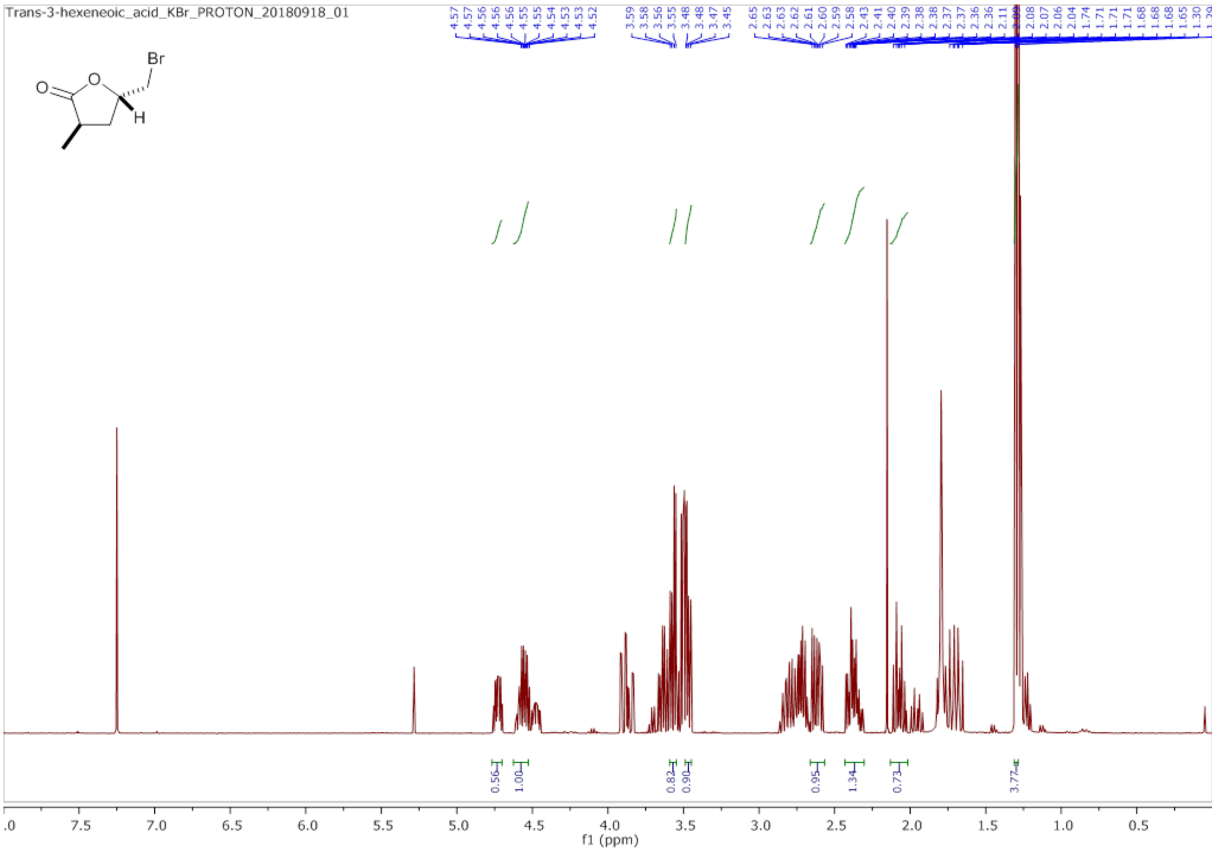

Scheme 5. ^{1}H NMR of 5-(bromomethyl)-3-methyldihydrofuran-2(3H)-one **(10a)**.

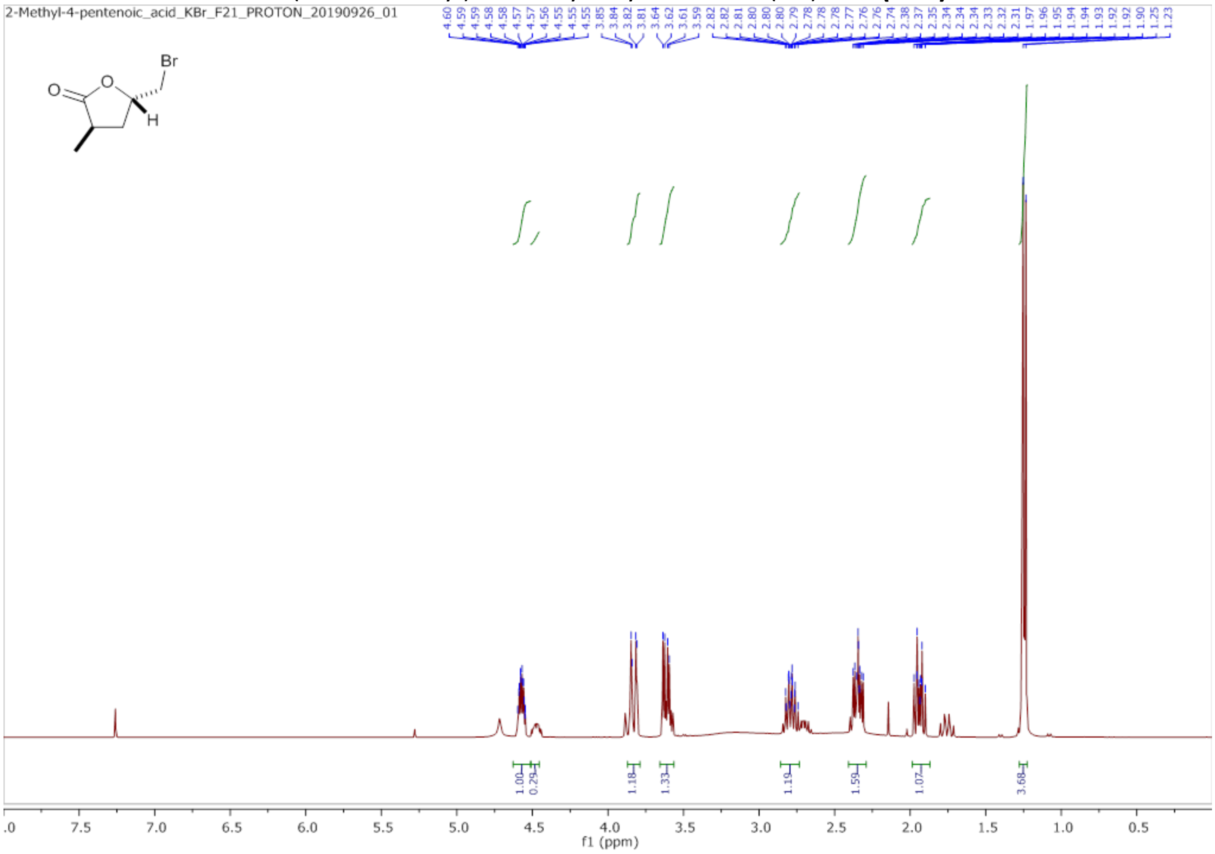

Scheme 6. ^{1}H NMR of pure 5-(bromomethyl)-3-methyldihydrofuran-2(3H)-one **(10a)**.

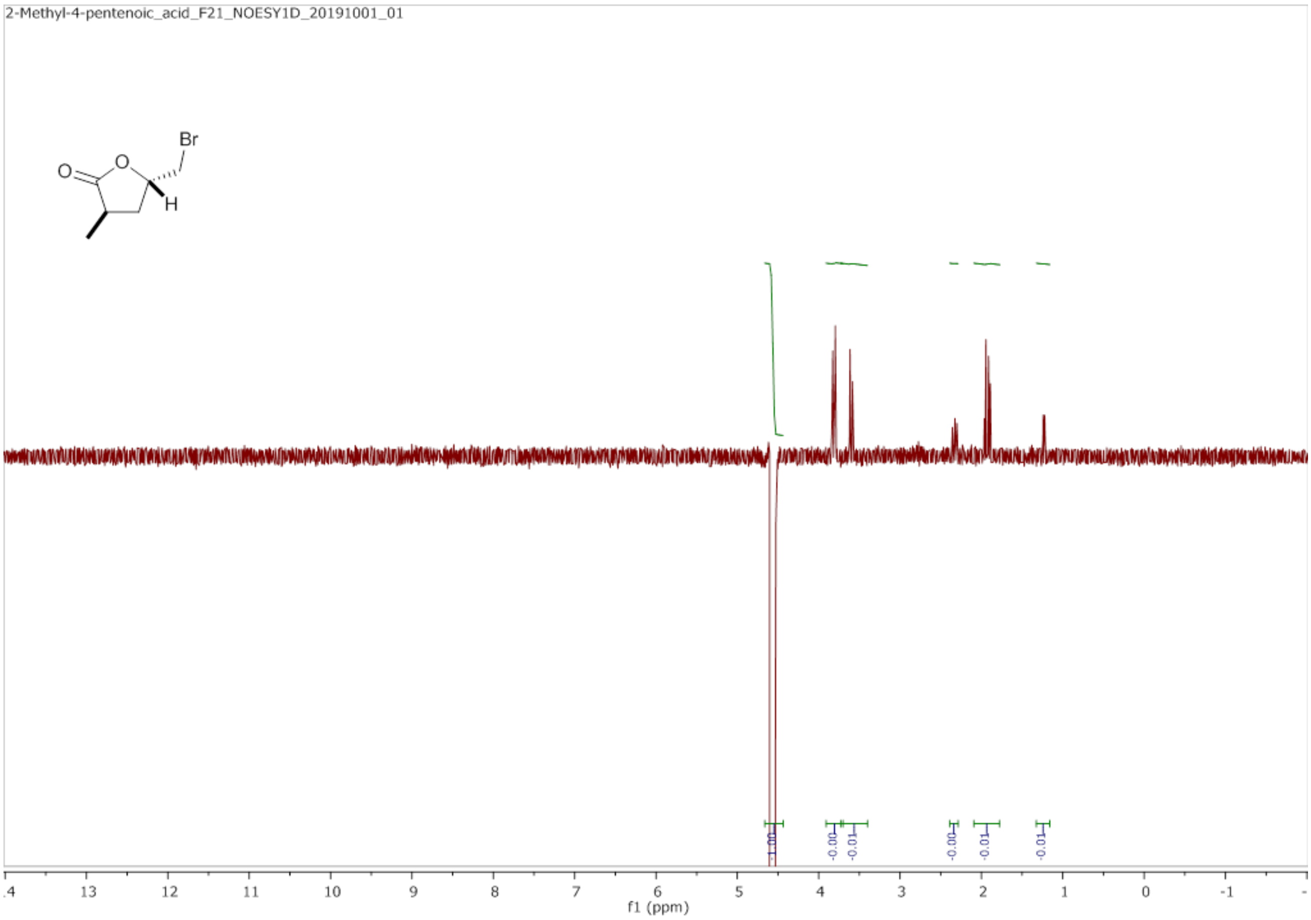

Scheme 7. NOESY1D (NOE)of 5-(bromomethyl)-3-methyldihydrofuran-2(3H)-one **(10a)**.

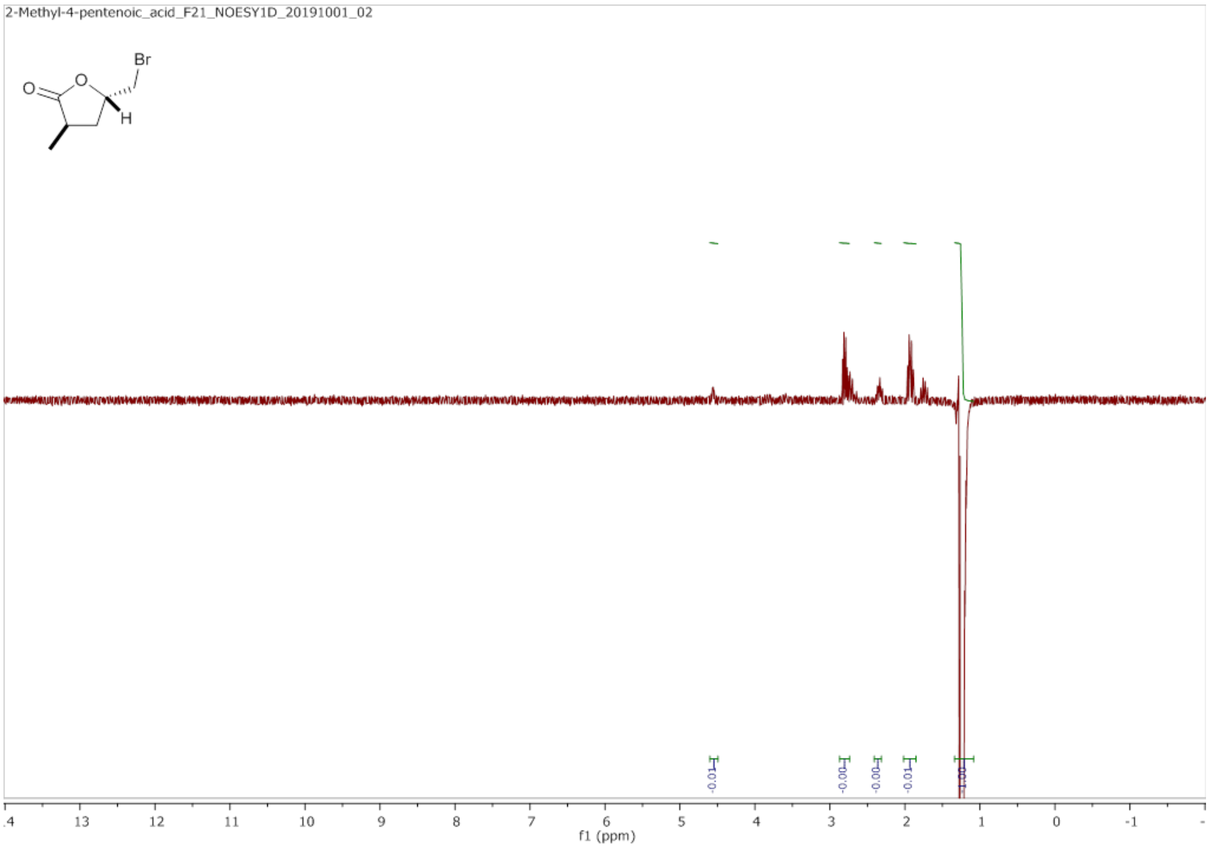

Scheme 8. NOESY1D (NOE)of 5-(bromomethyl)-3-methyldihydrofuran-2(3H)-one **(10a)**.

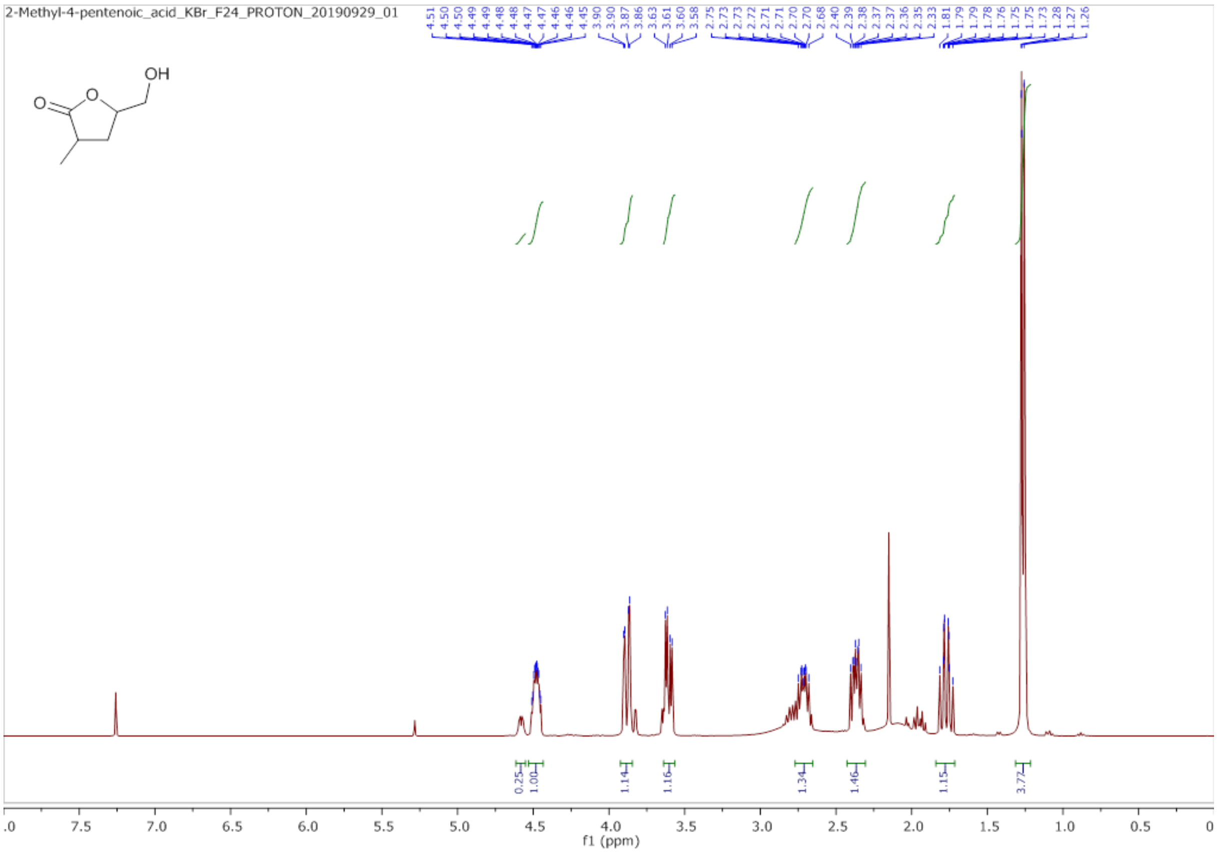

Scheme 9. ^{1}H NMR of 5-(hydroxymethyl)-3-methyldihydrofuran-2(3H)-one (by-product).

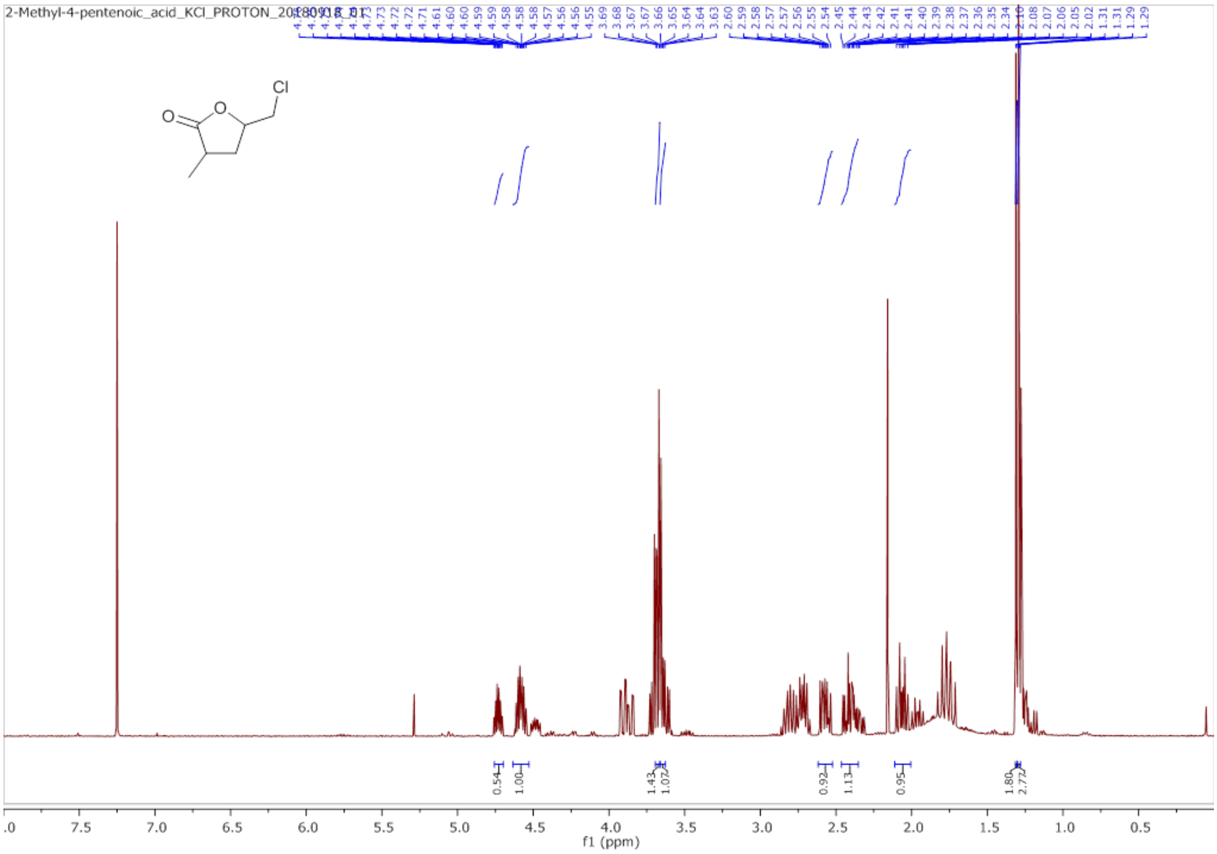

Scheme 10. ^{1}H NMR of 5-(chloromethyl)-3-methyldihydrofuran-2(3H)-one **(10b)**.

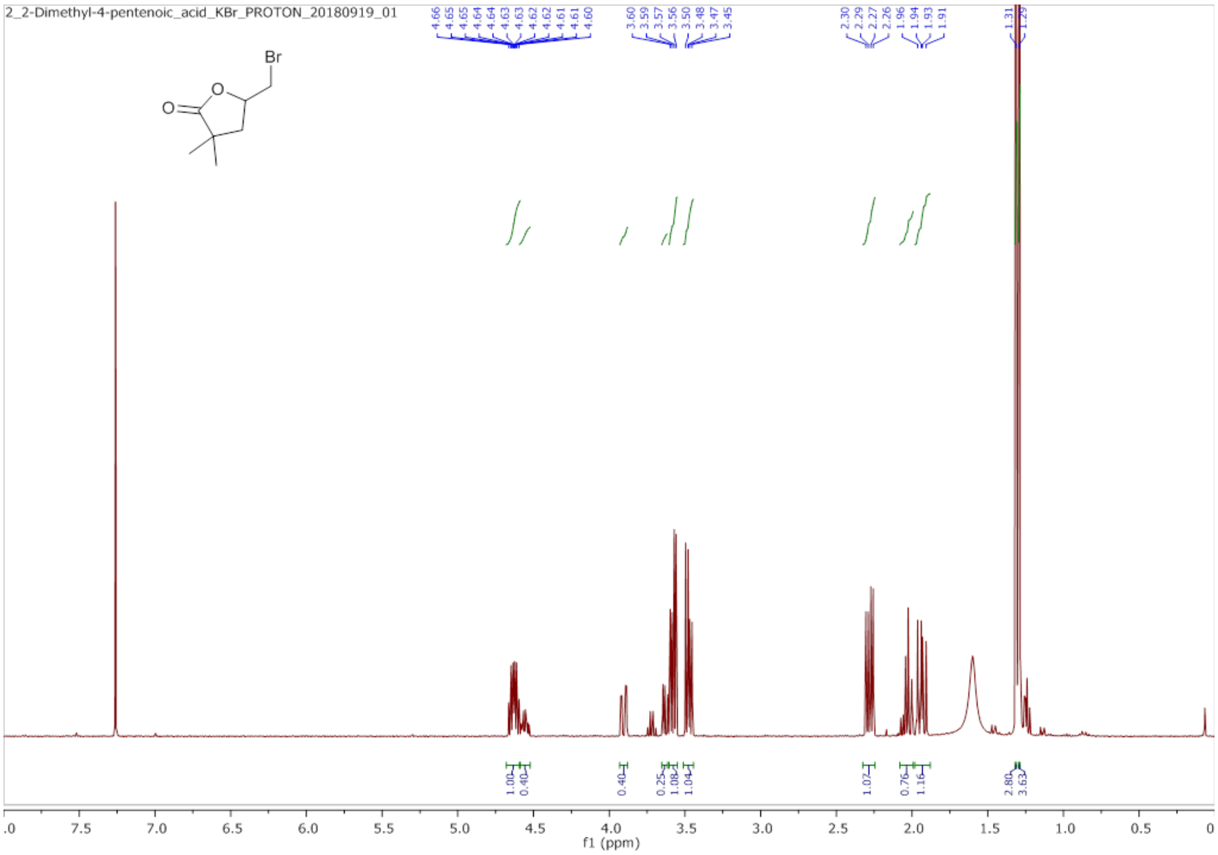

Scheme 11. ^{1}H NMR of 5-(bromomethyl)-3,3-dimethyldihydrofuran-2(3H)-one **(11a)**.

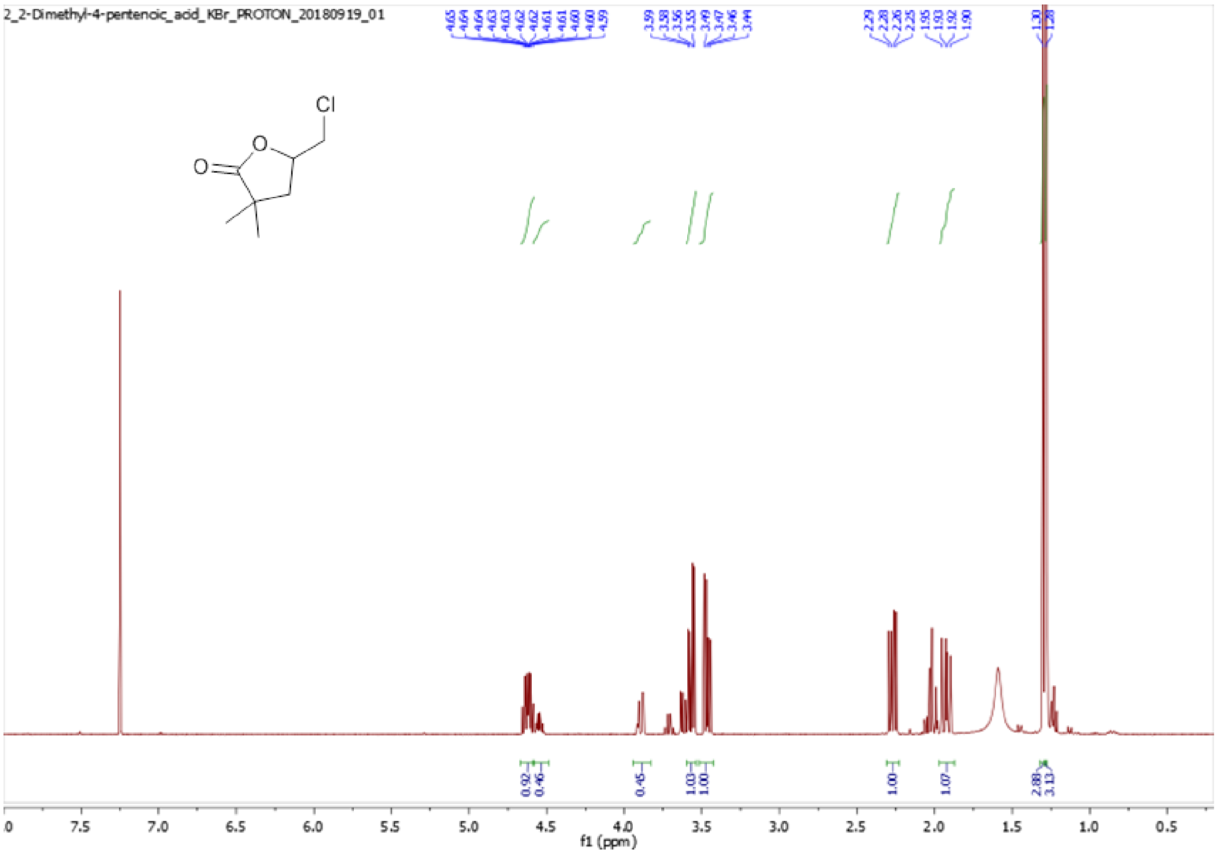

Scheme 12. ^{1}H NMR of 5-(chloromethyl)-3,3-dimethyldihydrofuran-2(3H)-one **(11b)**.

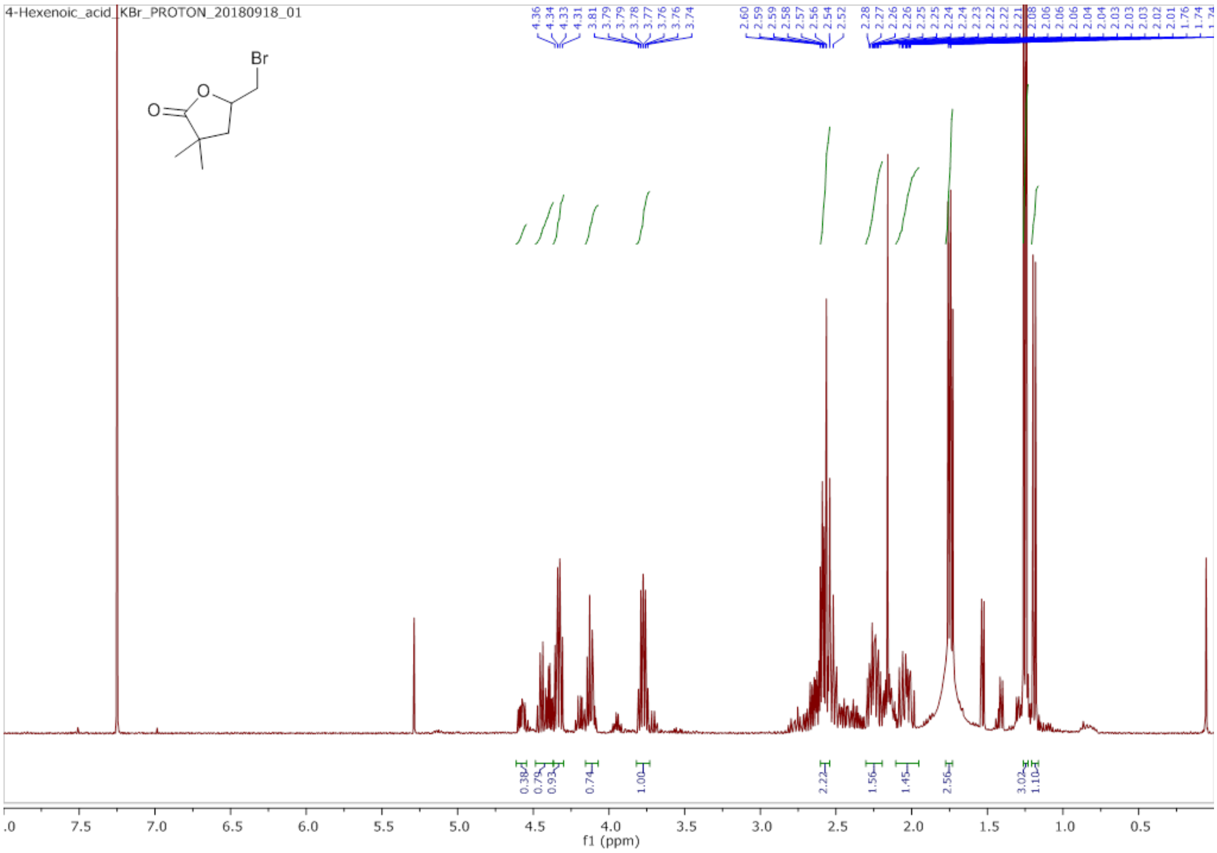

Scheme 13. ^{1}H NMR of 5-(1-bromoethyl)dihydrofuran-2(3H)-one **(12a)**.

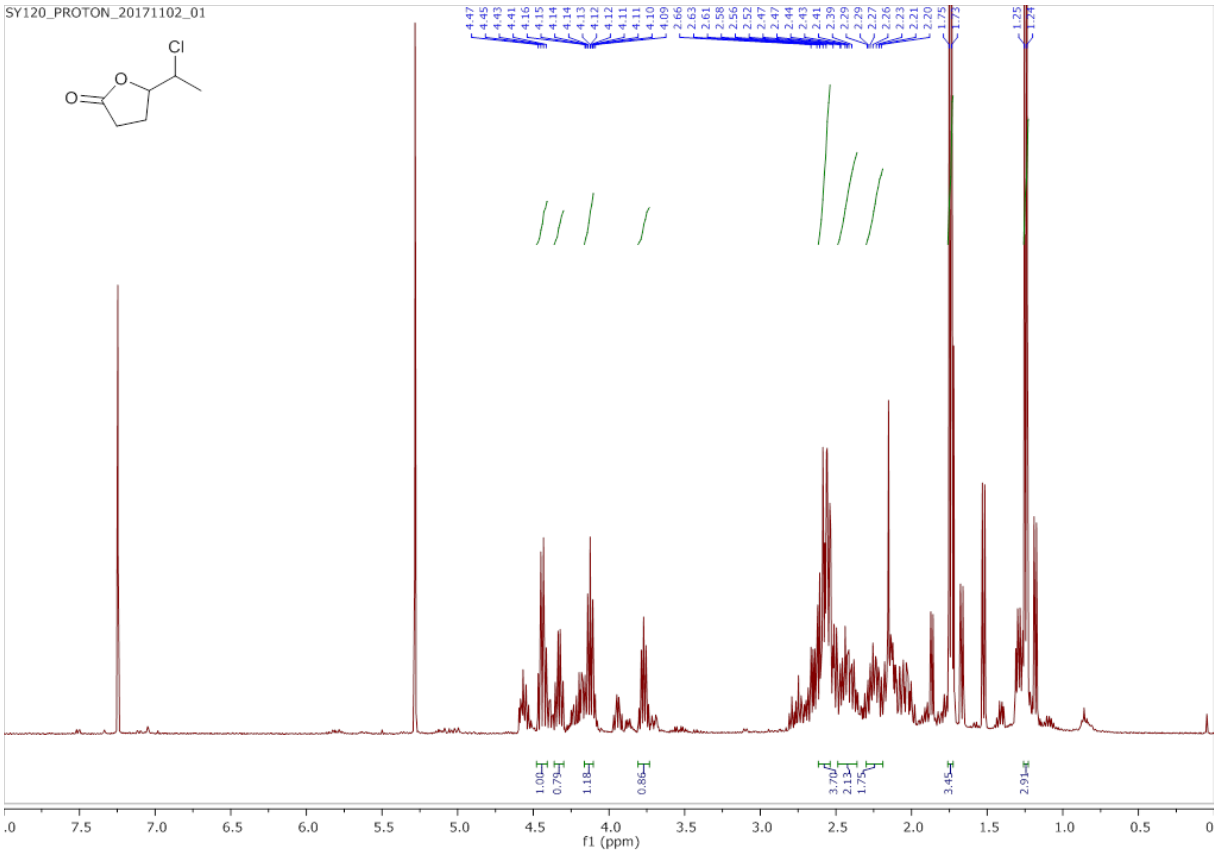

Scheme 14. ^{1}H NMR of 5-(1-chloroethyl)dihydrofuran-2(3H)-one **(12b)**.

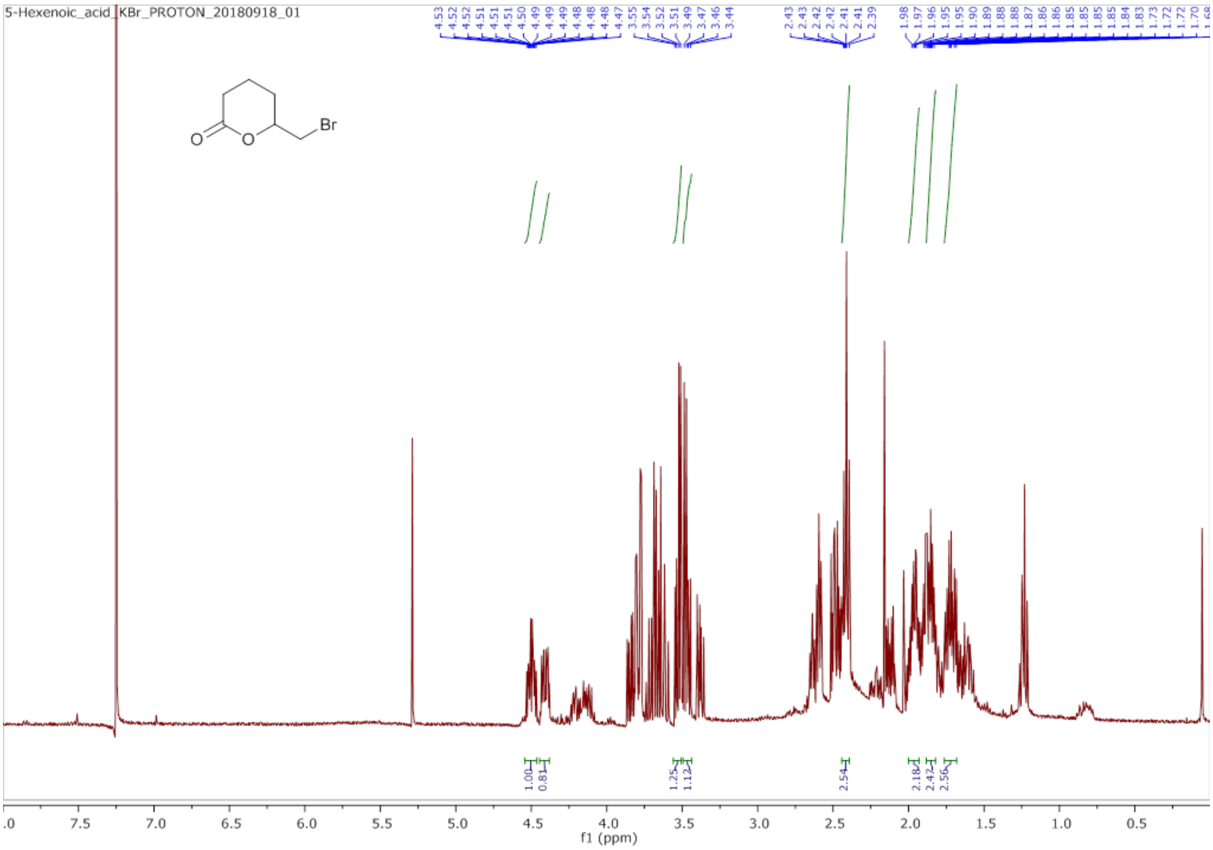

Scheme 15. ^{1}H NMR of 6-(bromomethyl)tetrahydro-2H-pyran-2-one **(13a)**.

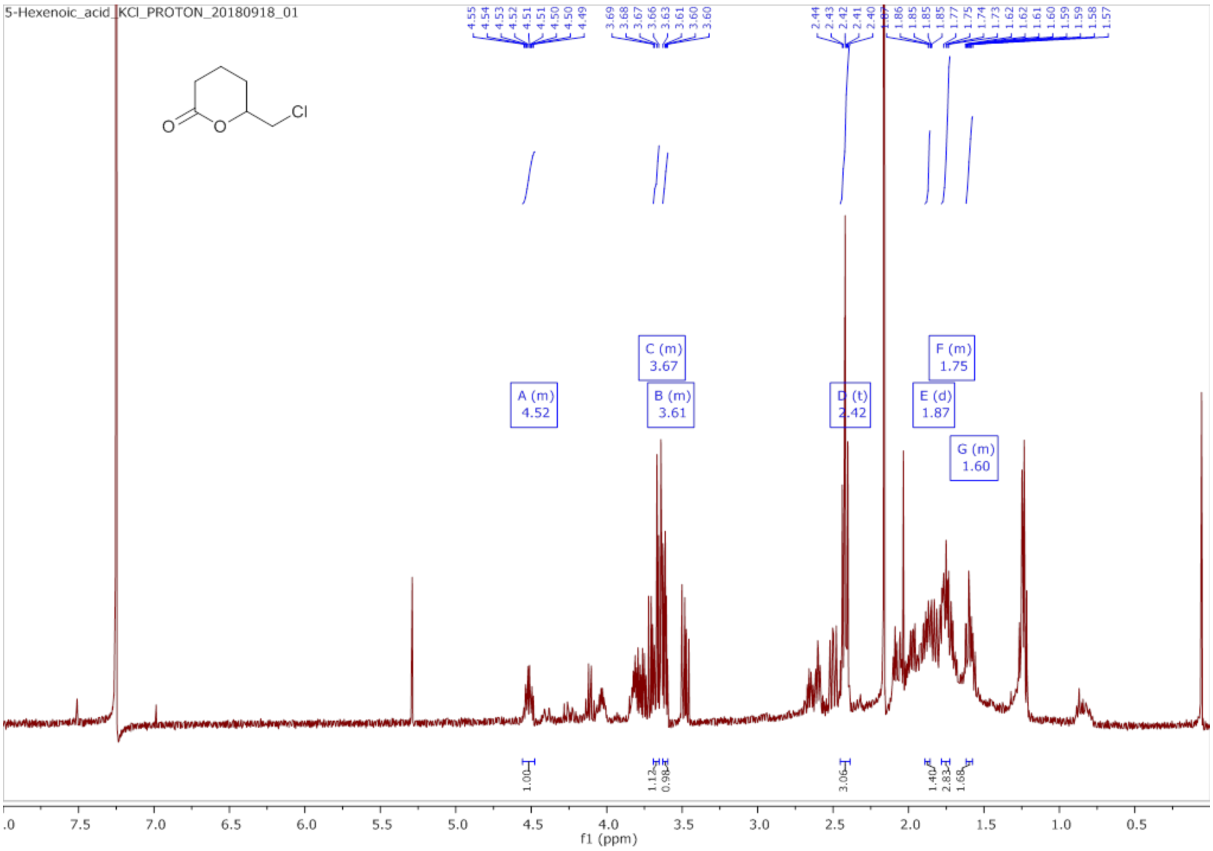

Scheme 16. ^{1}H NMR of 6-(chloromethyl)tetrahydro-2H-pyran-2-one **(13a)**.

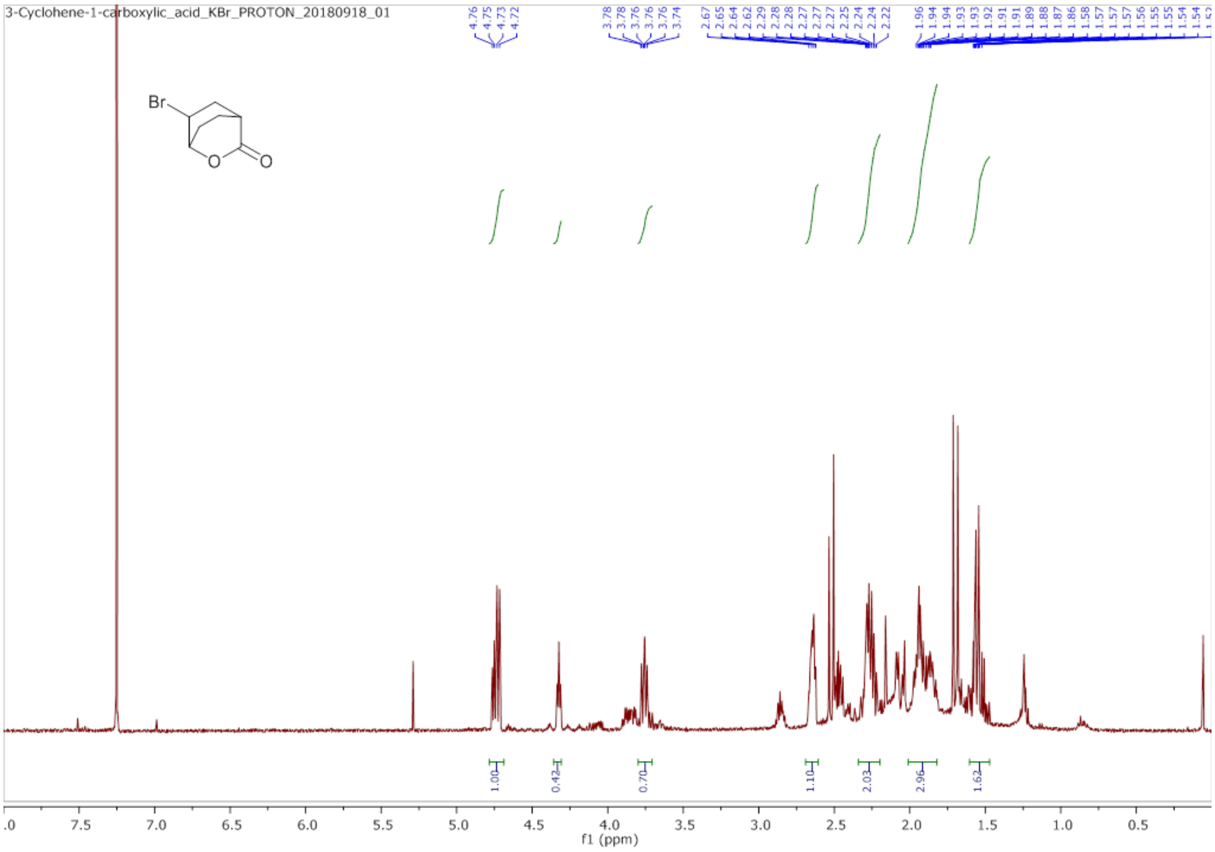

Scheme 17. ^{1}H NMR of 4-bromo-6-oxabicyclo[3.2.1]octan-7-one **(14a)**.

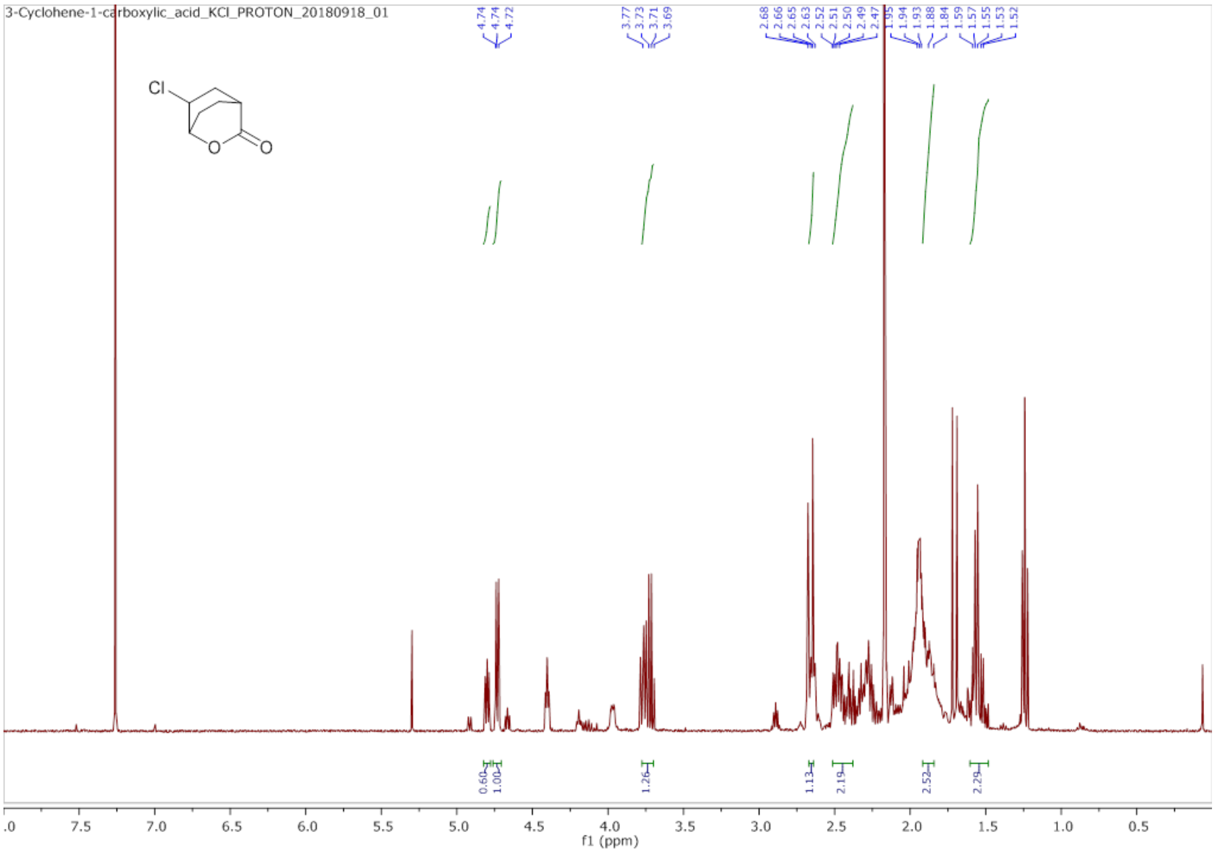

Scheme 18. ^{1}H NMR of 4-chloro-6-oxabicyclo[3.2.1]octan-7-one **(14b)**.

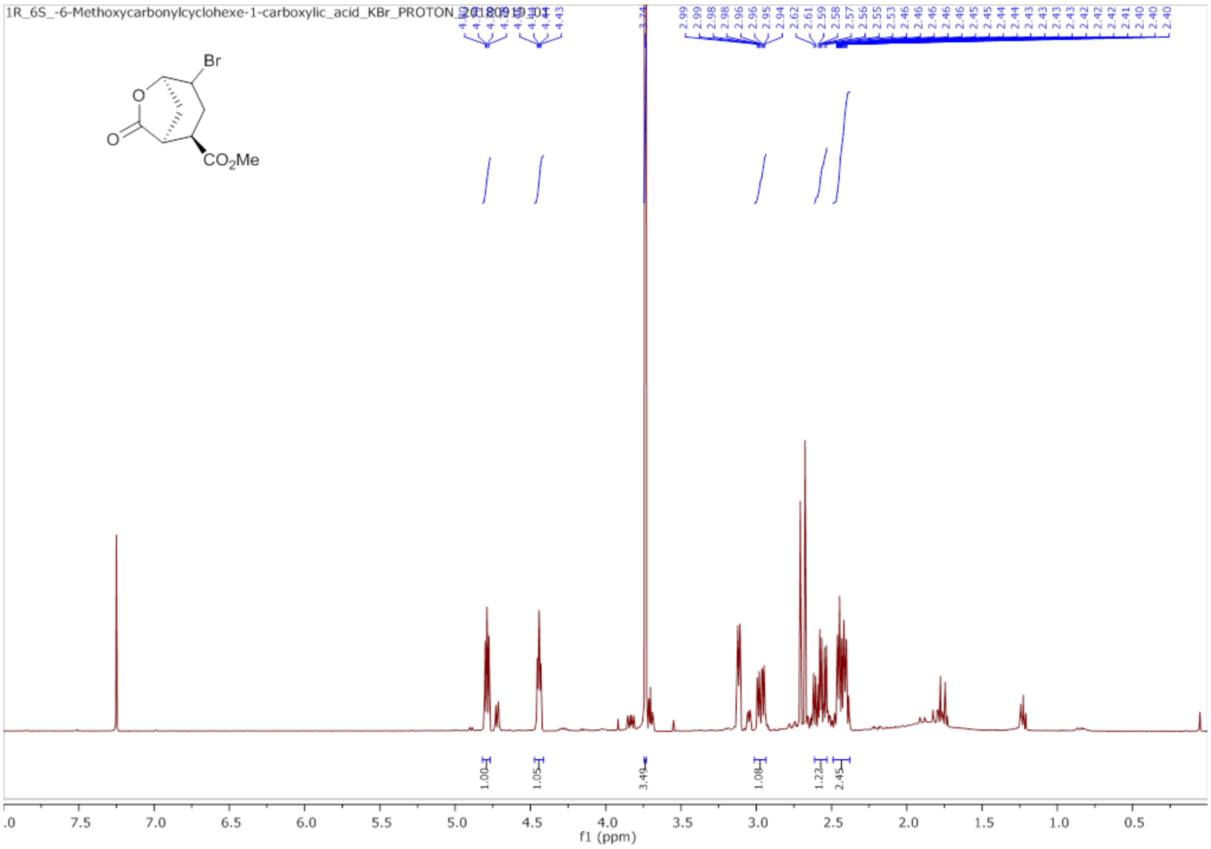

Scheme 19. ^{1}H NMR of methyl (1R,2S)-4-bromo-7-oxo-6-oxabicyclo[3.2.1]octane-2-carboxylate **(15a)**.

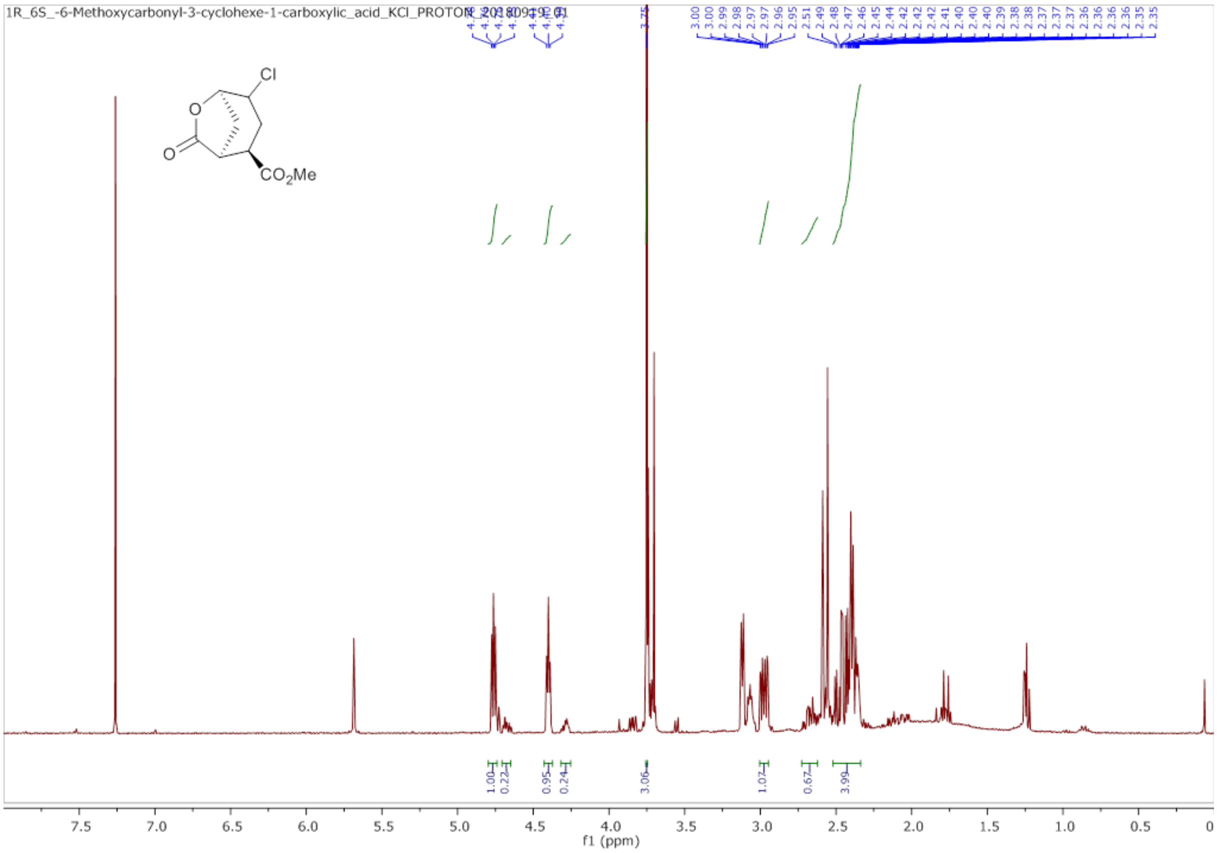

Scheme 20. ^{1}H NMR of methyl (1R,2S)-4-chloro-7-oxo-6-oxabicyclo[3.2.1]octane-2-carboxylate **(15b)**.

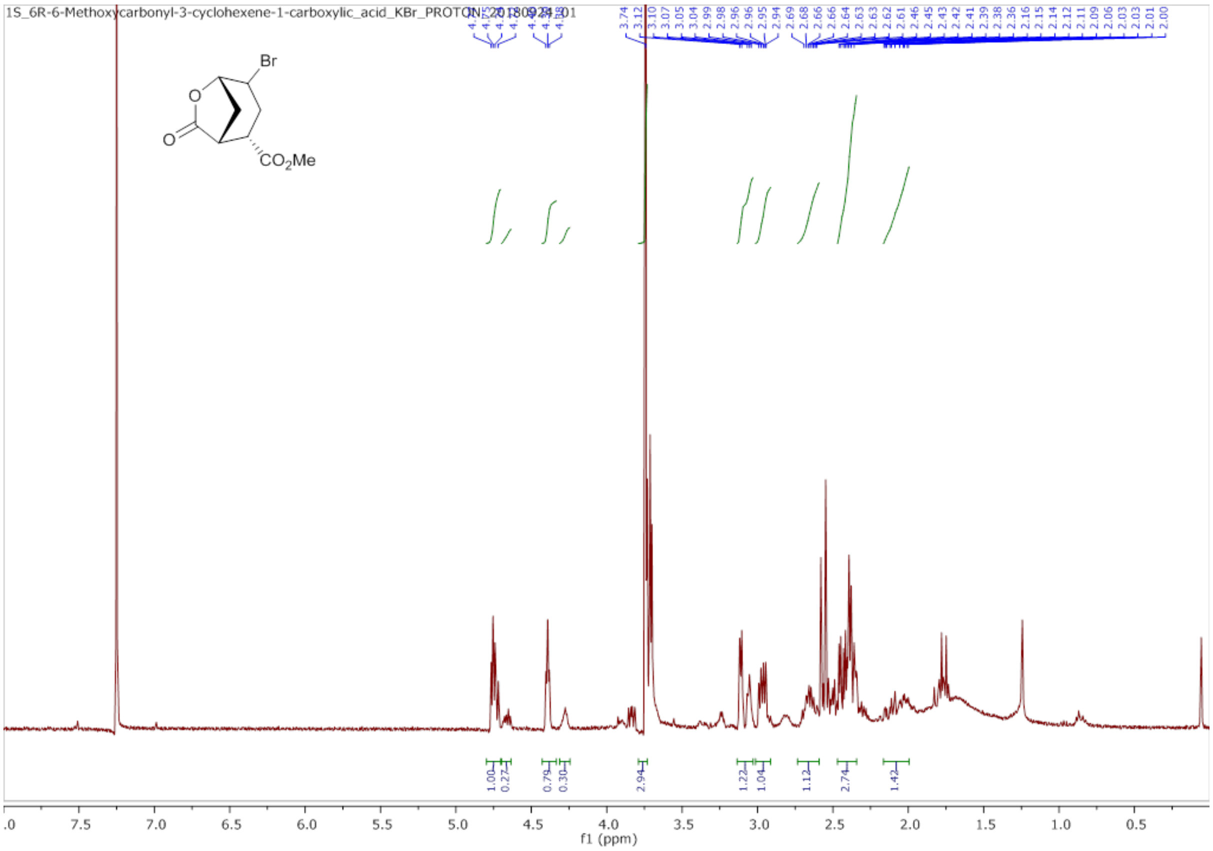

Scheme 21. ^{1}H NMR of methyl (1R,2R)-4-bromo-7-oxo-6-oxabicyclo[3.2.1]octane-2-carboxylate **(16a)**.

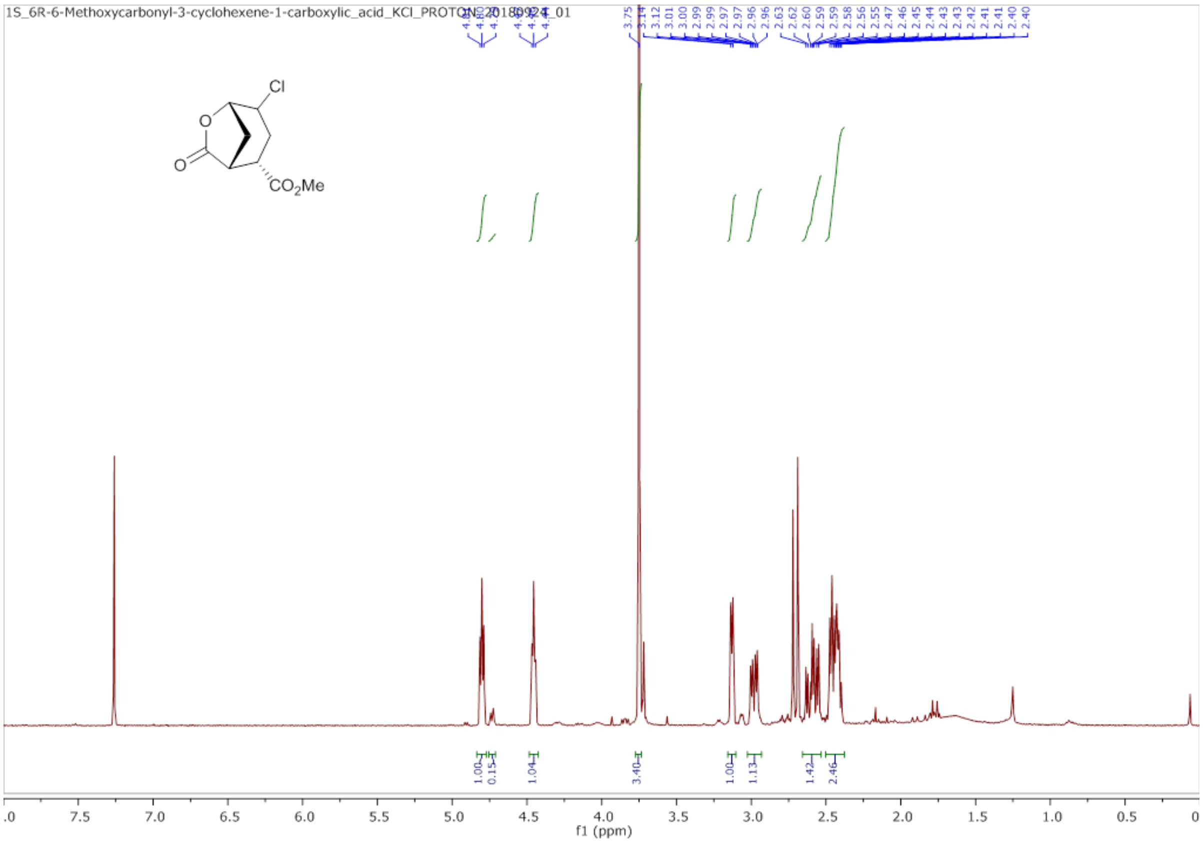

Scheme 22. ^{1}H NMR of methyl (1R,2R)-4-chloro-7-oxo-6-oxabicyclo[3.2.1]octane-2-carboxylate **(16b)**.

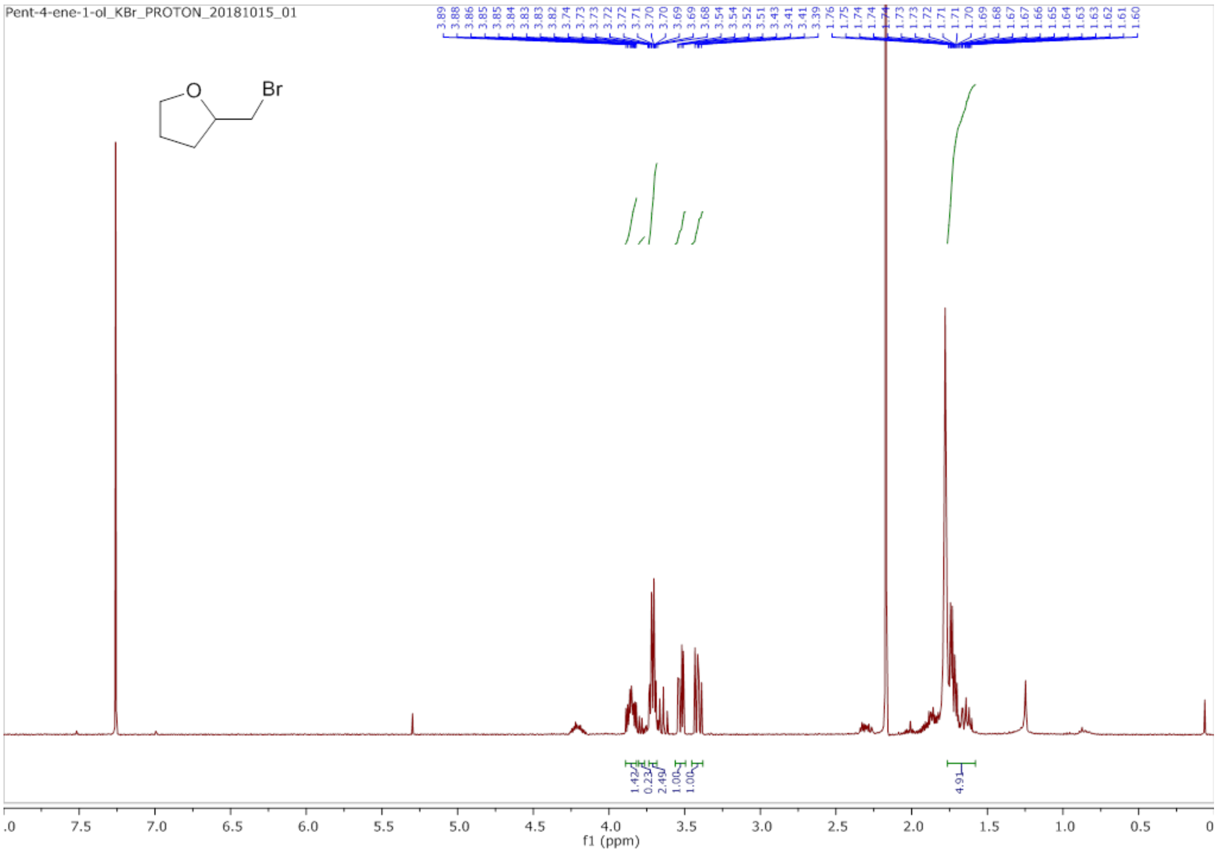

Scheme 23. ^{1}H NMR of 2-(bromomethyl)tetrahydrofuran **(17a)**.

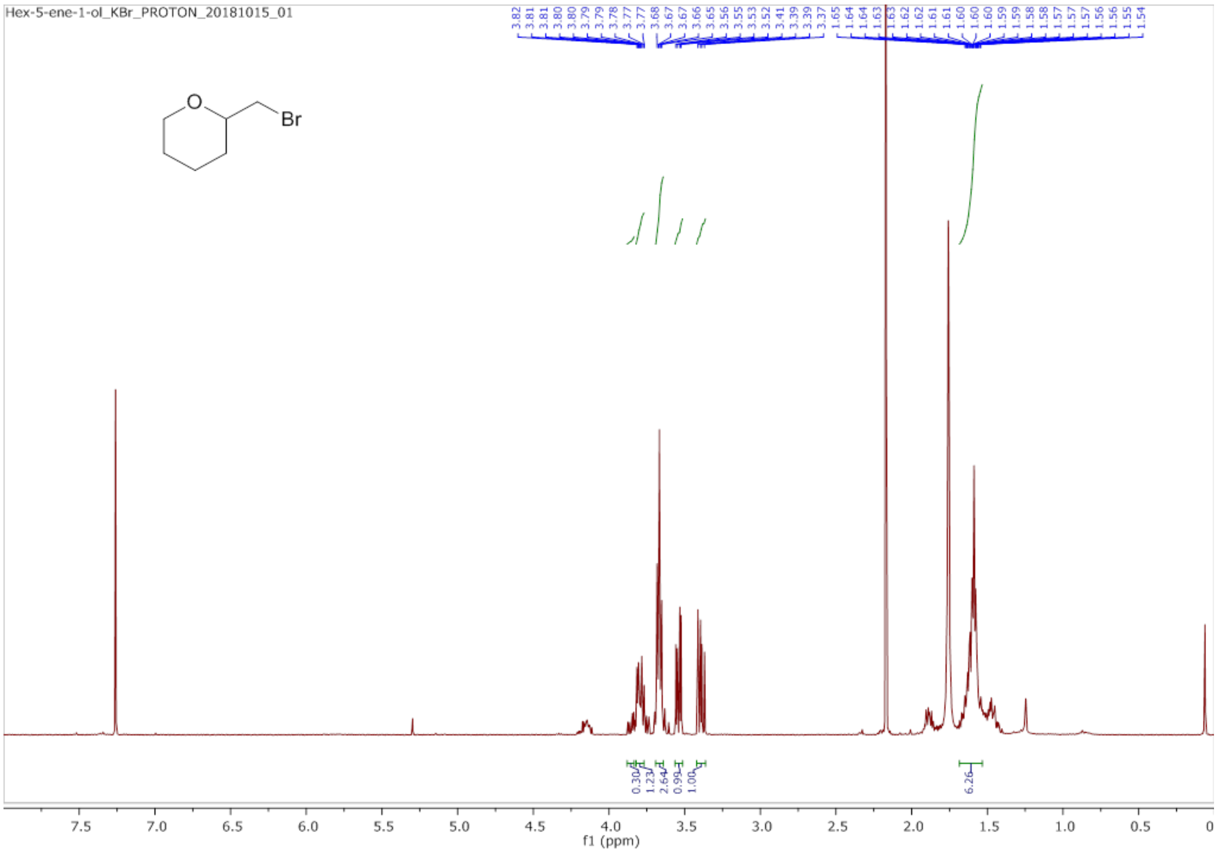

Scheme 24. ^{1}H NMR of 2-(bromomethyl)tetrahydro-2H-pyran **(18a)**.

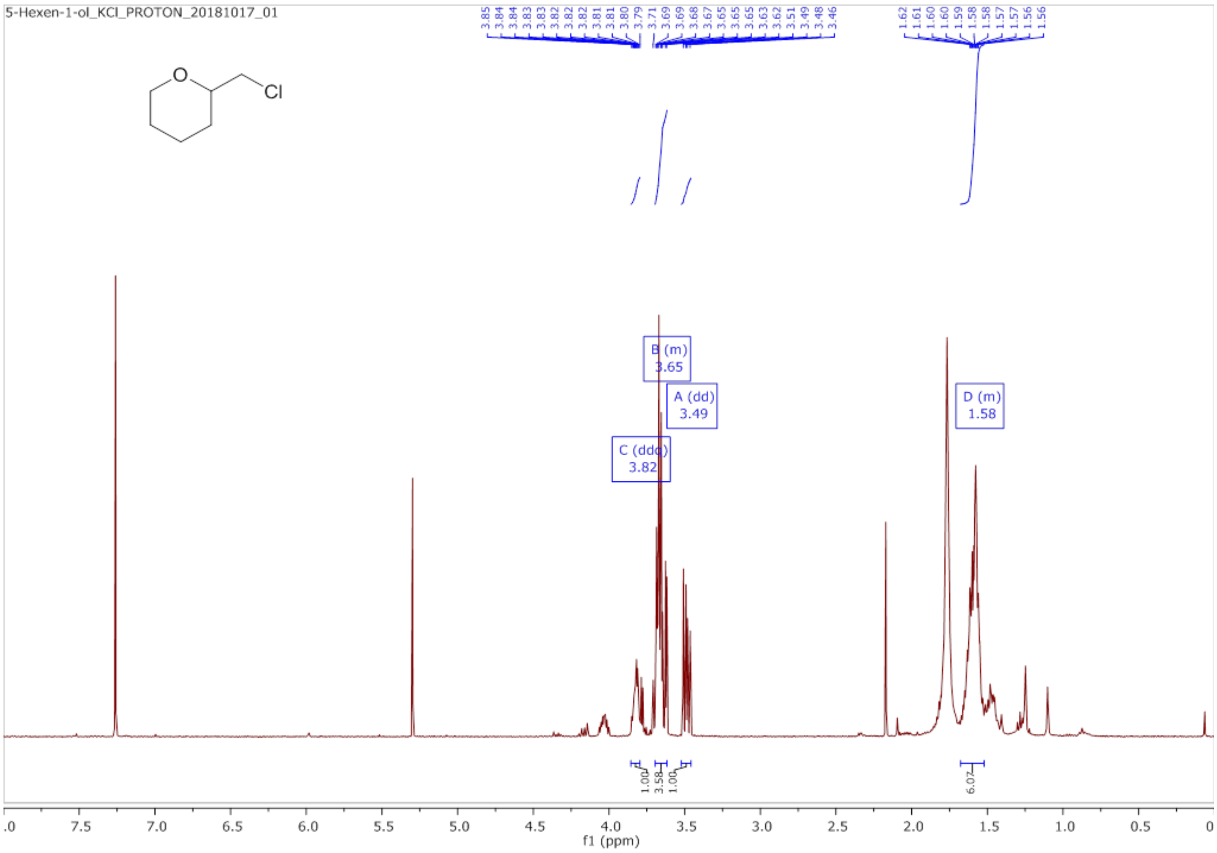

Scheme 25. ^{1}H NMR of 2-(chloromethyl)tetrahydro-2H-pyran **(18b)**.

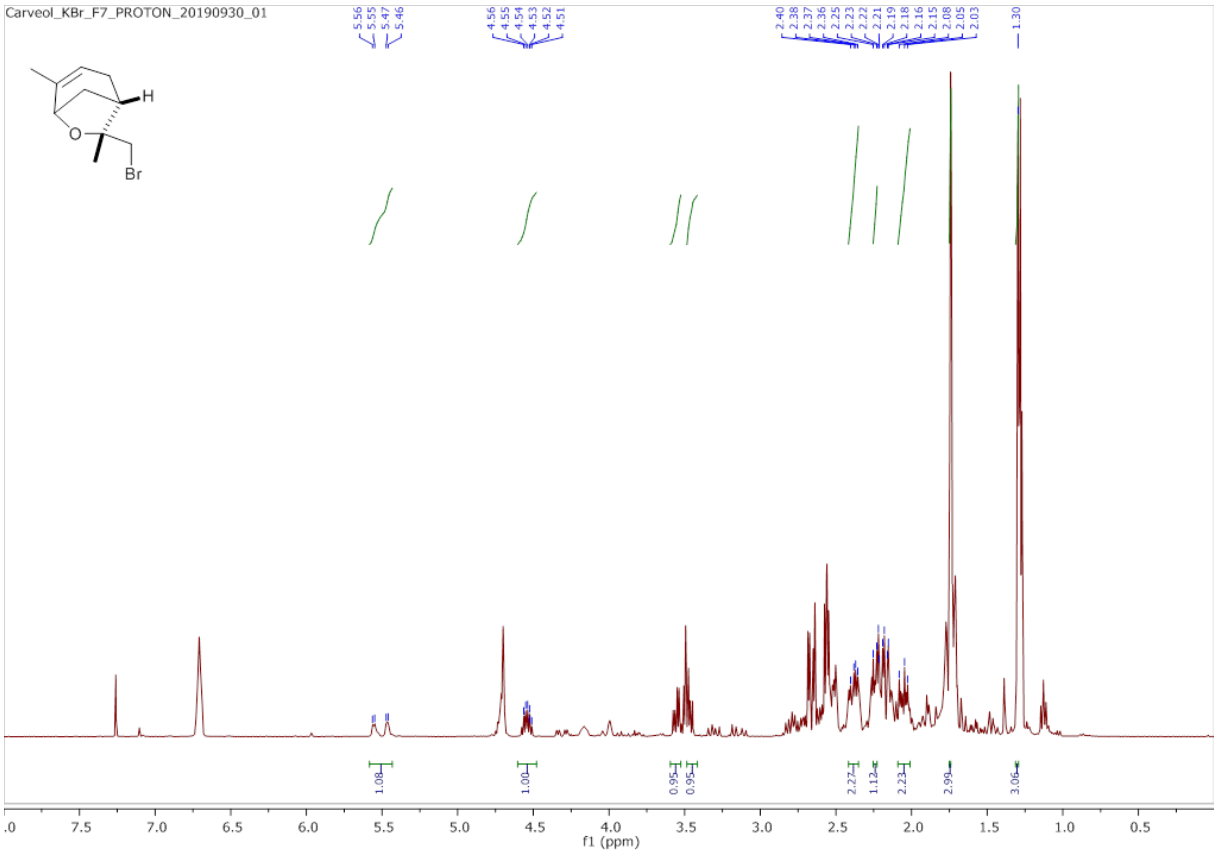

Scheme 26. ^{1}H NMR of 7-(Bromomethyl)-4,7-dimethyl-6-oxabicyclo[3.2.1]oct-3-ene (**19a**).

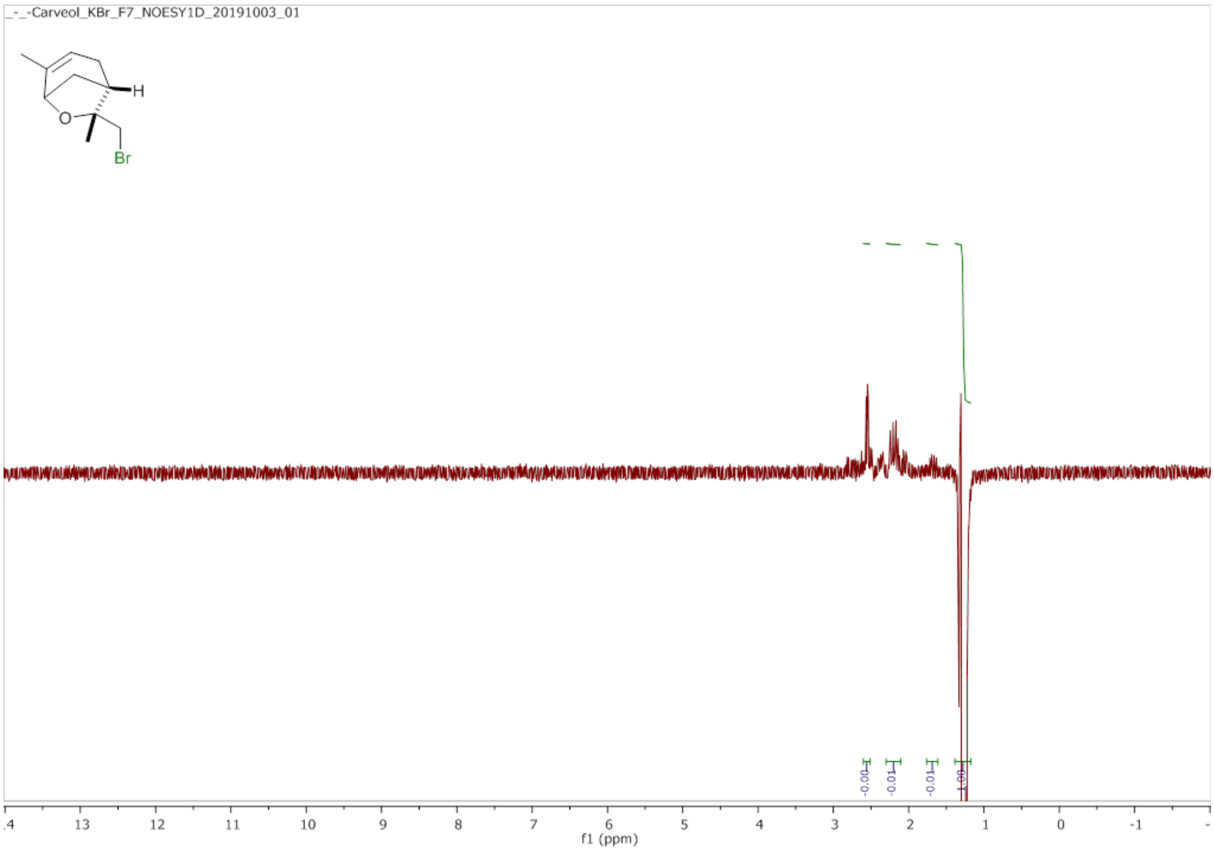

Scheme 27. NOESY1D (NOE) of 7-(Bromomethyl)-4,7-dimethyl-6-oxabicyclo[3.2.1]oct-3-ene (**19a**).

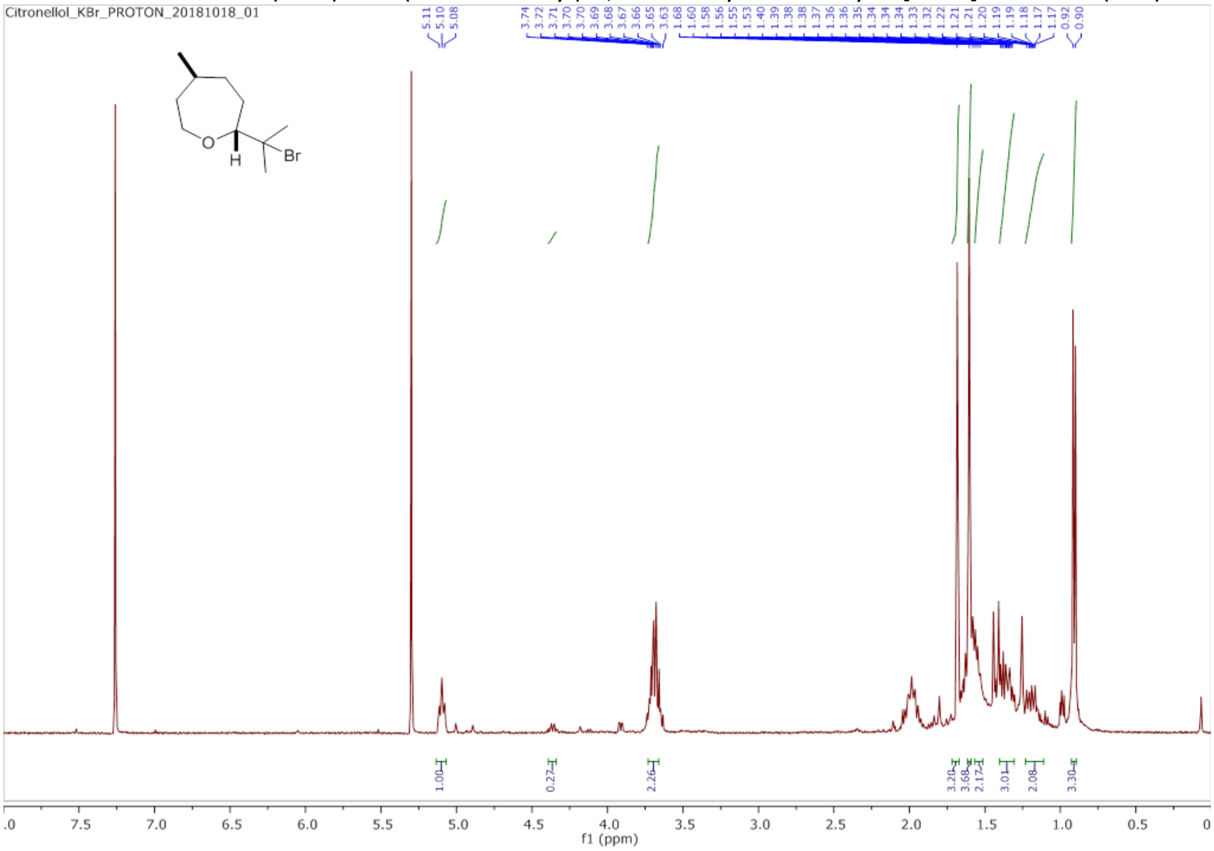

Scheme 28. ^{1}H NMR of 2-(2-bromopropan-2-yl)-5-methyloxepane **(20a)**.

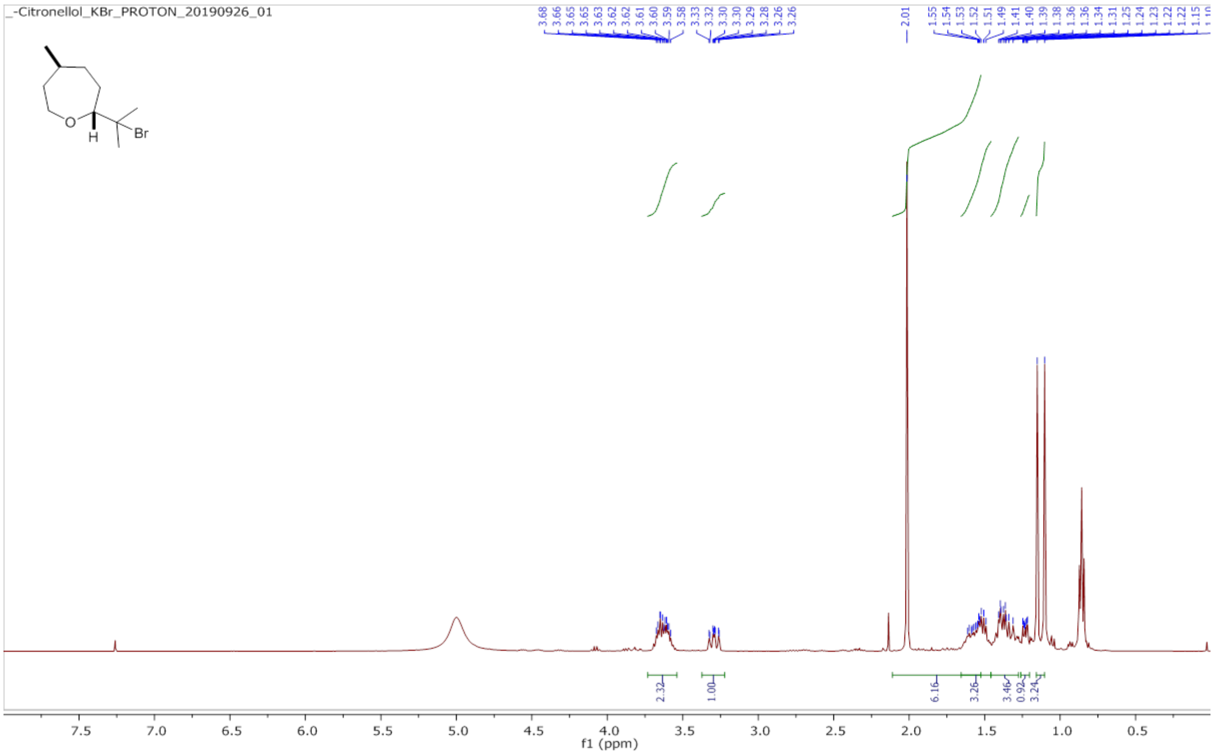

Scheme 29. ^{1}H NMR of pure 2-(2-bromopropan-2-yl)-5-methyloxepane **(20a)**.

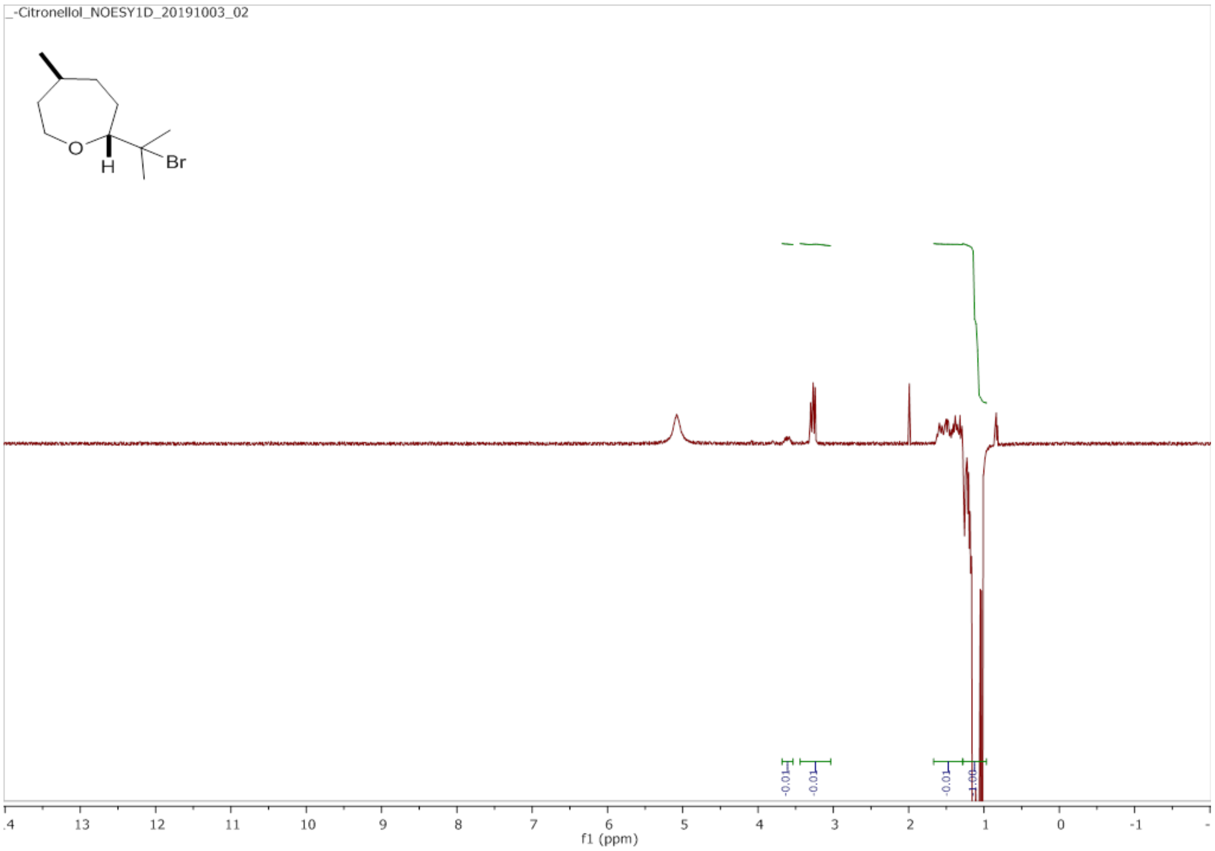

Scheme 30. NOESY1D (NOE) of pure 2-(2-bromopropan-2-yl)-5-methyloxepane **(20a)**.

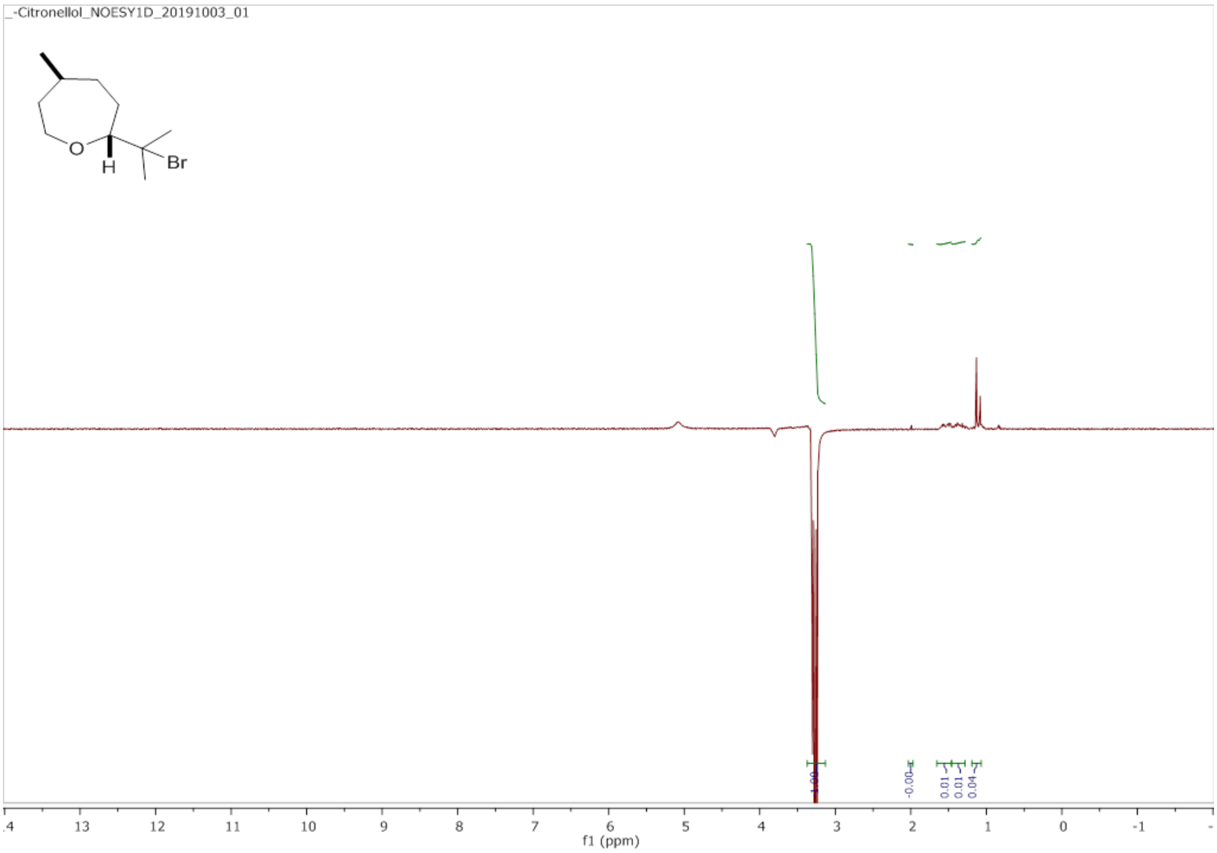

Scheme 31. NOESY1D (NOE) of pure 2-(2-bromopropan-2-yl)-5-methyloxepane **(20a)**.

7.5. GC-Chromatograms

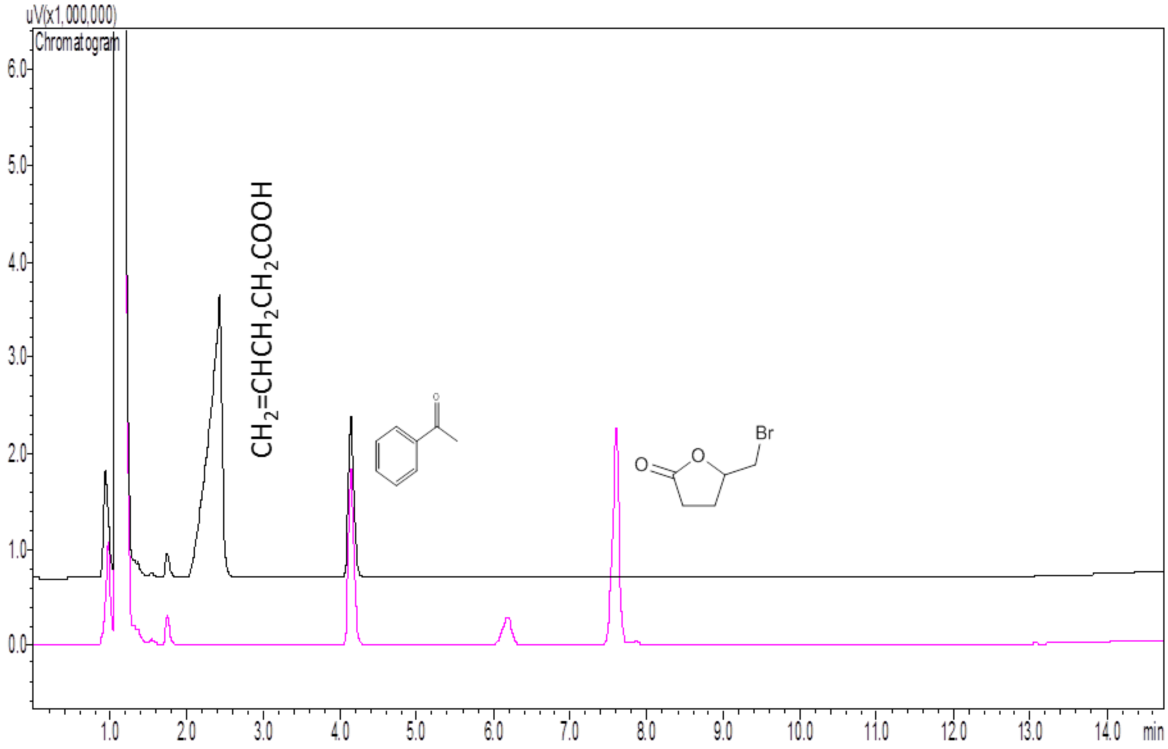

Figure S1. Representative GC chromatogram of 5-(bromomethyl)dihydrofuran-2(3H)-one **(9a)**.

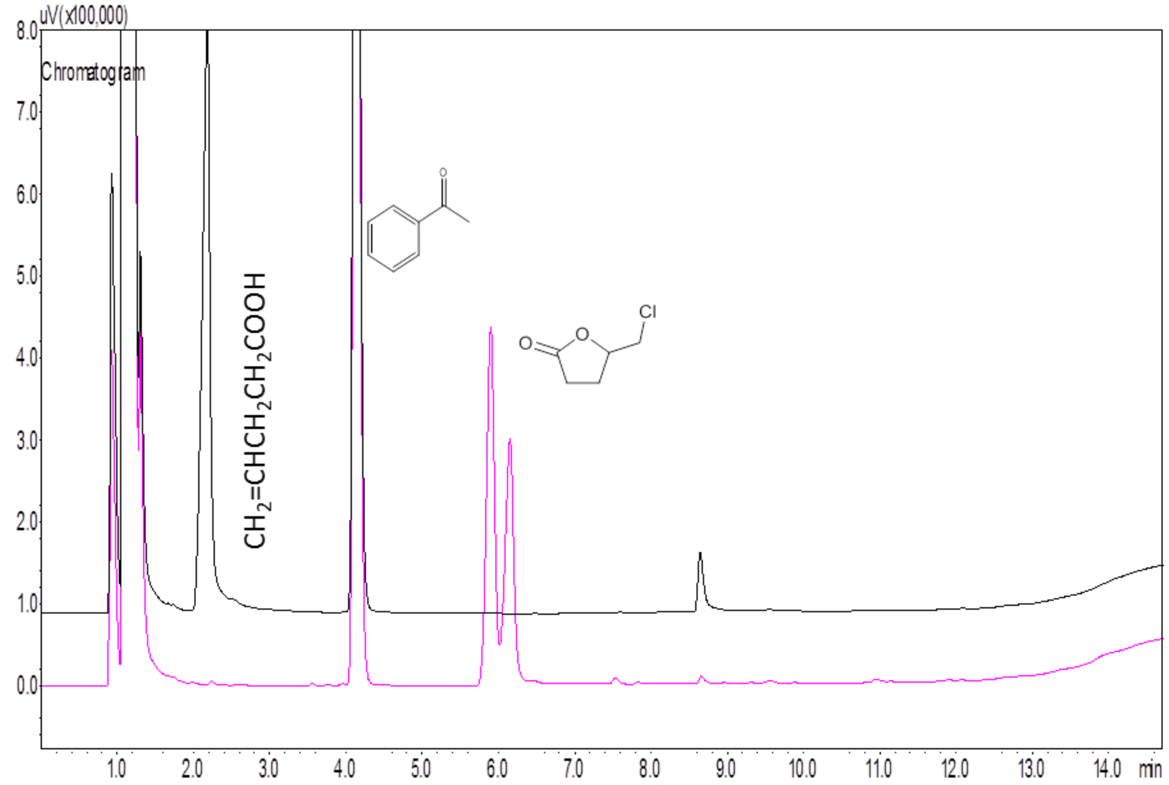

Figure S2. Representative GC chromatogram of 5-(chloromethyl)dihydrofuran-2(3H)-one **(9b)**.

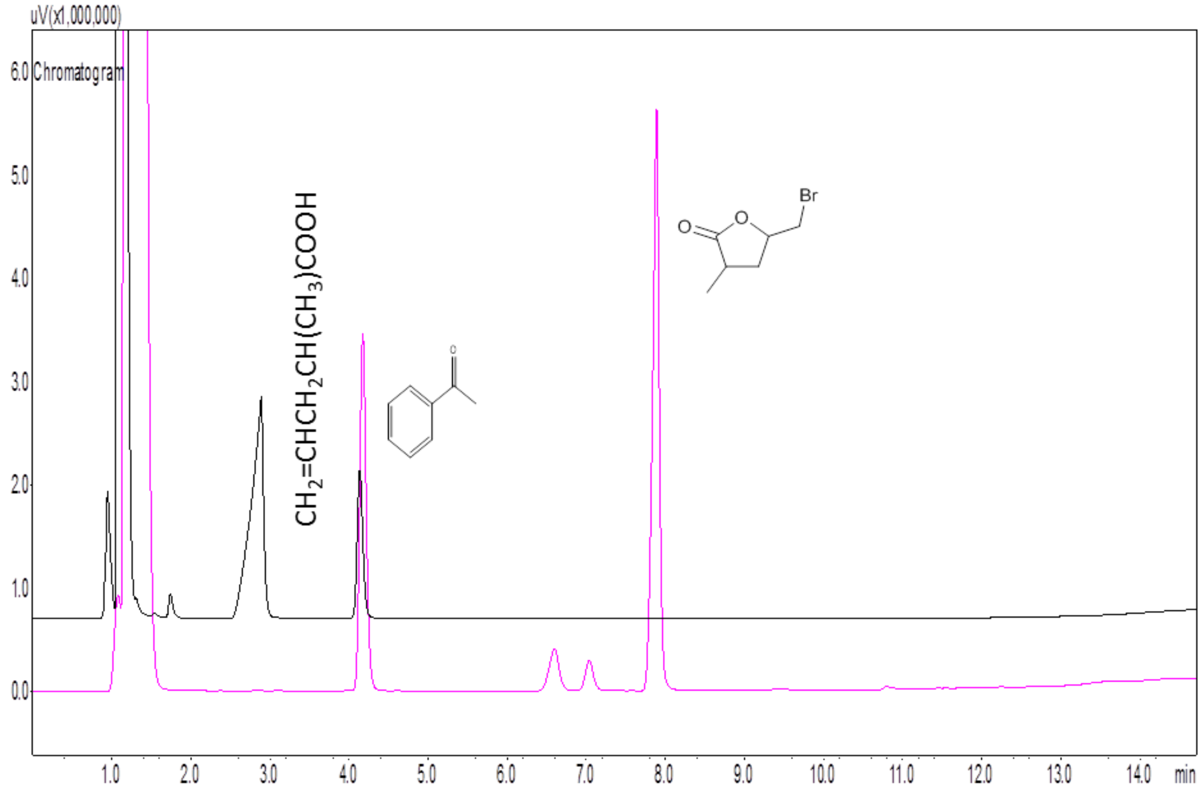

Figure S3. Representative GC chromatogram of 5-(bromomethyl)-3-methyldihydrofuran-2(3H)-one **(10a)**.

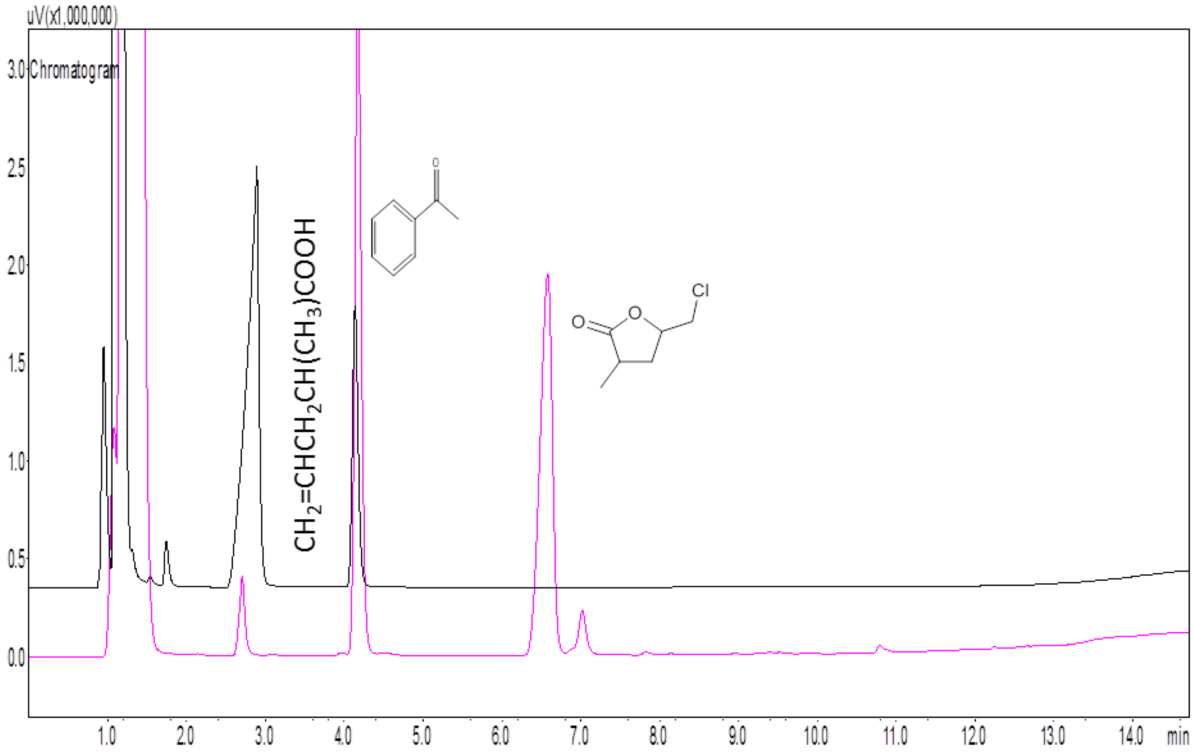

Figure S4. Representative GC chromatogram of 5-chloromethyl)-3-methyldihydrofuran-2(3H)-one **(10b)**.

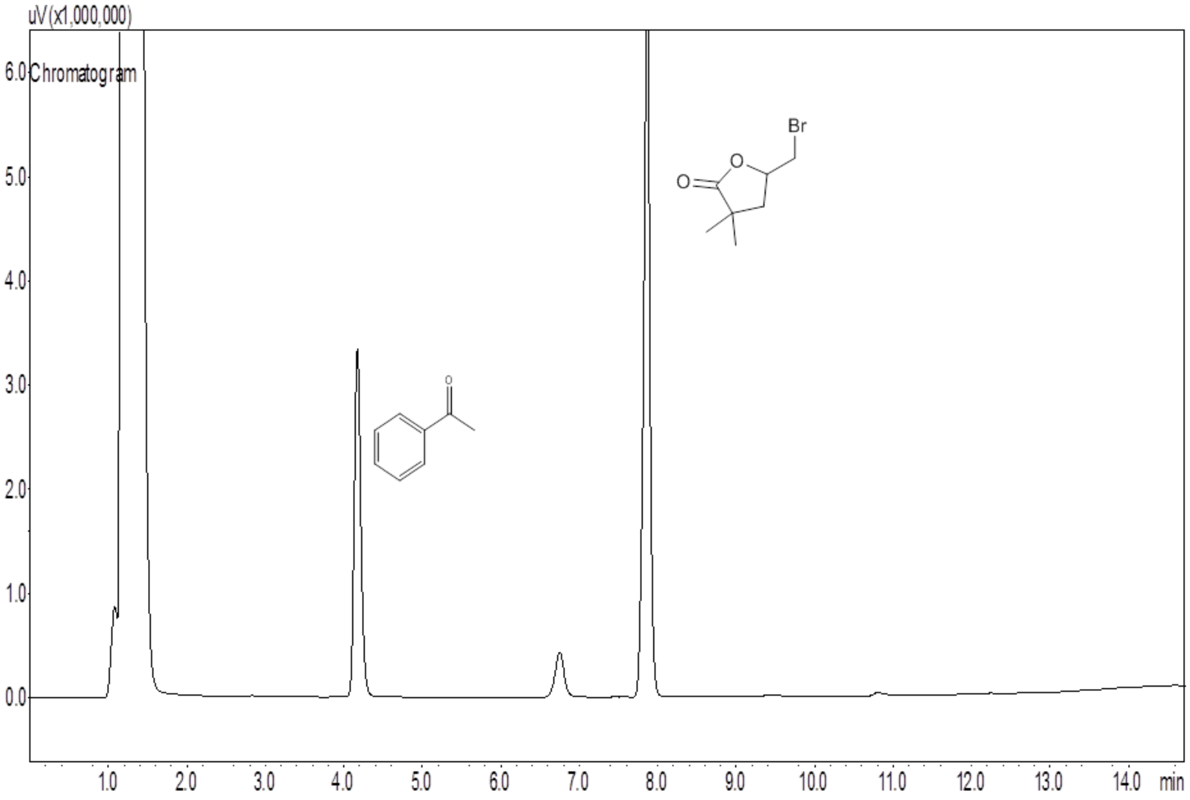

Figure S5. Representative GC chromatogram of 5-(bromomethyl)-3,3-dimethyldihydrofuran-2(3H)-one **(11a)**.

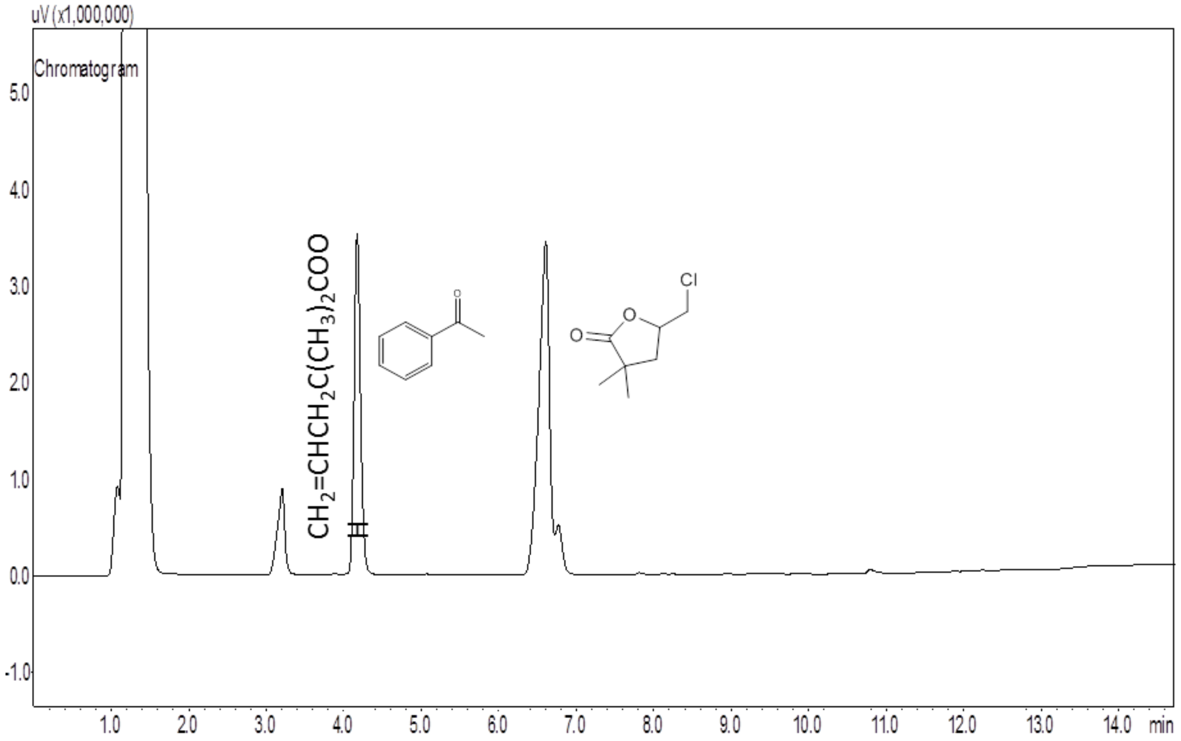

Figure S6. Representative GC chromatogram of 5-chloromethyl)-3,3-dimethyldihydrofuran-2(3H)-one **(11b)**.

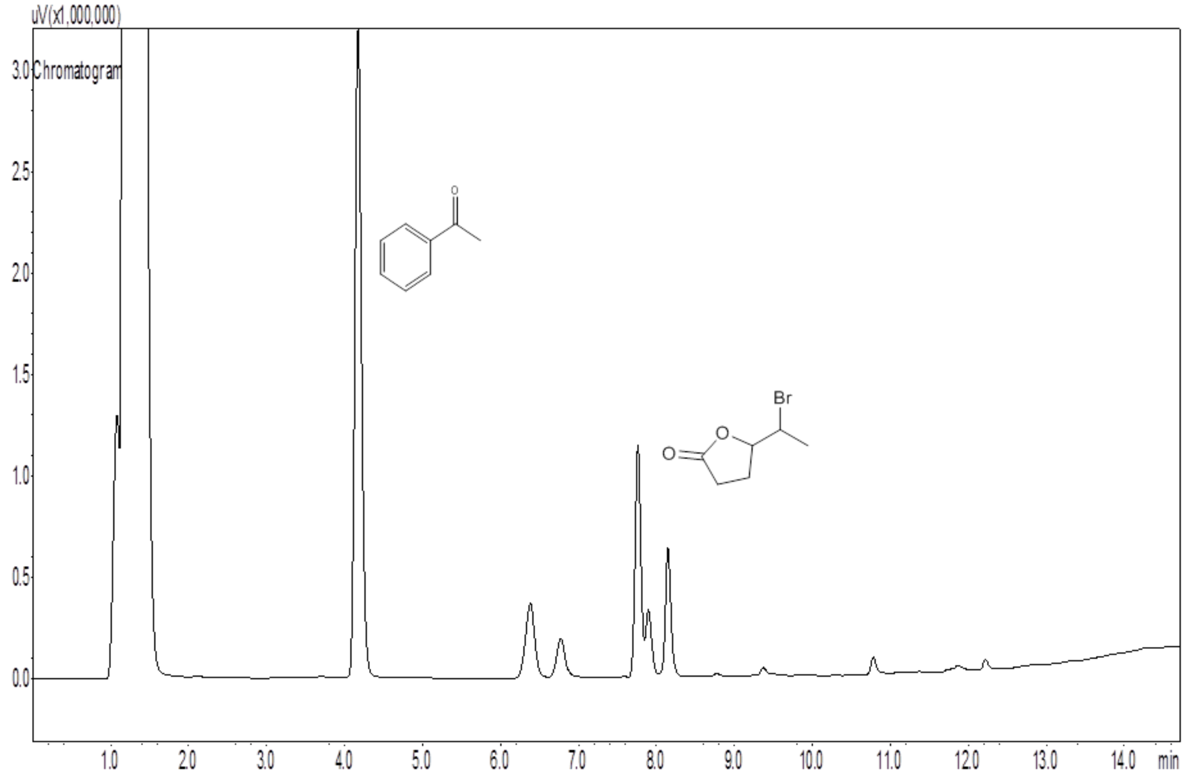

Figure S7. Representative GC chromatogram of 5-(1-bromoethyl)dihydrofuran-2(3H)-one **(12a)**.

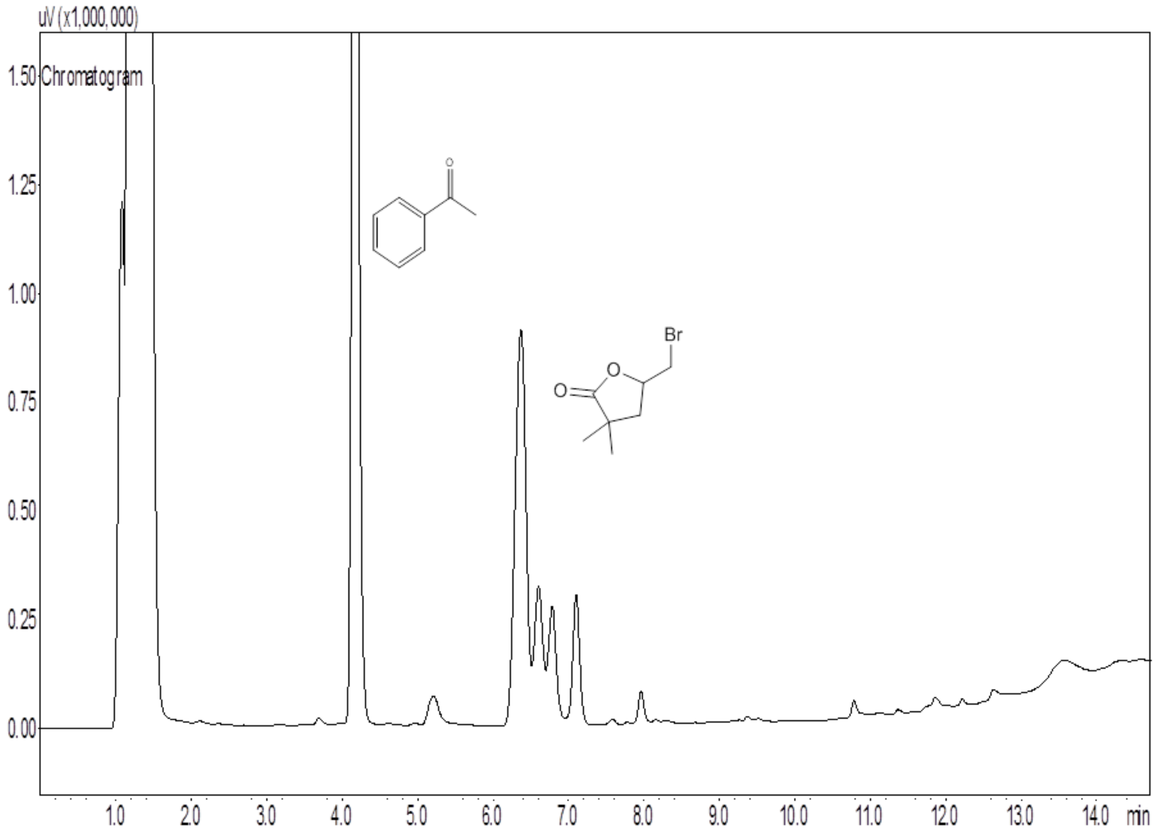

Figure S8. Representative GC chromatogram of 5-(1-chloroethyl)dihydrofuran-2(3H)-one **(12b).**

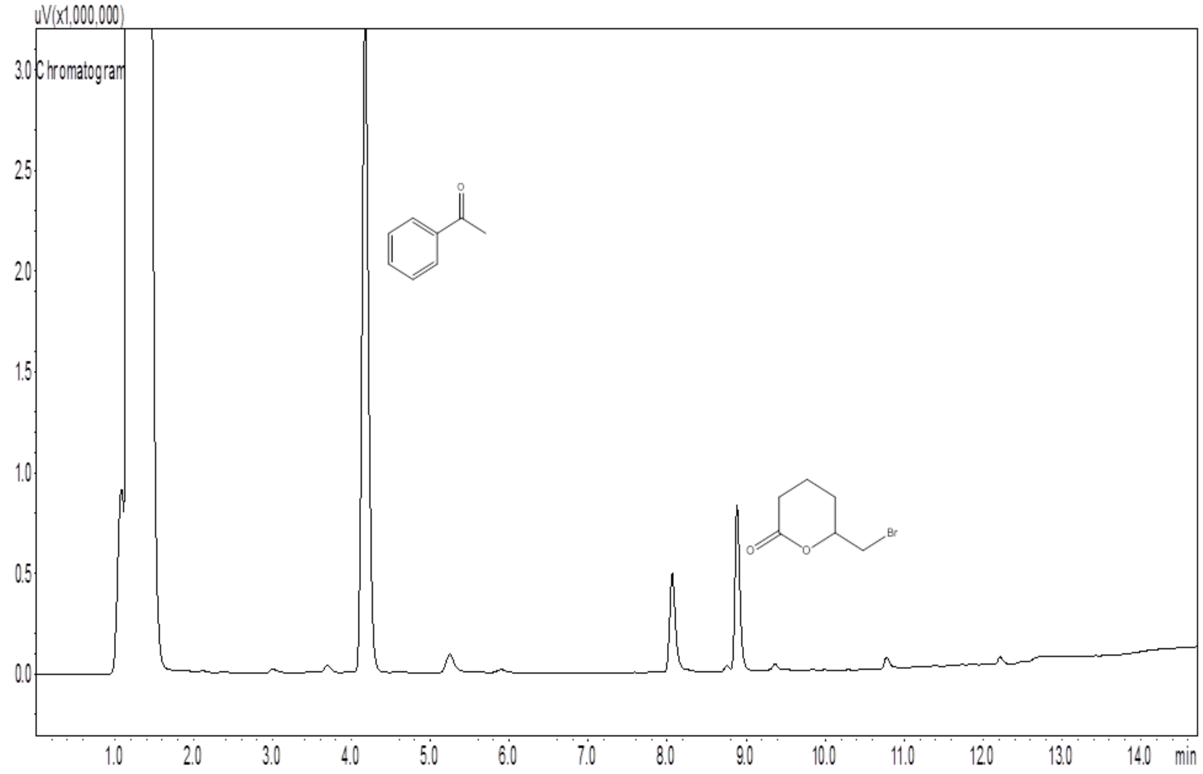

Figure S9. Representative GC chromatogram of 6-(bromomethyl)tetrahydro-2H-pyran-2-one **(13a)**.

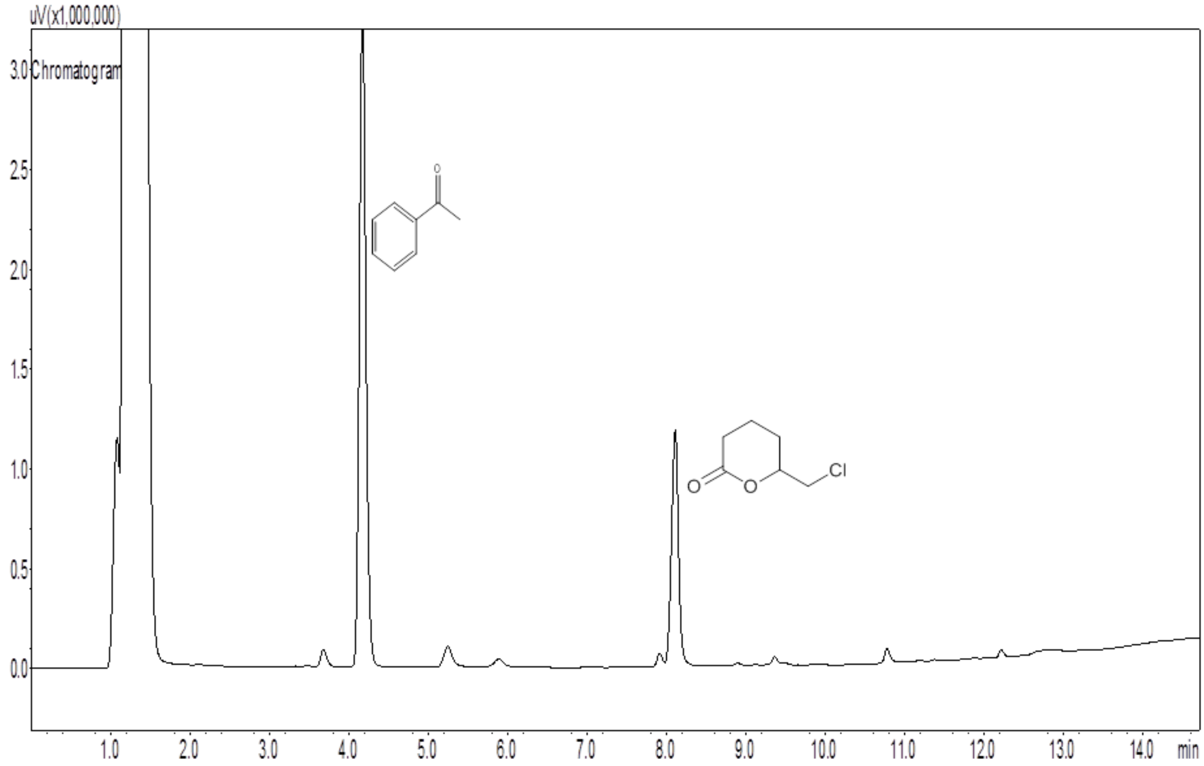

Figure S10. Representative GC chromatogram of 6-(chloromethyl)tetrahydro-2H-pyran-2-one **(13b)**.

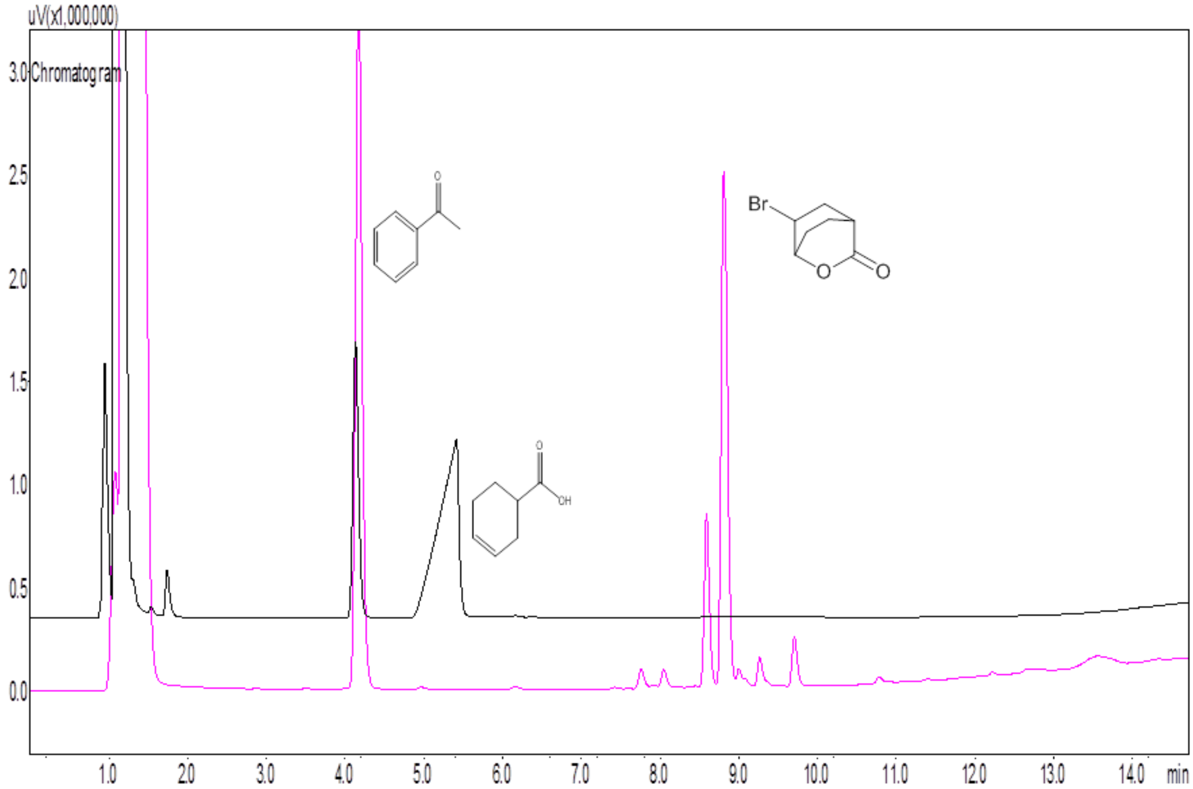

Figure S11. Representative GC chromatogram of 4-bromo-6-oxabicyclo[3.2.1]octan-7-one **(14a)**.

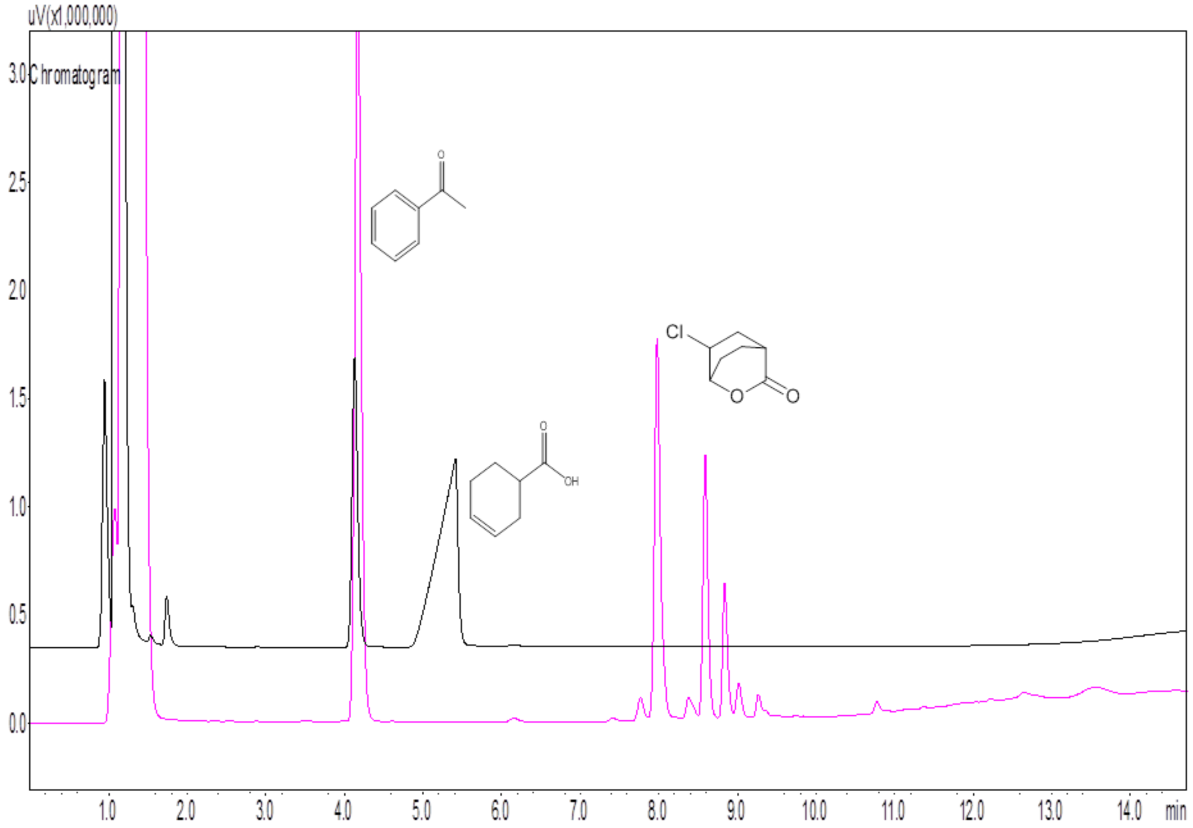

Figure S12. Representative GC chromatogram of 4-chloro-6-oxabicyclo[3.2.1]octan-7-one **(14b)**.

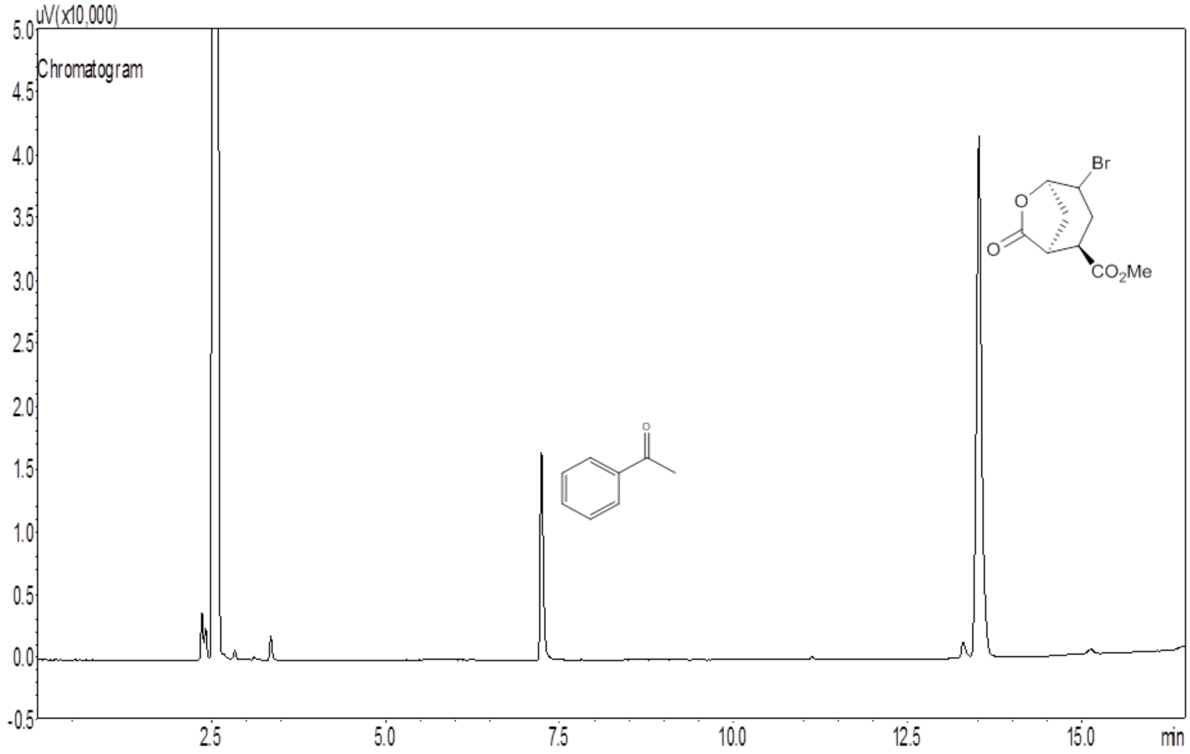

Figure S13. Representative GC chromatogram of methyl (1R,2S)-4-bromo-7-oxo-6-oxabicyclo[3.2.1]octane-2-carboxylate **(15a)**.

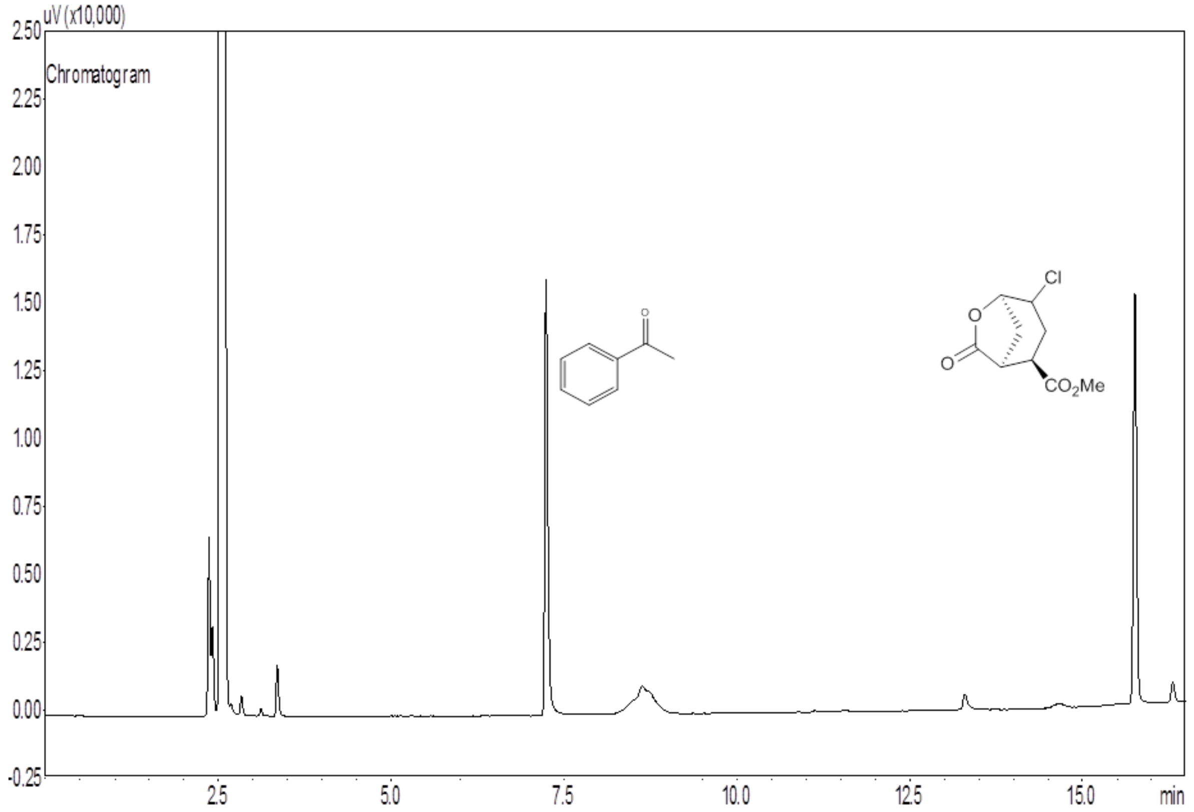

Figure S14. Representative GC chromatogram of methyl (1R,2S)-4-chloro-7-oxo-6-oxabicyclo[3.2.1]octane-2-carboxylate **(15b).**

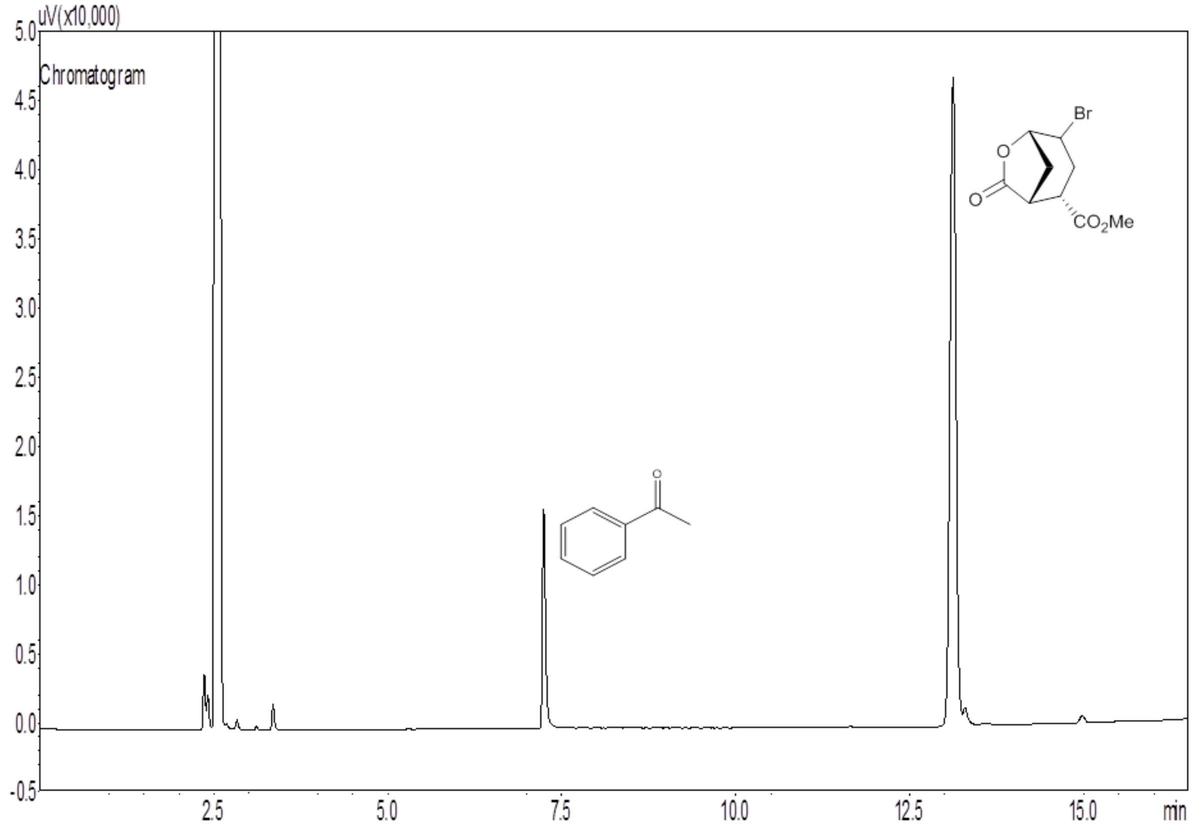

Figure S15. Representative GC chromatogram of methyl (1R,2R)-4-bromo-7-oxo-6-oxabicyclo[3.2.1]octane-2-carboxylate **(16a)**.

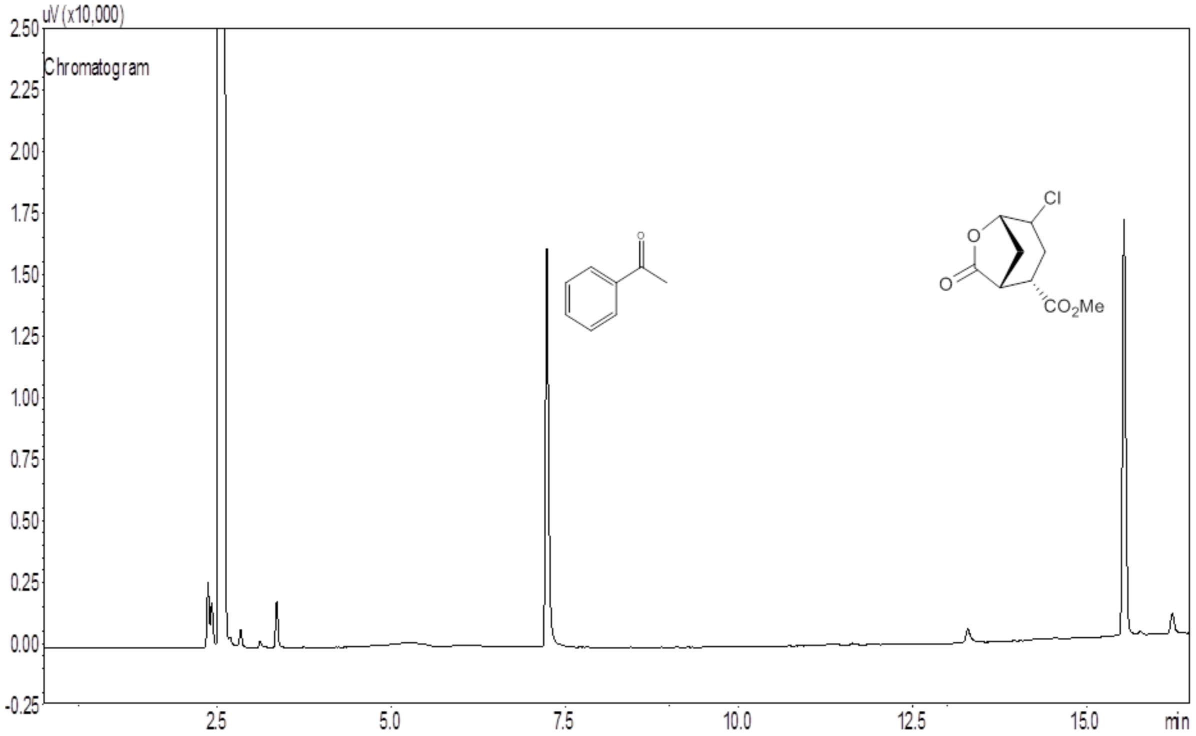

Figure S16. Representative GC chromatogram of methyl (1R,2R)-4-chloro-7-oxo-6-oxabicyclo[3.2.1]octane-2-carboxylate **(16b)**.

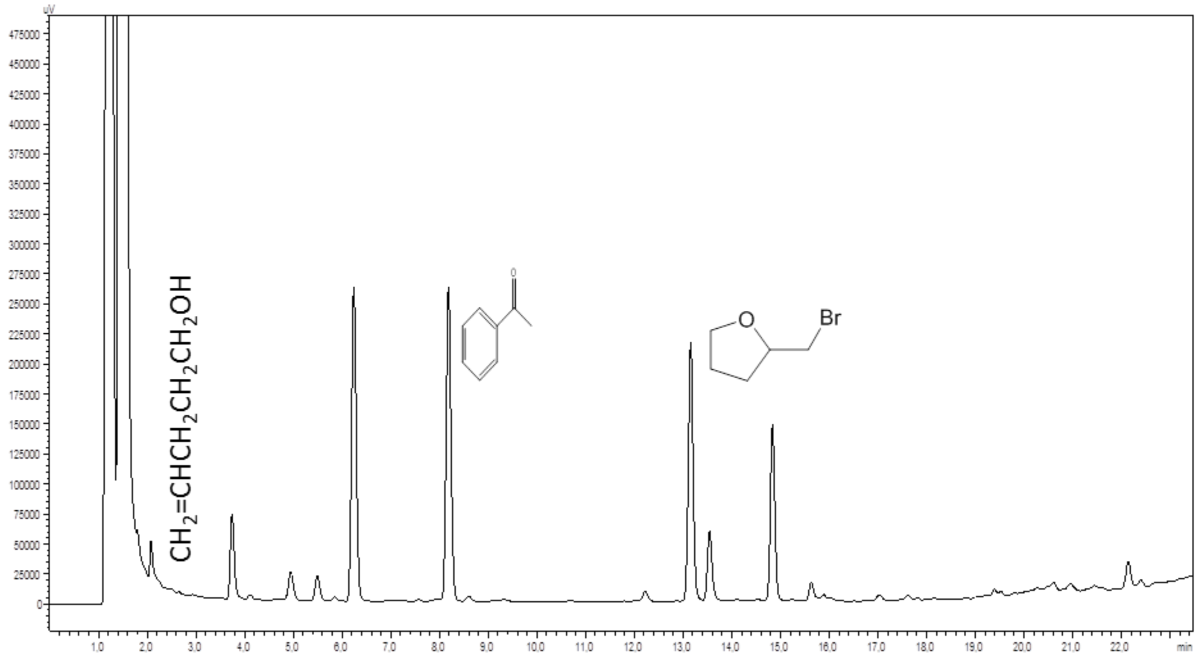

Figure S17. Representative GC chromatogram of 2-(bromomethyl)tetrahydrofuran **(17a)**.

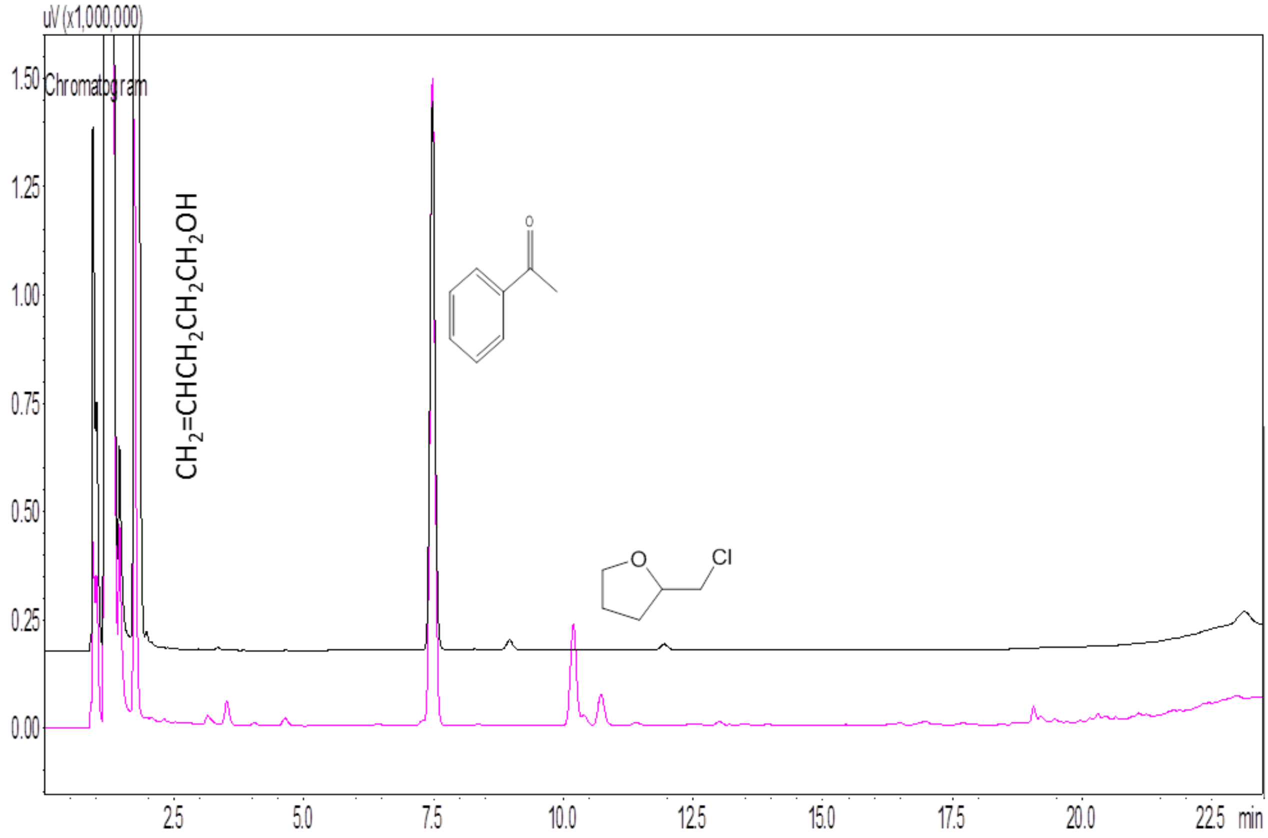

Figure S18. Representative GC chromatogram of 2-(chloromethyl)tetrahydrofuran **(17b)**.

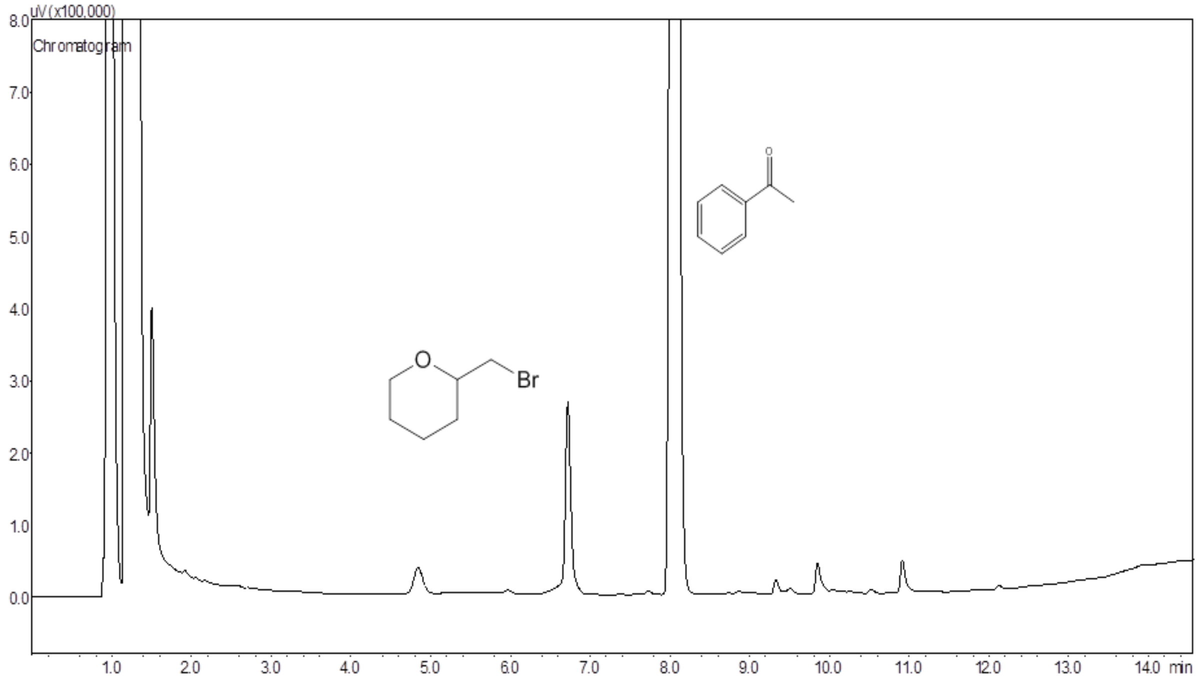

Figure S19. Representative GC chromatogram of 2-(bromomethyl)tetrahydro-2H-pyran **(18a)**.

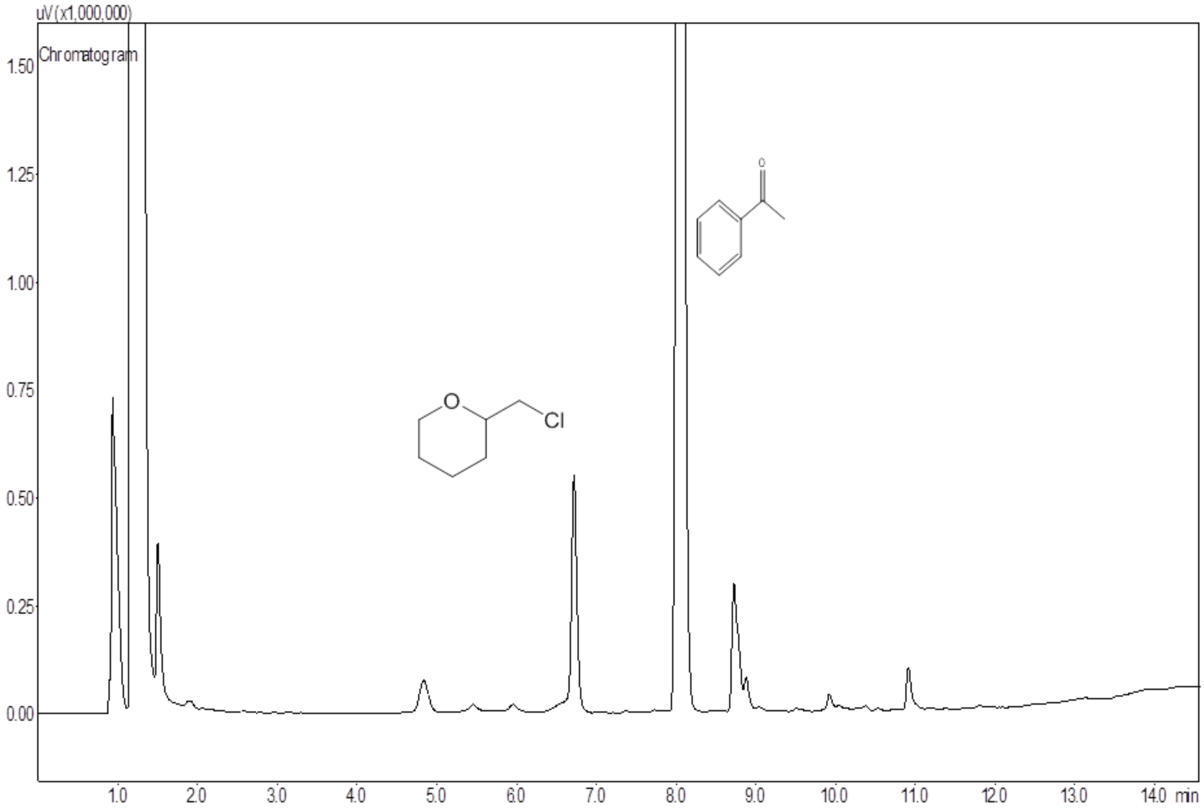

Figure S20. Representative GC chromatogram of 2-(chloromethyl)tetrahydro-2H-pyran **(18b)**.

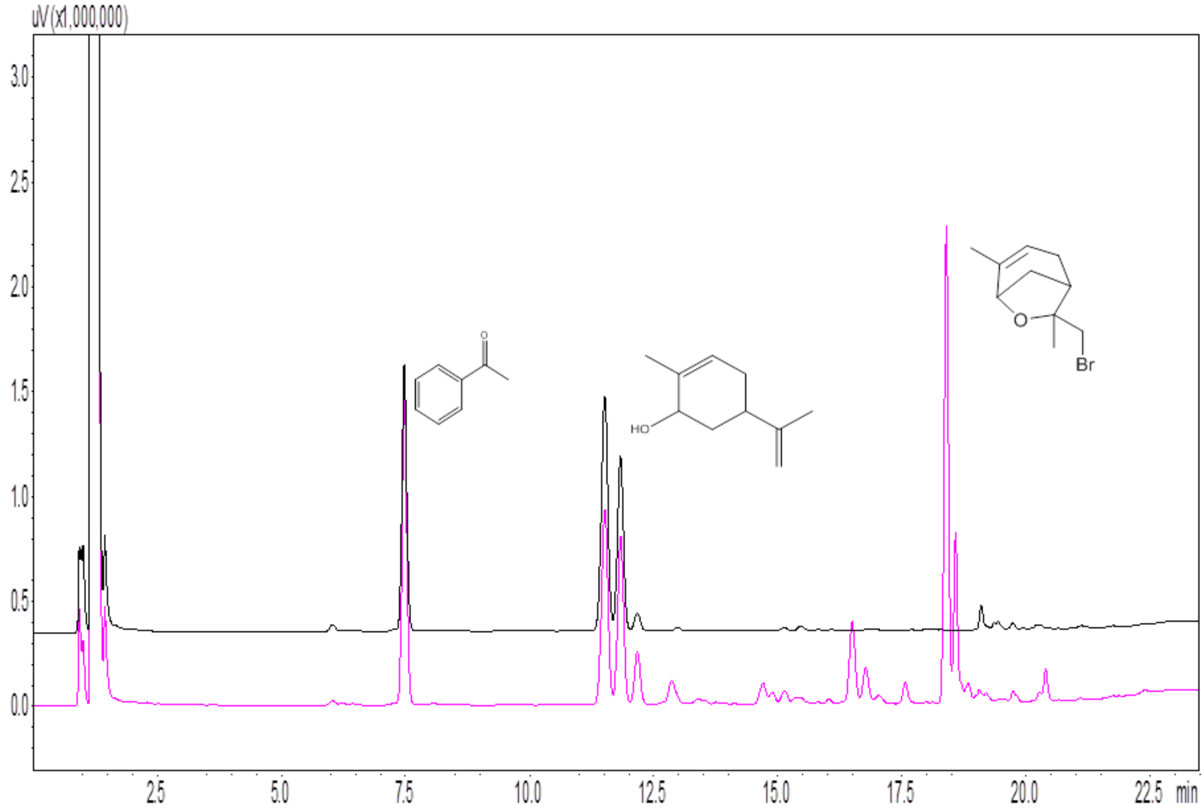

Figure S21. Representative GC chromatogram of 7-(bromomethyl)-4,7-dimethyl-6-oxabicyclo[3.2.1]oct-3-ene **(19a)**.

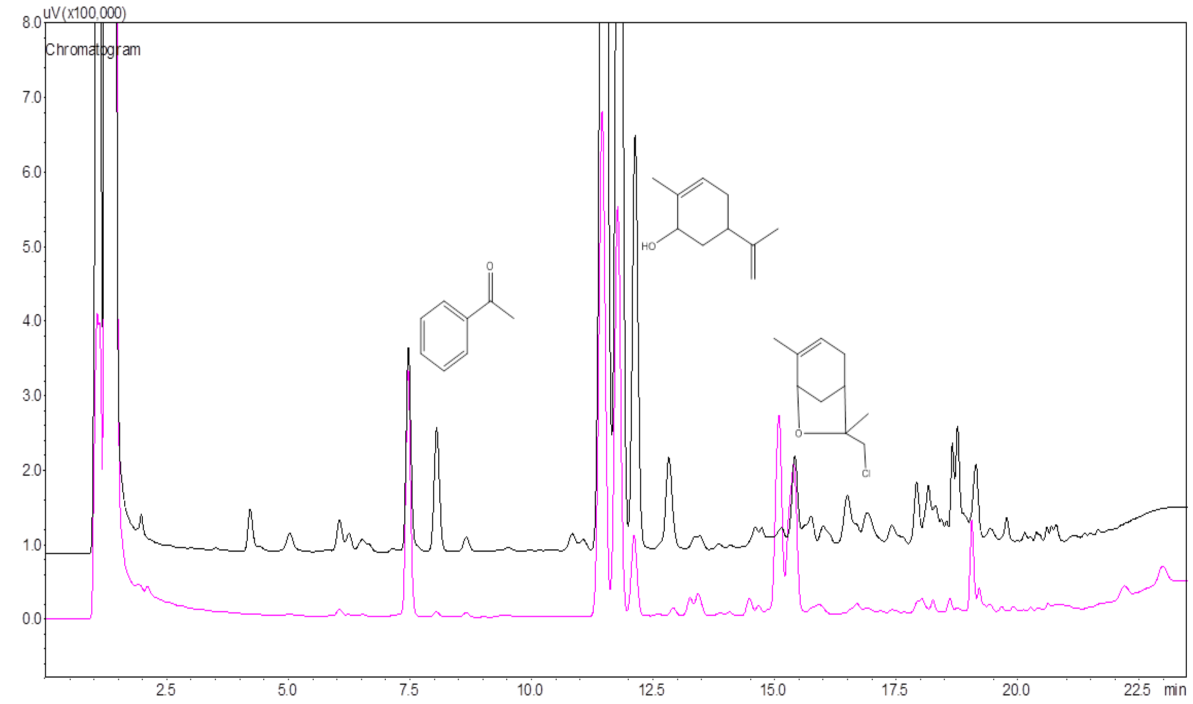

Figure S22. Representative GC chromatogram of 7-(chloromethyl)-4,7-dimethyl-6-oxabicyclo[3.2.1]oct-3-ene **(19b)**.

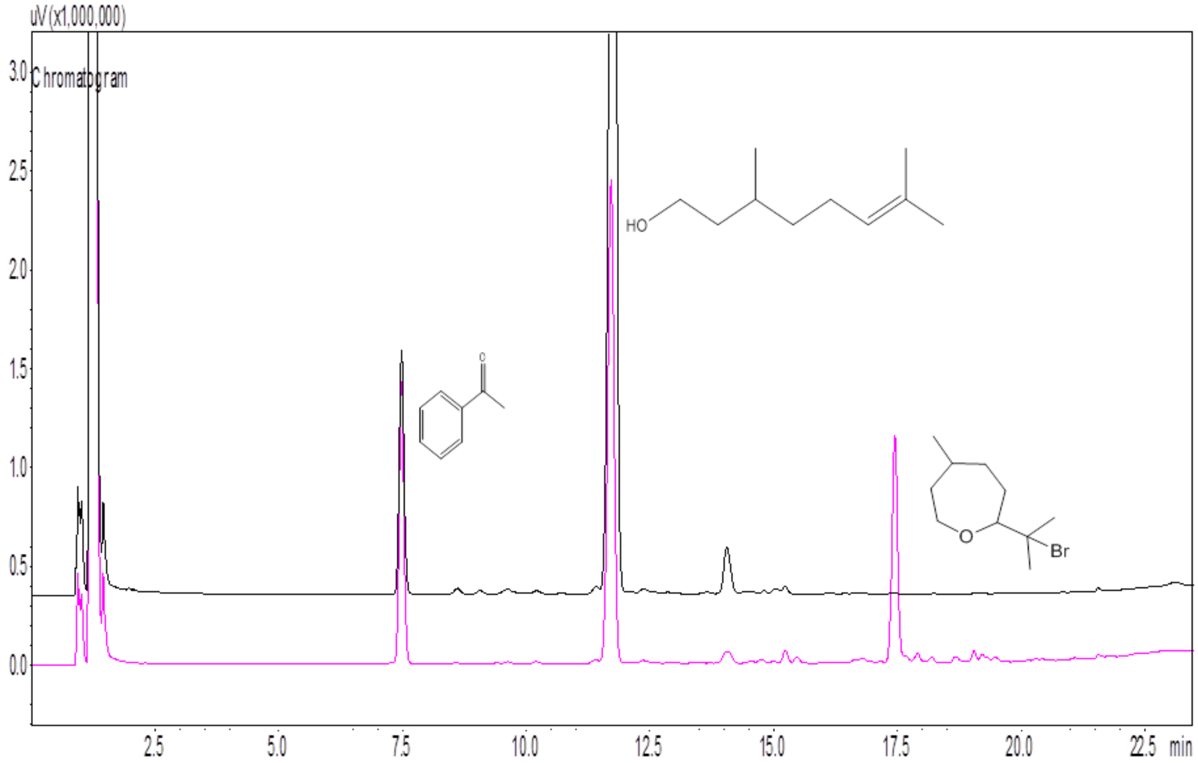

Figure S23. Representative GC chromatogram of 2-(2-bromopropan-2-yl)-5-methyloxepane **(20a)**.

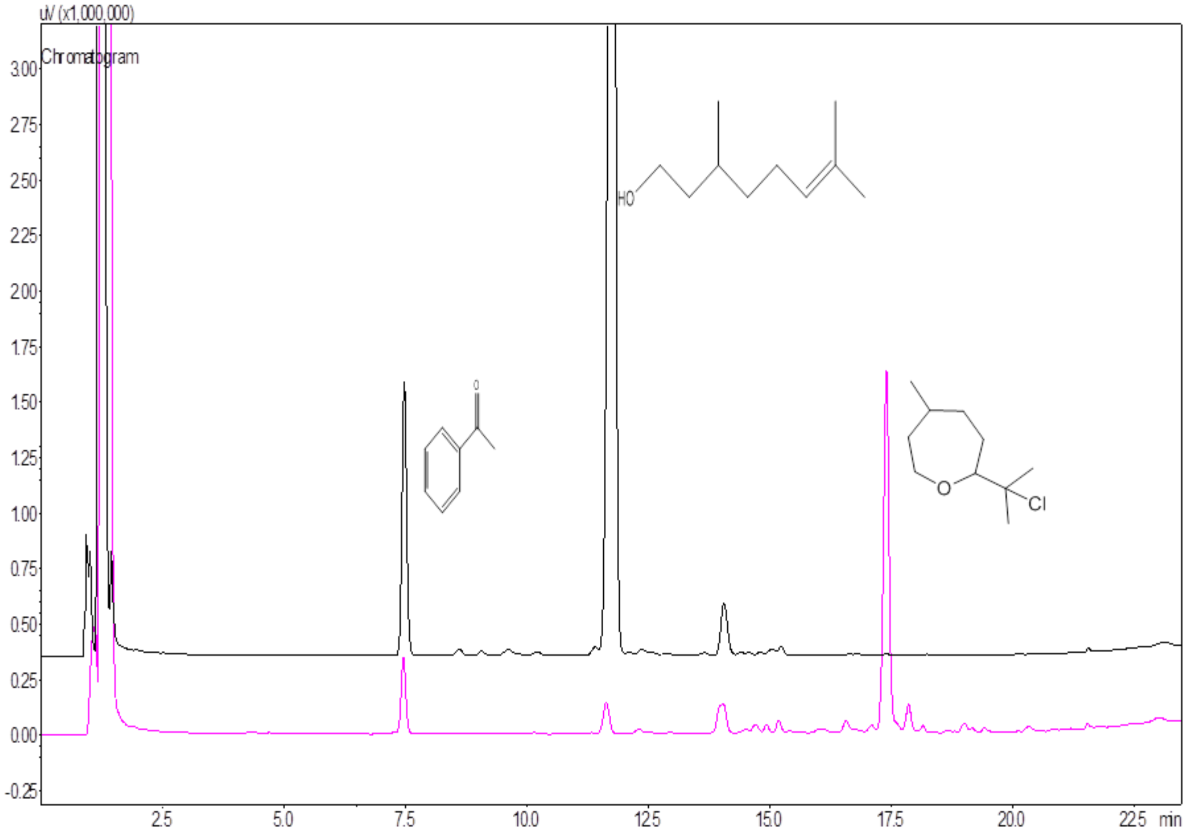

Figure S24. Representative GC chromatogram of 2-(2-chloropropan-2-yl)-5-methyloxepane **(20b)**.

7.6. Kinetic Resolution (KR) Chromatograms

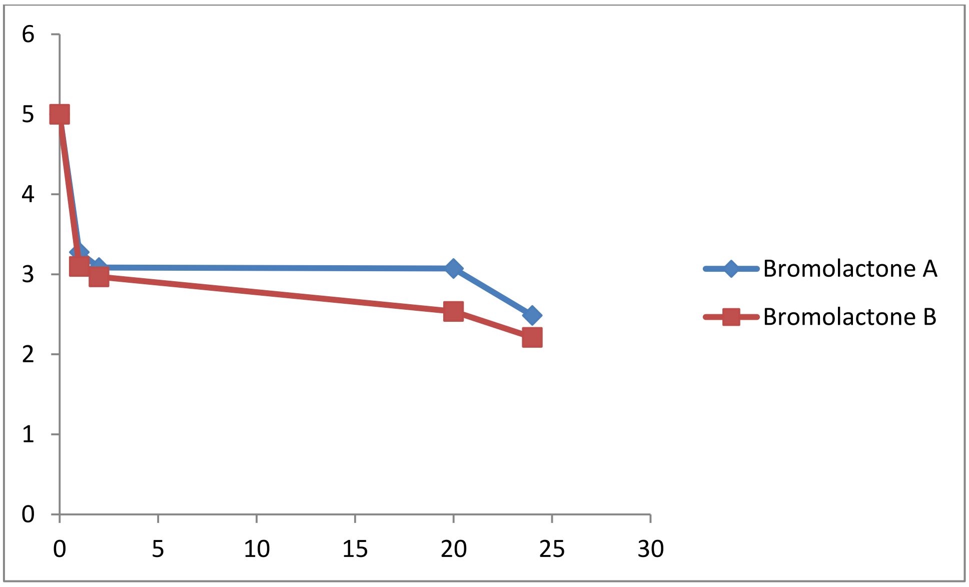

Figure S25. Representative kinetic resolutions chromatogram of bromolactone & Lipase porcine pancreas (PPL). Conditions: c(bromolactone) = 10 mM; Lipase porcine pancreas (PPL)= 10 mg; 100 mM phosphate buffer (KPi, pH 7.5); T = 30 ºC, t = 24h.

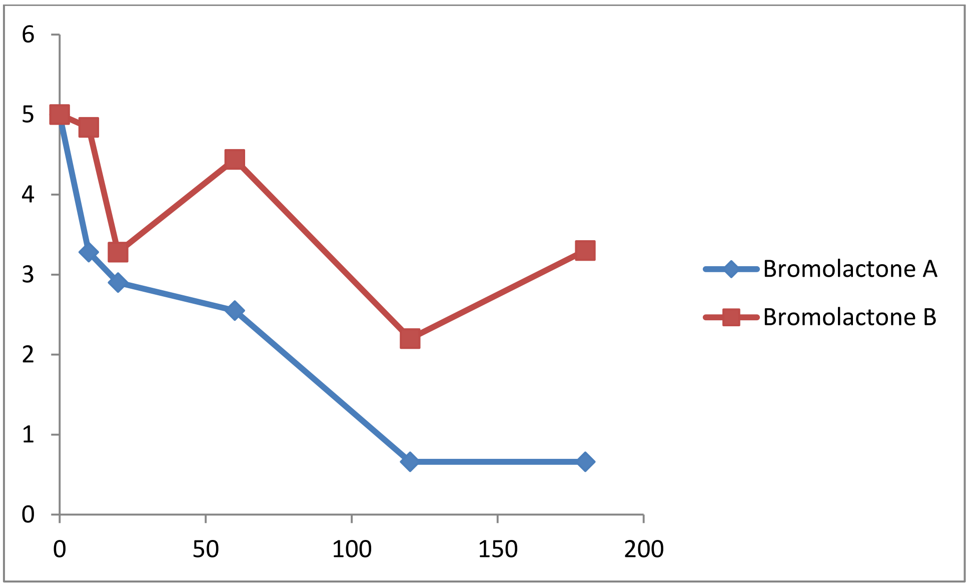

Figure S26. Representative kinetic resolutions chromatogram of bromolactone & (Lipase MAS1). Conditions: c(bromolactone) = 10 mM; MAS1 lipase= 1 mg; 100 mM phosphate buffer (KPi, pH 8.0); T = 40 ºC, t = 180 min.

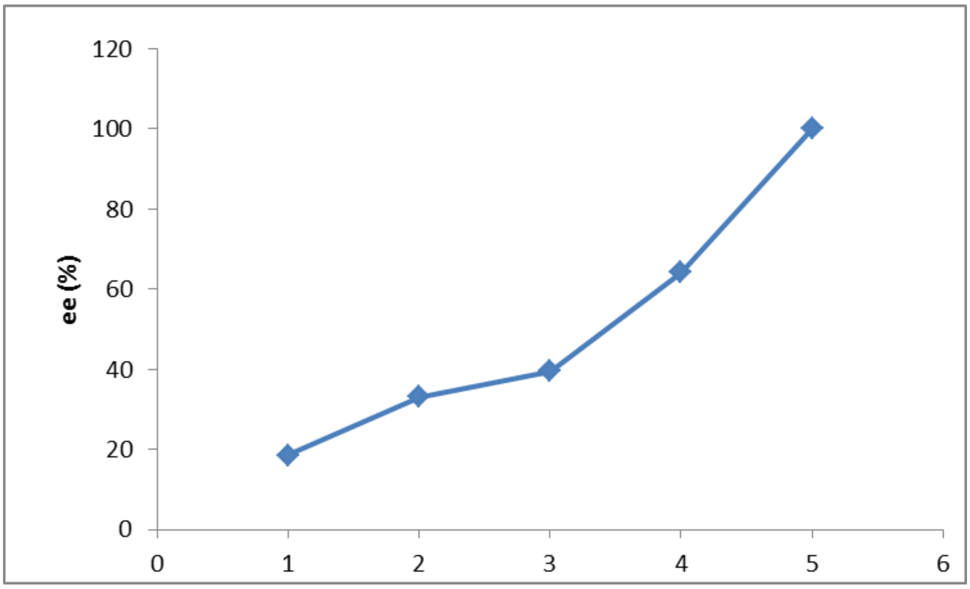

Figure S27. Representative ee value of the kinetic resolutions of bromolactone.
